# Genomic analysis of jumbo coliphage fEgEco12

**DOI:** 10.1007/s00705-026-06623-3

**Published:** 2026-04-12

**Authors:** Shimaa Badawy, Mikael Skurnik

**Affiliations:** 1 Department of Bacteriology and Immunology, Medicum, Human Microbiome Research Program, Faculty of Medicine, University of Helsinki, Helsinki, 00014 UH Finland; 2 Department of Botany and Microbiology, Faculty of Science, Damietta University, New Damietta, 34511 Egypt

## Abstract

Avian pathogenic *Escherichia coli* (APEC) infections are associated with major economic losses, cause perihepatitis, pericarditis, septicemia and even systemic infections in the poultry industry. APEC infections have usually been controlled by antibiotics, resulting in an increased prevalence of antibiotic-resistant *E. coli*. Concerns have been increased that transfer of antibiotic-resistant APEC via the food chain may cause risks for extra-intestinal infection of humans related to zoonotic transfer and increased difficulties in the treatment of human infections caused APEC-related *E. coli* types. With the occurrence of antibiotic resistance reaching a crisis point, it is important to find alternative treatments for multidrug-resistant infections. The use of phages to control pathogens is a promising therapeutic option for antibiotic replacing, therefore, novel phages specific for APEC were isolated in this study. Phage fEgEco12 was isolated from the hospital sewage water sample during a search for candidates for phage therapy applications against APEC. The phage morphology was studied by transmission electron microscopy, and the host range was analyzed with a Bioscreen C analyser. The phage genomic DNA was isolated, sequenced and annotated. Phage stability, phage adsorption and one step growth curve experiments were carried out to characterize its biological properties. Genomic analysis revealed that fEgEco12 is a lytic jumbo phage with genome size of 374,733 bp encoding 670 predicted genes. No genes associated with lysogeny, bacterial virulence, or antibiotic resistance were identified. The phage had myovirus morphology and a narrow host range. Comparative genomic analysis revealed that fEgEco12 and phage Ecwhy_1 belong to a new species in jumbo phage genus *Asteriusvirus*. fEgEco12 is a novel jumbo bacteriophage that is considered safe for phage therapy.

**Supplementary Information:**

The online version contains supplementary material available at 10.1007/s00705-026-06623-3.

## Background

*Escherichia coli* strain #6581 is an avian pathogenic *E. coli* (APEC). There are genetic similarities between the APEC and UPEC (uropathogenic *E. coli*) pathotypes, suggesting that APEC strains could be a potential source of virulence and antimicrobial-resistance genes for the UPEC strains [1]. *E. coli* is a well-known pathogen that is divided into two major groups: diarrheagenic *E. coli* (DEC) and extraintestinal pathogenic *E. coli* (ExPEC). The DEC strains are responsible for gastrointestinal infections, while ExPEC strains are responsible for diseases outside the intestinal tract such as sepsis, urinary tract infections and meningitis [2, 3]. APECs are ExPEC strains and are responsible for causing colibacillosis in poultry [4]. Avian colibacillosis refers to any systemic or localized infection caused by an APEC strain, and it is also an economically important disease that threatens food safety and avian welfare worldwide [1]. Avian colibacillosis can be treated with antibiotics. However, antibiotic resistance is increasing, and treatment has been very complicated due to the rise of both the number of antibiotic-resistant strains and the considerable prevalence of antibiotic-resistance mechanisms [5, 6]. The number of multidrug-resistant *E. coli* strains has increased considerably in the last decade, limiting treatment options in both humans and animals [7, 8]. Various studies have reported the isolation, characterization, and application of bacteriophages targeting APEC strains of poultry origin. Kazibwe et al. isolated and characterized seven lytic bacteriophages active against APEC strains recovered from broiler chickens. Such phages exhibited moderate host ranges and showed limited thermal stability, losing infectivity at temperatures above 60 °C [9]. Similarly, Tang et al. reported the characterization of a lytic bacteriophage, CE1, which had shown promising potential as a surface disinfectant, with therapeutic efficiency in control of APEC infection in vivo by using a 1-day-old broiler chicken challenge model [10]. Smith et al. isolated and characterized seven distinct phages (ASO1A, ASO1B, ASO2A, ASO78A, ASO2B, AVIO78A, ASO78B) directed against the APEC serogroups O1, O2, and O78. The phages belonged to multiple genera [e.g., *Felixounavirus*,* Phapecoctavirus*,* Tequatrovirus*) with variable host ranges against APEC strains. Phage ASO78A, for example, lysed five of the six tested strains in vitro. None of the phages carried virulence, antimicrobial resistance, or lysogeny genes, hence supporting their potential safety for therapeutic use [11]. Yao et al. isolated the lytic phage PEC9 from chicken farm feces that was able to lyse strains of the two major APEC serotypes, O1 and O2, in vitro. Crucially, in vivo mouse models demonstrated improved survival, reduced bacterial loads, and reduced organ damage following APEC challenge when treated with PEC9, hence supporting therapeutic potential [12].

Bacterial viruses, bacteriophages (or phages), are the most abundant microorganisms in the environment [13], and play crucial roles in microbial ecology and evolution. Phages can be used as an alternative treatment in therapy against bacterial infections, especially those caused by multi-drug resistant (MDR) bacteria [14, 15]. Compared with the high-cost and lengthy development of novel antibiotics, it is relatively economical and fast to isolate new phages and to promote the research of phages as antibacterial agents [16]. It is estimated that about half of the bacterial cells in the environment are killed by phages every day [17]. Upon completion of the lytic reproductive cycle, the phage-infected bacterial cells undergo lysis that leads to the release of newly formed progeny phages into the environment. Thus, phages have been used as a possible strategy for controlling bacterial infections in several areas, where multi-resistant pathogenic strains of *E. coli* represent one of the leading agents associated with diseases [16].

Large icosahedral viruses that infect bacteria represent an extreme of the coevolution of capsids and the genomes they coordinate. One group of these large viruses is the jumbo phages, tailed phages with double-stranded DNA genomes with a genome size > 200 kilobases (Kb) [18]. The jumbo phages, specifically that infect *E. coli*, are of significant interest due to their complex genetic structures and potential applications [19]. In addition, some of them have been found to encode nucleus-like structures that protect their genomes from the DNA-targeting bacterial defenses [20–22]. Thus, they hold great promise in the field of phage therapy against bacterial infections [23, 24].

These phages are assigned “jumbo” because of their most notable features of a large phage virion and large genome size. However, in addition to these, jumbo bacteriophages also display several novel characteristics which have not been observed for phages with smaller genomes, that differentiate jumbo phages in terms of genome organization, virion structure, progeny propagation, and evolution [25]. Additionally, large viruses can show specific structures like long, wavy and curly tail fibers, which have rarely been observed in smaller phages [26], and their large genomes encode DNA polymerases, RNA polymerases, endolysins, chitinases, glycoside hydrolases, lyases and many other genes with unknown functions [25]. Most isolated jumbo phages infect Gram-negative bacteria, have long life cycles and diffuse poorly in soft agar, thus producing tiny plaques that can be difficult to detect [27].

In this study, we isolated and characterized a novel *Escherichia* jumbo phage fEgEco12, with a myovirus morphology, characterized by its long, contractile tail [28], classified as “giant” due to its exceptional size, encapsulating a huge, densely packed DNA genome of 374,733 bp.

## Materials and methods

### Bacterial strains, phage isolation and purification

The bacterial strains used in this study are listed in Supplementary Table S1. *E. coli* strain #6581 was used as the host bacterium for phage isolation. All bacteria and phage incubations were performed at 37 °C using Lysogeny Broth (LB). LB agar (LA) plates were supplemented with 1.5% agar, and soft agar medium included 0.4% agar [29].

A sewage sample from cancer clinic, Helsinki University Central Hospital, Helsinki, Finland, was used as the source for the phage isolation. The sewage sample was centrifuged at 8,000 rpm for 5 min and the supernatant was sterilized using a 0.22 μm syringe filter (Millipore, USA). The phage lysates were produced in liquid cultures with 50 µl of phage suspension and 500 µl of overnight bacterial culture added to 9 ml LB and incubated at 37 °C with vigorous agitation for 6 h or until lysis occurred. 200 µl of chloroform was added to each 3 ml of lysate to kill remaining bacteria, and the mixture was incubated at room temperature for 20 min by gently turning the tube up and down. The lysate was then centrifuged at 5,000 rpm for 10 min or until the supernatant was clear. The supernatant was filtered through a 0.2 μm syringe filter. To stabilize the phage during long-term storage, sucrose was added to 8%. Filtered phage lysate in LB was used for the assays unless otherwise stated [27].

To titrate phage lysates, 100 µL of an overnight culture of *E. coli* strain #6581 and 50 µL of 10-fold diluted phage samples were added to 5 mL of LB soft agar and overlaid on LB agar. After incubation overnight at 37 °C, the number of plaques was counted and expressed as plaque-forming units per milliliter (PFU/mL), and a single plaque was selected and resuspended in 1× SM buffer (100 mM NaCl, 10 mM MgSO4, 50 mM Tris–HCl, pH 7.5). Resuspended phage was filtered through a 0.22 μm syringe filter to remove bacterial debris. Subsequently, the phage was isolated using three rounds of plaque purification [30], the isolated phage was stored at −80 °C in LB broth containing 20% glycerol until further use [31, 32].

### Transmission electron microscopy

Transmission electron microscopy analysis was performed at the Electron Microscopy Unit (Institute of Biotechnology, University of Helsinki, Helsinki, Finland). A purified high-titer phage fEgEco12 lysate was prepared in 0.1 M ammonium acetate, pH 7.0. A 3 µl aliquot of the phage preparate was transferred on a carbon-coated copper grid and allowed to absorb for one minute and then the grid was negatively stained using 2% uranyl acetate for 30 s and visualized using the transmission electron microscope (JEOL JEM-1400, Tokyo, Japan) under 80 kV equipped with a Gatan Orius SC 1000B bottom-mounted Charged Coupled Device-camera (Gatan Inc., Pleasanton, CA, USA). The size dimensions of fEgEco12 were measured from a minimum of ten separate phage particles [33].

### Phage DNA extraction, genome sequencing, assembly and bioinformatics analysis

Phage DNA was isolated from high titer phage preparation (10^12^ PFU/mL) using the phenol-chloroform extraction method [34]. Briefly, to 400 µL of the phage suspension, 1.3 µL DNase I (1 U/µL, Promega, Madison, WI, USA), and 4 µL RNase A (1 mg/mL) were added and the mix was incubated for 30 min at 37 °C to degrade bacterial DNA and RNA. Then, 16 µL of 0.5 M EDTA, 1.2 µL of proteinase K (20 mg/mL), and 20 µL 10% sodium dodecyl sulphate (SDS) were added to the tube that was incubated at 56 °C for at least 1 h. The cooled suspension was extracted sequentially with a volume of phenol, phenol/chloroform (1/1) and chloroform, each time mixing the tube gently for 15 min, followed by 5 min centrifugation at 16,000× g. The nucleic acid was precipitated from the final aqueous phase by adding 0.1 volumes of 3 M sodium acetate pH 5.2, and 2 volumes of absolute ethanol. The tube was mixed manually for 2–3 min until the precipitated DNA thread became visible and could be transferred using a 1 µL inoculation loop into a tube containing 1 mL 70% EtOH. After 10 min centrifugation at room temperature (RT), the supernatant was carefully removed and the pellet was air dried and dissolved overnight into 50–100 µL of TE buffer (10 mM Tris-HCL, 1 mM EDTA, pH 8.0). In the case that the DNA thread failed to form, the DNA was pelleted by centrifugation and washed with 1 mL of 70% EtOH as above. The quality and quantity of the DNA was estimated using the NanoDrop spectrophotometer (ND-1000, Wilmington, DE, USA) and/or the Qubit machine (Invitrogen Qubit 2.0 Fluorometer, CA, USA) applying the QubitTM dsDNA BR Assay Kit (Thermo Fisher Scientific), followed by visualization by agarose gel electrophoresis [34].

The genomic DNA of the phage was sequenced on an Illumina HiSeq platform at Novogene (Cambridge, UK), using a 150-bp paired-end protocol. The obtained sequence reads were *de novo* assembled using the A5-miseq pipeline [35]. To verify the fidelity of the assemblies, the original reads were mapped back to the de novo assembled contigs using the Geneious Prime tools (R10 software version 2023.2, Biomatters Ltd., Auckland, New Zealand) [36]. Preliminary annotation of the phage genome was carried out using rapid annotation subsystems technology (RAST) [37] and the gene prediction annotations were manually checked and revised using the Artemis software (release 2.30.0) [38]. The PhageTerm program was used to identify the termini and packaging method of the phage genome [39]. The identities and functions of the predicted genes and protein annotations were analyzed using the BLASTP (https://blast.ncbi.nlm.nih.gov/Blast) and HHpred [40] servers. To study the similarity of fEgEco12 to known phages, the whole genome was first analyzed with Microbial Nucleotide BLAST, and a number of most similar phage genomes were then selected for phylogeny analysis. To determine the phylogenetic position of phage fEgEco12 homologous protein sequences of the major capsid protein were identified from the most closely related bacteriophages using BLASTp and the phylogenetic analysis was performed using MEGA version 11 [41] with default parameters (CLUSTAL W aligner and Fast Tree plugin using the neighbor-joining statistical tree analysis method with 100 bootstrap replications). A heatmap integrating the intergenomic similarity values and information regarding the genome lengths and the aligned genome fractions was prepared using the Virus Intergenomic Distance Calculator (VIRIDIC) program [42]. The alignment of the genomes of selected jumbo phages was performed using the progressive Mauve [43]. The phage fEgEco12 and Ecwhy_1 proteomes were compared using the Proteome Comparison tool of the BVBRC platform (https://www.bv-brc.org/) which performs protein bidirectional sequence-based comparison using BLASTp. The linear genomic map of phage fEgEco12 was generated using the Linear Genome Plot tool [44] at Galaxy Europe (https://usegalaxy.eu/).

### Phage host range determination

A total of 147 bacterial strains, including 132 *E. coli*, 10 *Staphylococcus aureus*, two *Pseudomonas aeruginosa*, two *Acinetobacter baumannii*, and one *Klebsiella pneumoniae* strains (Supplementary Table S1) were used to assess fEgEco12 host range. The host range screening was done with a liquid culture method using Bioscreen C analyser (GrowthCurves AB Ltd, Finland) absorbance plate reader. The assay was performed as previously described with slight modifications [45]. Ten µL of phage lysate (10^9^ PFU/mL) was added to the honeycomb plate wells containing 100 µL of aliquots of 1:500 diluted overnight selected bacterial cultures. A negative control containing bacteria but no phage, a positive control with *E. coli* host strain #6581 and phage, and a blank control containing only LB were included to each plate. The samples were analyzed as duplicates. Plates were incubated at 37 °C with continuous shaking and the growth was monitored for 16 h with 1 h measurement intervals at OD_600_. The mean optical density and standard deviation (SD) were calculated for each sample at each time point. Bacterial growth curves were plotted as OD_600_ readings versus time. The blank was subtracted from all samples, and the means were calculated from the two parallel samples. fEgEco12 was considered to efficiently infect a given bacterial strain if the absorbance of the culture containing phage and bacteria was below 70% of the negative control.

### Restriction enzyme digestions

The purified phage fEgEco12 DNA was digested with the restriction endonucleases EcoRI, NsiI, SpeI, SmaI, SexAI, PshAI, CIaI, AflII, NruI and HindIII that in silico analysis were predicted to produce best resolved restriction fragment patterns. The restriction enzyme digestions were carried out according to the manufacturers’ instructions in a final volume of 10 µL. Briefly, 1.0 µL of phage DNA (approximately 300–600 ng/µL), 1.0 µL of 10 × digestion buffer, 0.5 µL of the restriction enzyme and nuclease free water (7.5 µL) were mixed in a microtube for each enzyme and incubated at 37 °C for 2 h. The samples with the gene ruler were then loaded into wells of 1% (w/v) agarose gel supplemented with 0.005% (w/v) Midori green dye. The DNA fragments were separated by running the samples at 65 V, 200 mA for 180 min. Then, the restriction fragment bands were visualized using the BioRad GelDoc XR+ imaging system.

### Phage stability

The stability of phage under different conditions, including temperature and pH, was evaluated using previously described methods [46, 47], with few modifications. To test the temperature stability of the phage, 100 µl of phage suspension (10^9^ PFU/mL) was incubated at temperatures ranging from 4 °C to 80 °C for 1 h, followed by determining phage titer and viability using the double-layer agar (DLA) method at each temperature. A bacteriophage suspension kept at 4 °C was used as a control in this study. The phage stability at different pH values was evaluated by mixing 10 µl of concentrated phage suspension (10^9^ PFU/mL) with 990 µl of SM buffer adjusted to various pH values ranging from 1 to 14. The mixture was then incubated at 37 °C for 1 h, and then the phage titer was determined by measuring the number of phage plaques using the DLA method.

### Phage adsorption assay

The overnight host bacterium was sub-cultured to 5 mL of LB and incubated at 37 °C until OD_600_ ∼1.0. Then the bacterium was pelleted by centrifugation at 5000 rpm for 10 min and resuspended into 0.9 ml of fresh LB. Thereafter, 100 µl of fEgEco12 (3 × 10^9^ PFU) was mixed with 900 µl of fresh host bacterial culture of (10^9^ CFU/ml) corresponding to multiplicity of infection (MOI) of 1 (the experimental tube A). The control tube B contained 0.9 mL of fresh LB medium without bacteria. The suspension was incubated at 37 °C, 120 rpm for 30 min and samplings of 50 µl aliquots were done at 5 min intervals from tubes A and B and dispensed into pre‐chilled Eppendorf tubes. The samples were briefly vortexed then centrifuged at 13,000 × g at + 4 °C for 3 min and the supernatants were recovered. The numbers of free phages in 50 µl of the supernatants were determined by DLA method. Briefly, 50 µL of the supernatant was added to 200 µL host bacteria in 3 mL molten soft agar media tubes previously maintained at 50 °C. The mixtures were briefly vortexed, dispensed on pre-warmed LB agar plates and allowed to set (solidify) at RT. After overnight incubation at 37 °C the number of plaques were counted from all the plates and recorded at their respective time points (from 0 min to 30 min). Plaque counts from control tubes (tube B) were used as time point 0 min reference points. The titrations were performed in triplicates. LB was used as an adsorption-free control without bacteria. The values were normalized by having the average PFU of tube B representing 100%. The adsorption rate constants (k‐values) were calculated for 5 min time points as described [48].

### One step growth curve

In the one-step growth curve experiment, an exponentially growing culture of *E. coli* strain #6581 (ca. 10^6^ CFU/mL) was infected with fEgEco12 phage at a MOI of 0.01. The one step growth curve experiment was carried out as described [49]. Phage titers were determined using the DLA method in triplicate from the 1/10, 1/100, and 1/1000 diluted cultures at 5 min intervals. The 1/10 diluted culture was sampled from 5 to 30 min, the 1/100 diluted culture from 35 to 45 min, and the 1/1000 diluted culture from 50 to 60 min. The obtained PFU/mL values were used to draw the growth curve and to calculate the burst size.

## Results and discussion

### Isolation and morphological analysis of phage fEgEco12

**Fig. 1:**
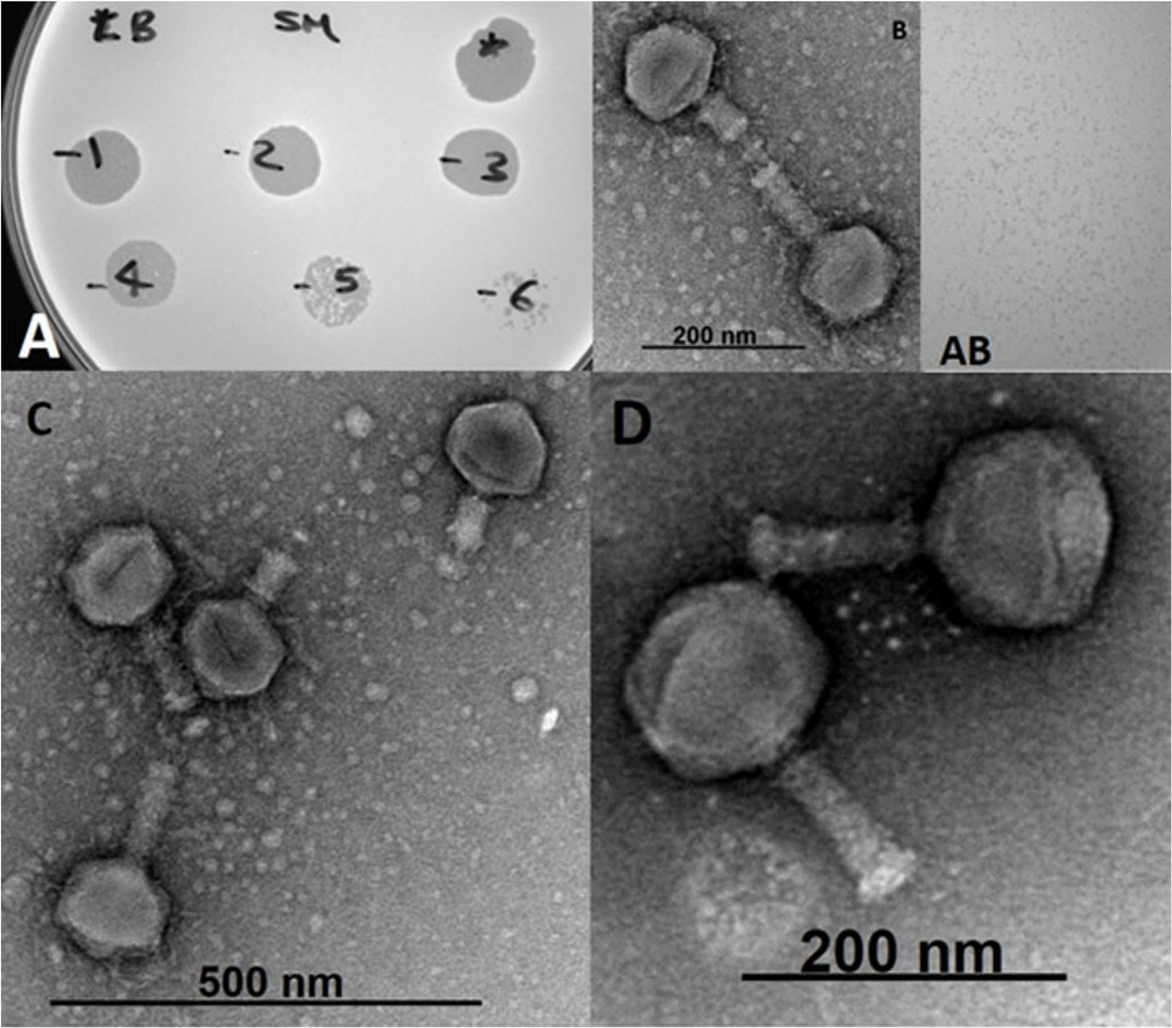
Properties of phage fEgEco12 isolated from sewage water. (**A**) The lytic activity of the phage by spot test. (AB) Clear and rounded plaques with a diameter ~ 1 mm. (**B**, **C**, **D**) Transmission electron micrographs of fEgEco12 with negative staining. Short tail fibers (arrows) on the base plate are clearly visible (**D**)

The *Escherichia* phage fEgEco12 was isolated from the hospital sewage water sample during a search for candidates for phage therapy applications. Phage fEgEco12 formed relatively clear and small (0.5–1 mm) plaques against *E. coli* #6581 (Fig. 1A). Transmission electron microscopy (TEM) analysis revealed that phage fEgEco12 has jumbo phage dimensions and indicated that the phage is a member of the *Caudoviricetes* class with an icosahedral head and contractile tail (Fig. 1B, C and D). The average total length of the non-contracted phage particle was 237 ± 2 nm and 187 ± 2 nm for a contracted particle without tail tube. The tail diameter of the contracted tail sheath was greater than that of the non-contracted tail. The head was very symmetrical for a non-contracted particle but for a contracted particle the head was wider than it was long (Table 1). The neck connected the head to the tail and the tail ended in a baseplate. Phages have a long contractile tail with unique hairy fibers which may be involved in host interaction [50].

**Table 1:** The dimensions of fEgEco12 with contracted and non-contracted tails (all values in nm)

Tail status	Head to tail	Tail sheath length (w/o baseplate and neck)	Tail sheath diameter	Head height	Head width
Non-contracted	237 ± 2	109	39.1	130	126
Contracted	187 ± 2	65.2	47.8	121 0.7	130

### Bioinformatics analysis and genomic characterization of fEgEco12 genome

The genome of the *Escherichia* phage fEgEco12 was fully sequenced, annotated, and deposited in the GenBank database under the accession number PP777464.1. To further characterize fEgEco12, the sequenced genome was analyzed using various bioinformatics tools. The phage fEgEco12 genome has a 374,733 bp, linear, double stranded DNA molecule encoding 670 predicted genes with a G + C content of 35.8%. PhageTerm identified 20,993 bp direct terminal repeats.

**Fig. 2:**
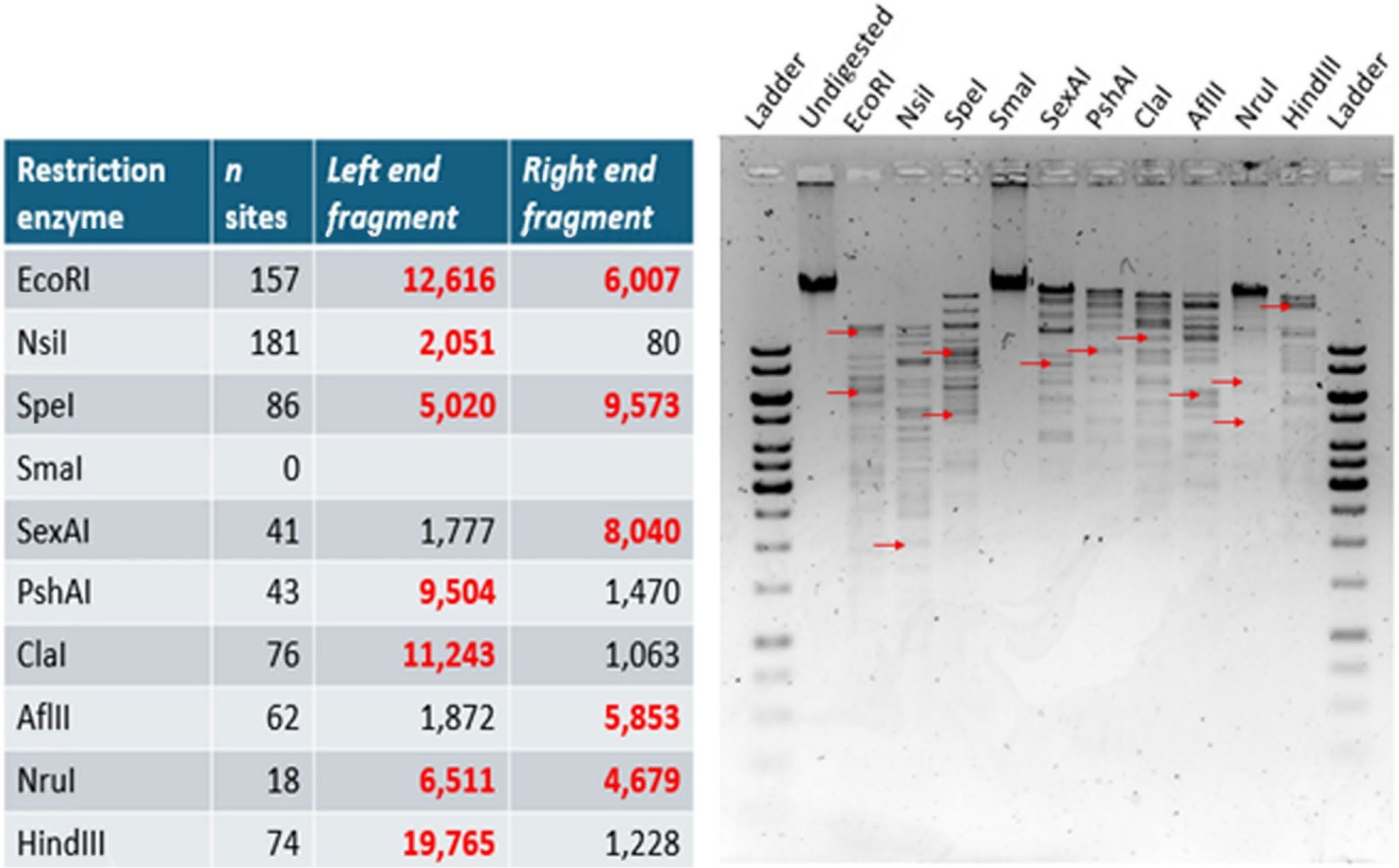
fEgEco12 restriction sites in silico vs. experimental digestions. The table lists the sizes of the predicted left and right end fragments (see supplementary Figures S2-S10 for details). The positions of the selected end fragments (in bold red) are indicated by red arrows in the agarose gel image

Restriction enzyme digestions were carried out to confirm the genome arrangement and the presence of the direct terminal repeats (Fig. 2 and Supplementary information Figures S2-S10). The restriction digestion fragments with all enzymes generated band patterns identical to those predicted in silico by NEB Cutter for the linear genome. The predicted left and right end restriction digestion fragments are shown in Fig. 2, and the positions of the fragments are indicated on the gel image. Overall, the restriction digestions confirmed the linear genome arrangement and the presence of the long terminal repeats of phage fEgEco12.

While the fEgEco12 genome is packaged in linear form into the heads of phage particles, it is very likely that the genome may be circularized within the infected cell to protect the free DNA ends against exonucleases and/or facilitate the replication process. The direct terminal repeats in many tailed class *Caudoviricetes* phages facilitate circularization via homologous recombination immediately after the genome enters the host cell before it starts the replication [51–53]. For example, the linear, terminally redundant DNA of phage P1 is known to circularize after entering the host and replicate as a plasmid like replicon [54]. Similarly, the linear genomes with direct terminal repeats use homologous recombination between the repeats to generate circular or concatemeric forms that act as replication templates before being processed into linear genomes for packaging [53]. The presence of the direct terminal repeats in the genome of phage fEgEco12 suggests that it is very likely circularized upon infection.

**Fig. 3:**
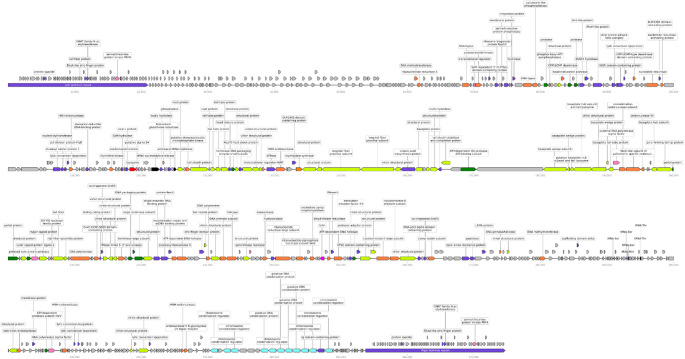
Linear genome map of phage fEgEco12 created using the Linear Genome Plot tool. The colors indicate the functional groups of the gene products. tRNA (black), structural proteins for capsid and tail formation (light green), packaging (dark green), DNA replication and repair-related proteins (orange), host lysis proteins (red), non-categorized proteins (violet) and hypothetical proteins (grey). Chromosome condensation associated proteins (turquoise)

Based on the bioinformatic analysis, 13 of the predicted genes were identified to encode virion structural proteins, and 170 genes, non-structural proteins with functions in replication, recombination, repair, translation and transcription. Thus, altogether 487 genes were annotated to encode hypothetical proteins or proteins of unknown function. The overall organization of the genome of fEgEco12 is presented in Fig. 3, and the annotation of the predicted genes is presented in Supplementary Table S2. tRNAscan identified 6 tRNA genes (two tRNA^**Ser**^ s, tRNA^**Met**^, tRNA^**Asn**^ and two tRNA^**Thr**^ s) located between bp 295,067 − 295,139. VirulenceFinder and ResFinder did not identify any known genes encoding virulence-, toxicity-, or antibiotic resistance-associated proteins. A functional annotation of the genome revealed that fEgEco12 encodes most DNA replication proteins, like two DNA polymerases, a clamp loader, primase/helicases, as well as a phage-encoded RNA polymerase sigma factor.

Furthermore, the fEgEco12 contains genes encoding putative lysis functions. These include the lysis proteins holin and the phage lysozyme. Notably, no genes encoding lysogeny-associated proteins or transposases were identified, and the in silico whole genome analysis indicated a 100% prediction of a virulent (lytic) phage lifestyle for fEgEco12. These results confirmed that fEgEco12 can be considered strictly lytic and suitable for therapeutic applications and the large capsid is able to contain the 374,733 bp-long viral DNA. It can thus be classified as a jumbo phage.

### Genomic comparison and phylogenetic analysis of phage fEgEco12

The phylogenetic proteomic tree of phage fEgEco12 to other described prokaryotic viruses was generated using the ViPTree that allowed to identify the closely related phages, demonstrating significant similarity among their genomes (Figure S1). The head diameters of the closely related jumbo phages are comparable to that of fEgEco12. For example, the head diameter of the avian pathogenic *Escherichia* virus phAPEC6 is 136 nm with the capacity to package its 352,598-bp genome [51]. Similarly, the head diameter of the 348.532 bp *E. coli* phage PBECO4 is 132 nm [55], and that of the 348,532 bp coliphage 121Q head, first measured as 116 nm [56], but corrected to 132 nm after cryo-electron microscopy and three-dimensional reconstruction [19]. The head diameter of the 345,809 bp *Klebsiella* phage vB_KleM-RaK is 123 nm [57].

**Fig. 4:**
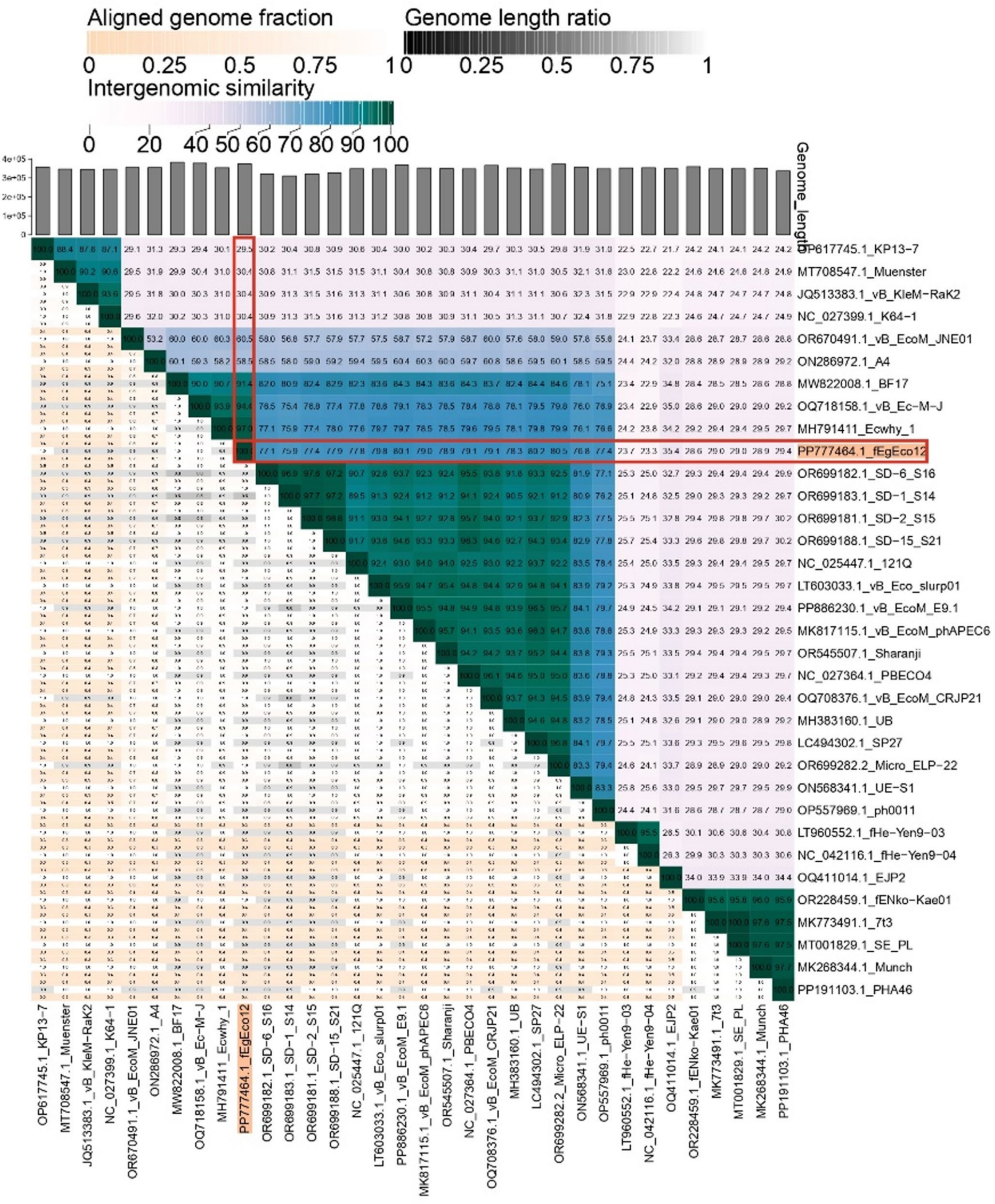
Whole-genome comparison and clustering of phage fEgEco12 (PP777464.1 indicated by red borders) with its close relatives (Table 2), carried out with VIRIDIC. In the top right corner of the heat map, different shades of blue indicate intergenomic similarity (%) between the genomes of each pair compared. Numerical values are also shown. The darker the color, the more closely related are the genomes. The lower left corner of the heat map shows three indicator values for each genome pair: top value, the aligned fraction of genome 1 for the genome in this row; middle value, the genome length ratio for the two genomes in this pair; bottom value, the aligned fraction of genome 2 for the genome in this column

The taxMyPhage tool (https://phagecompass.ku.dk/) classified fEgEco12 as a new species in the jumbo phage genus *Asteriusvirus* [58]. A set of jumbo phages was selected based on VipTree and BLASTn comparisons (Table 2). The VIRIDIC analysis generated a heatmap of pairwise intergenomic similarities to a set of 33 closest phage genomes of fEgEco12 (Fig. 4; Table 2). VIRIDIC quantifies genomic relatedness at nucleotide-level comparisons. Similarity values ≥ 95% indicate that the phages represent separate strains of a phage species consistent with established ICTV demarcation criteria, and similarity values > 70% classify the phages into same genus. Based on the VIRIDIC analysis the phages fell into 7 distinct genus clusters (Table 2) of which genus cluster 2 with 20 phages represents the genus *Asteriusvirus*. The closest relative of fEgEco12 was *Escherichia* phage Ecwhy_1 with 97.0% identity, and the identity to other *Asteriusvirus* phages ranged between 76 and 94%. On the other hand, fEgEco12 is ca. 30% identical to Klebsiella phages of genus *Alcyoneusvirus* (Genus cluster 1), 29% identical to the Salmonella phages of genus cluster 4, and ca. 23% identical to *Yersinia* phages of genus *Eneladusvirus* (genus cluster 3). Supporting metrics such as aligned genome fraction and genome length ratio, displayed alongside the heatmap, provide additional confidence in the inferred genomic affiliations and contribute to a comprehensive comparative analysis.

**Table 2:** Summary of the phage fEgEco12-related phages compared using the VIRIDC tool

Accession No	Phage	Genome size	Terminal repeat	Genus (NCBI)	ANI %	Species cluster	Genus cluster
PP777464.1	fEgEco12	374,733	20,993	Asteriusvirus	100	6	2
MH791411	Ecwhy_1	354,537		Asteriusvirus	97.0	6	2
OQ718158.1	vB_Ec-M-J	378,573	21,525	Asteriusvirus	94.4	19	2
MW822008.1	BF17	383,493		Asteriusvirus	91.4	10	2
LC494302.1	SP27	347,152		Asteriusvirus	80.2	2	2
OR699282.2	Micro_ELP-22	373,488		Asteriusvirus	79.8	2	2
PP886230.1	vB_EcoM_E9.1	367,851		Not defined	80.1	3	2
LT603033.1	vB_Eco_slurp01	348,043		Asteriusvirus	79.8	3	2
NC_027364.1	PBECO4	348,113		Asteriusvirus	79.1	12	2
OQ708376.1	vB_EcoM_CRJP21	366,301		Asteriusvirus	79.1	12	2
MK817115.1	vB_EcoM_phAPEC6	352,598		Asteriusvirus	79.0	8	2
OR545507.1	Sharanji	350,079		Asteriusvirus	78.9	8	2
MH383160.1	UB	353,081		Asteriusvirus	78.3	5	2
NC_025447.1	121Q	348,532		Asteriusvirus	77.8	11	2
OR699188.1	SD-15_S21	326,338		Asteriusvirus	77.9	21	2
OR699181.1	SD-2_S15	321,761		Asteriusvirus	77.4	21	2
OR699182.1	SD-6_S16	319,470		Asteriusvirus	77.1	21	2
OR699183.1	SD-1_S14	310,643		Asteriusvirus	75.9	21	2
OP557969.1	ph0011	348,217		Asteriusvirus	77.4	16	2
ON568341.1	UE-S1	358,076	8298*	Asteriusvirus	76.8	15	2
OR670491.1	vB_EcoM_JNE01	355,583		Not defined	60.5	20	7
ON286972.1	A4	355,528		Not defined	58.5	14	5
OQ411014.1	EJP2	349,185		Not defined	35.4	18	6
JQ513383.1	vB_KleM-RaK2	345,809		Alcyoneusvirus	30.4	1	1
MT708547.1	Muenster	346,937	20,030	Alcyoneusvirus	30.4	9	1
NC_027399.1	K64-1	346,602		Alcyoneusvirus	30.4	13	1
OP617745.1	KP13-7	357,701		Alcyoneusvirus	29.5	17	1
PP191103.1	PHA46	337,158		Not defined	29.4	7	4
MK773491.1	7t3	348,718		Not defined	29.0	7	4
MT001829.1	SE_PL	348,657		Not defined	29.0	7	4
MK268344.1	Munch	350,103		Not defined	28.9	7	4
OR228459.1	fENko-Kae01	360,105	19,736	Not defined	28.6	7	4
LT960552.1	fHe-Yen9-03	352,596		Eneladusvirus	23.7	4	3
NC_042116.1	fHe-Yen9-04	354,378		Eneladusvirus	23.3	4	3

**Fig. 5:**
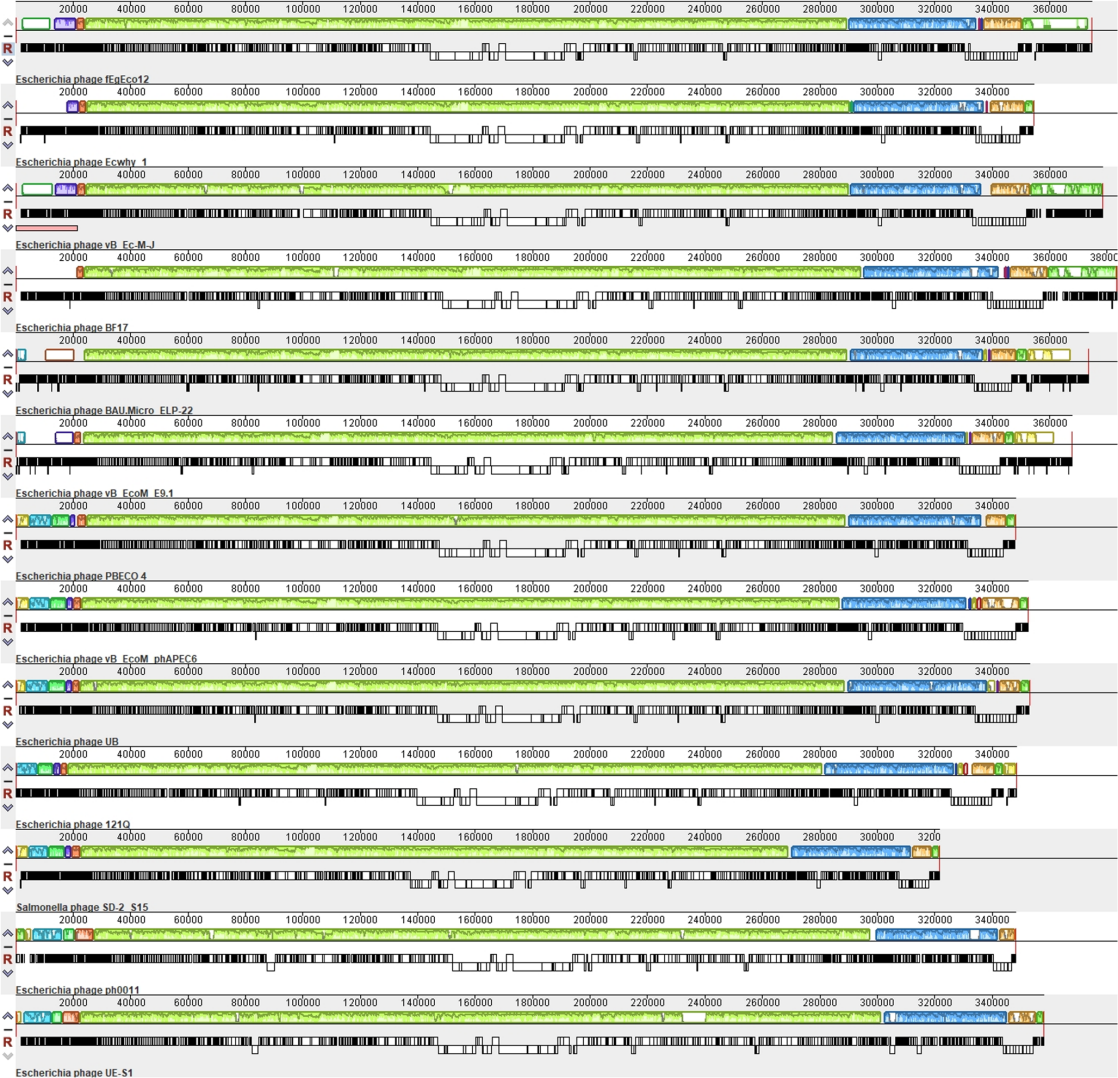
Whole genome alignment with progressive MAUVE of the *Asteriusvirus* phages listed in Table 2. The genomes of phages Ecwhy_1, vB_Ec-M-J, BF17, BAU.Micro_ELP-22, vB_EcoM_E9.1, PBECO4, vB_EcoM_phAPEC6, 121Q, ph0011, SD-2_S15 and UE-S1 were rearranged to match the starting point of phage fEgEco12. The colored collinear blocks indicate homologous regions between the genome sequences while the height of the similarity profile in the collinear blocks indicate average level of conservation in the regions of the genome sequence

*The progressive Mauve alignment (Fig. 5) indicates that this terminal repeat may not be correctly assigned

Among all the jumbo phages, the physical termini of only five phage genomes have been determined (Table 2), and all of them possess ca. 20 kb direct repeats, except phage UE-S1, that was reported to possess an 8.3 kb direct terminal repeat (but see below). It is very likely that the other jumbo phages also possess long ca. 20 kb direct terminal repeats.

To carry out more detailed genomic comparison of these closely related phages, a progressive Mauve alignment of the 14 phages representing distinct *Asteriusvirus* species clusters (Table 2) was carried out (Fig. 5). For the alignment, the genomes of some phages were rearranged to match the starting point of fEgEco12, for which the genome termini were confirmed by PhageTerm and restriction digestion analyses (Fig. 2). The alignment showed a strong synteny among the genomes and highlighted the similarities and differences and revealed that the phages have a conserved modular structure with collinear blocks throughout their genomes (Fig. 5). However, certain locally collinear blocks with a high number of genes encoding hypothetical and accessory proteins showed major variations amongst them. Interestingly, phage fEgEco12 contains some genes not present in the other phages, which indicates lineage-specific gene acquisition. Of note, the Mauve alignment predicts that the terminal repeat annotated for phage UE-S1 is not correctly assigned. Collectively, these results clearly suggest that the jumbo phage fEgEco12 together with the other *Escherichia* phages are closely related members of genus *Asteriusvirus.*

**Fig. 6:**
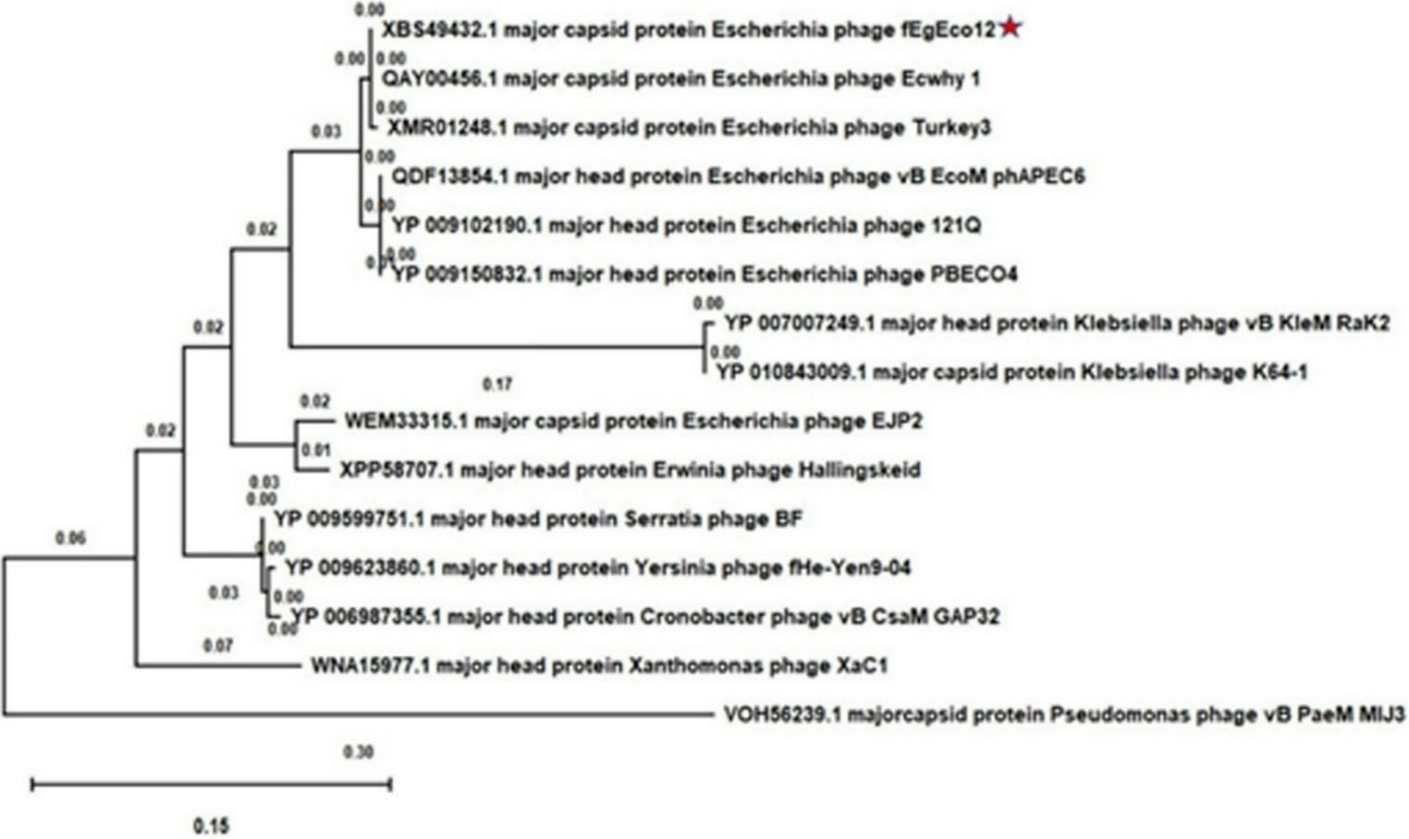
Phylogenetic analysis based on the major capsid protein sequences of representatives of several genera of the *Caudoviricites* class

The relationship of phage fEgEco12 among the *Caudoviricetes* was further confirmed by the phylogenetic analysis performed using the major capsid protein sequences. The results show that phage fEgEco12 belongs to the cluster formed by the *Asteriusvirus* genus (Fig. 6) in full agreement with the results reported above.

**Fig. 7:**
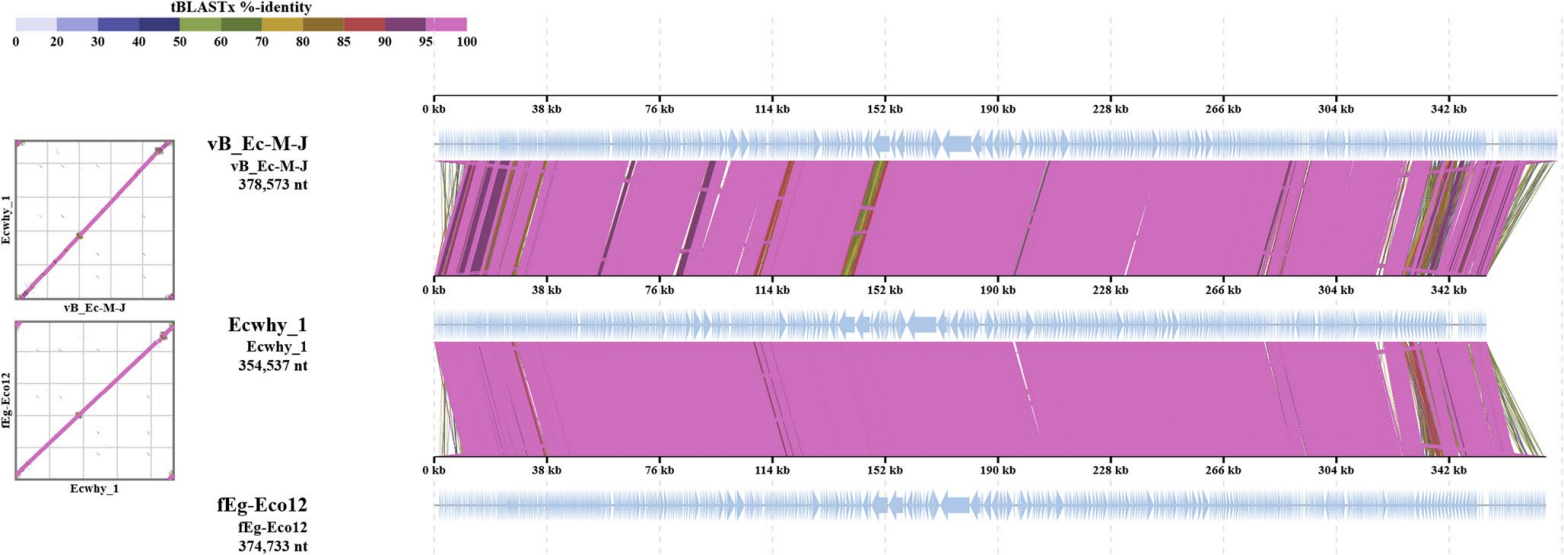
Alignment of phage fEgEco12, Ecwhy_1 and vB_Ec-M-J genomes using the VipTree tool

To pinpoint the genomic differences between the closest related phages, the genomes of phages fEgEco12, Ecwhy_1 and vB_Ec-M-J were aligned using the VipTree tool (Fig. 7). While fEgEco12 and Ecwhy_1 represent the same species and differ from each other only marginally, vB_Ec-M-J represents another phage species and contains several differences. Comparison of the gene products of fEgEco12 and Ecwhy_1 revealed that seven gene products displayed < 50% amino acid identity, whereas four proteins exhibited moderate similarity ranging from 51.0% to 72.6% (Table S3). Remarkably, two of the highly different proteins were annotated as chromosome condensation regulators, suggesting potential functional diversity between the two phages. A total of 59 predicted gene products of fEgEco12 were completely absent in Ecwhy_1 (Table S4). Only three of them had functional annotation. Gp520 and Gp570 are annotated as HNH endonucleases, and Gp519 as RNA polymerase sigma factor (Table S4). In addition, altogether 20 predicted gene products (all annotated as hypothetical proteins) of Ecwhy_1 were completely absent in fEgEco12 (data not shown).

### Phage host range

fEgEco12 infected efficiently only 15 (11%) of the 132 *E. coli* strains, and in addition one *Klebsiella pneumoniae* strain (Table S1). fEgEco12 did not infect any of the other bacterial species tested. fEgEco12 seems to have a relatively narrow host-range which can limit its applicability in phage therapy. However, host range expansion protocols have been developed to overcome the limitations of narrow host-range phages [59, 60].

### Phage stability

**Fig. 8:**
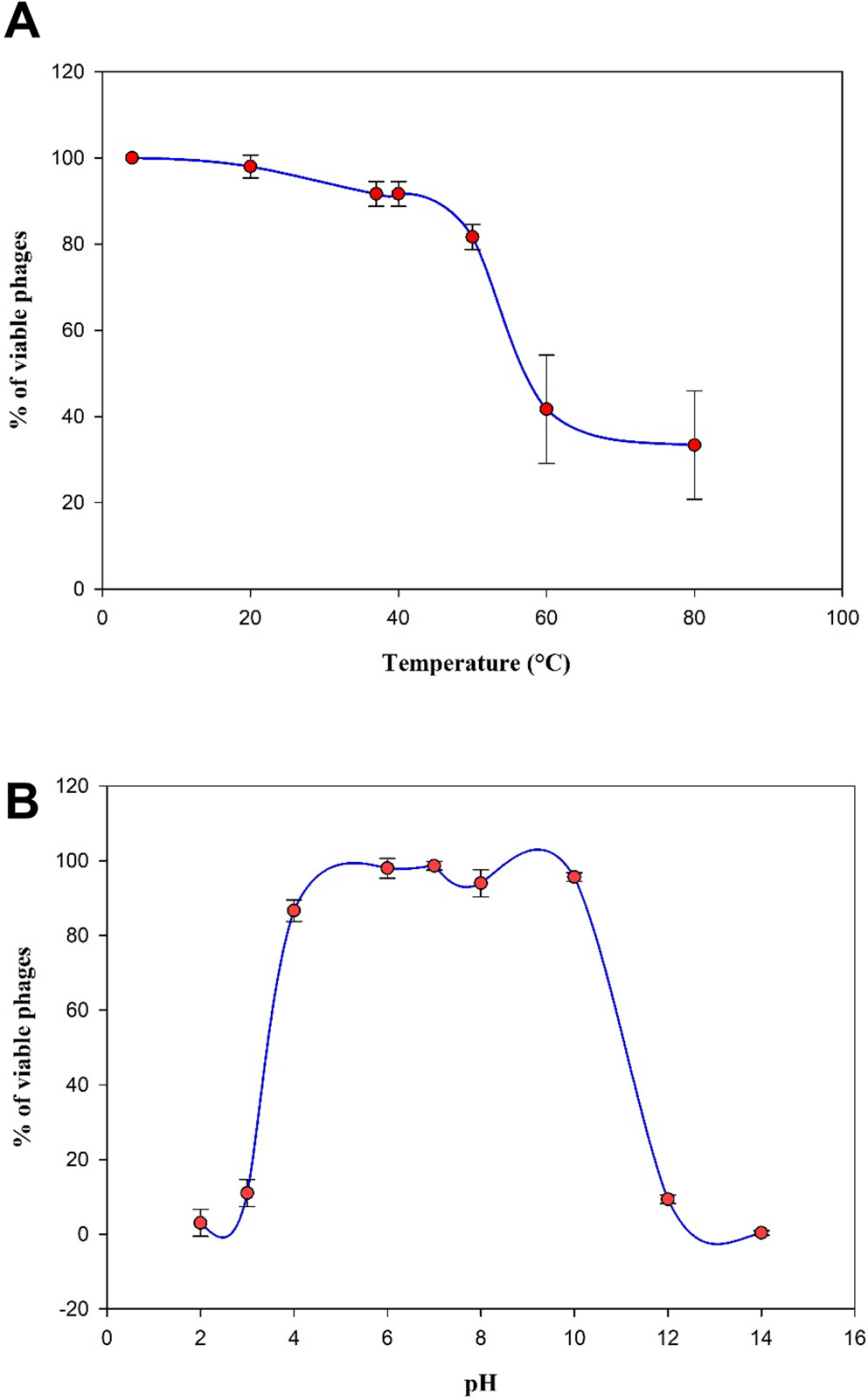
Stability of phage fEgEco12 under different environmental conditions. Temperature (**A**) and pH (**B**). Error bars indicate standard deviation (*n* = 3)

The stability of phage fEgEco12 under various temperature and pH conditions, is demonstrated in Fig. 8. The thermostability results revealed that phage fEgEco12 survives well at temperatures between 4 °C and 50 °C and retains its titer of ~ 10^9^ PFU/ml but does not tolerate higher temperatures. The phage titer dropped below the detection level at 60 and 80 °C when compared to 4 °C (Fig. 8A). Even then, some phages survived the incubation at 80 °C. Importantly, phage fEgEco12 was stable for 5 years when stored at 4 °C. Regarding the pH-tolerance, no significant differences were seen in the phage titers (average ~ 10^9^ PFU/ml) after 1 h incubation at pHs between 4 and 10 (Fig. 8B), while at pH 3 and at pH 12 the titers decreased significantly. No phages survived at pH 14. In conclusion, significant decreases in phage titers were observed at extreme temperatures and pH compared to standard conditions.

Phage adsorption and single-step growth curves.

**Fig. 9:**
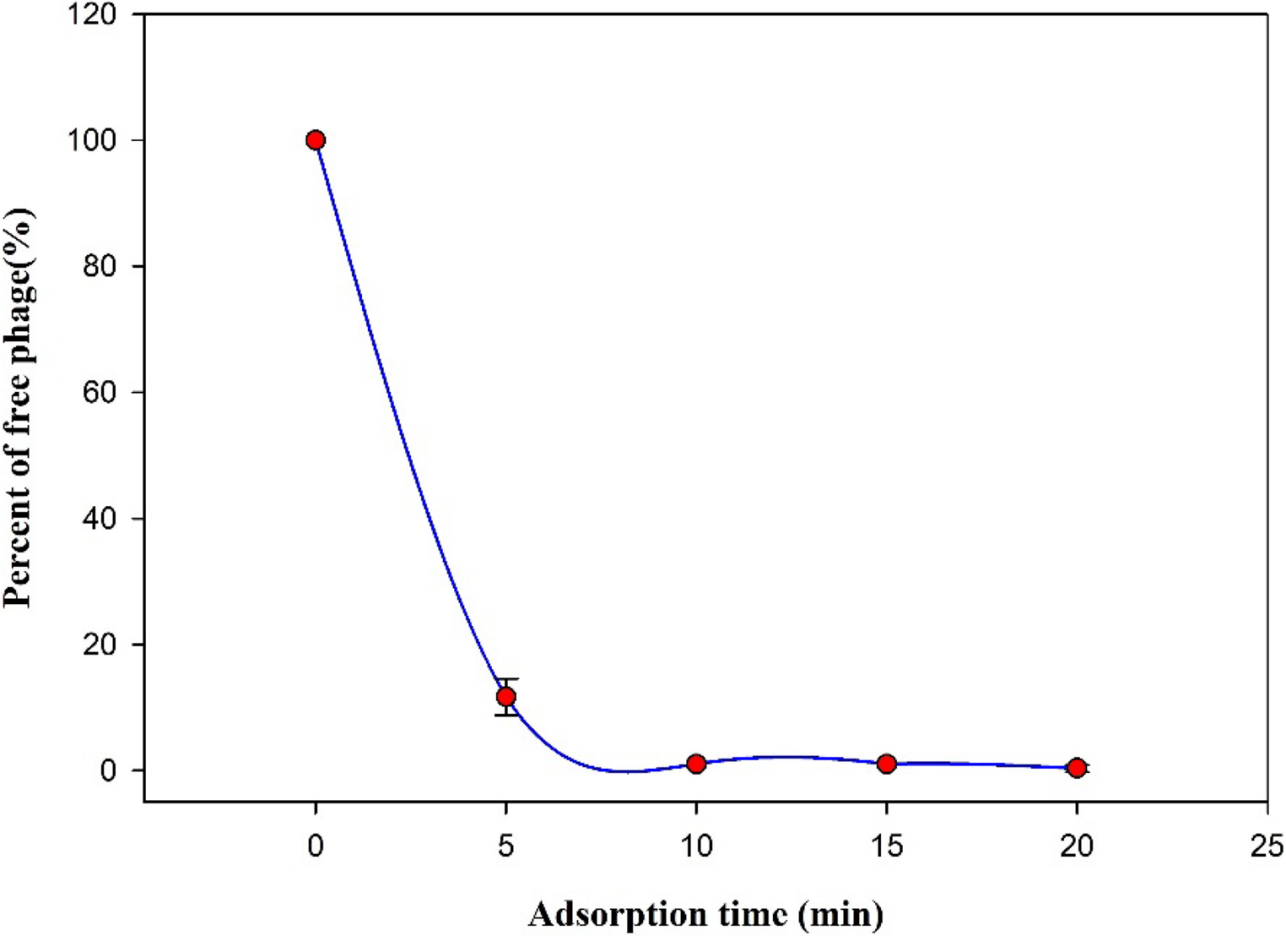
Adsorption curve of the Escherichia phage fEgEco12. Error bars indicate standard deviation (*n* = 3)

**Fig. 10:**
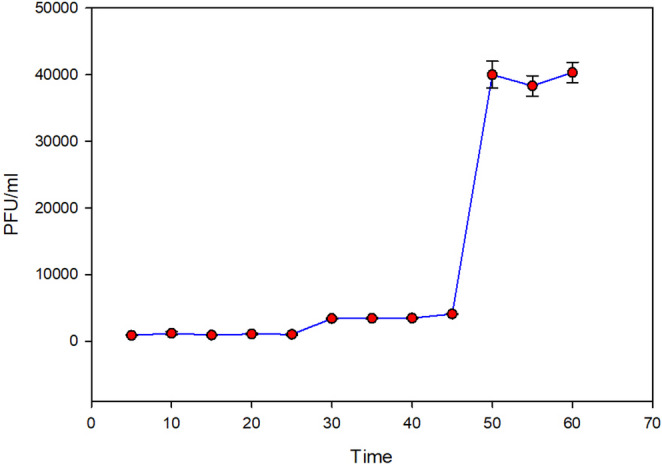
One step growth curve of Escherichia phage fEgEco12. The average phage titer for time points 5–25 min was 1042.5 PFU/mL and for time points 50–60 min, 39.6 × 10^3^ PFU/mL. These values were used to calculate the burst size. Error bars indicate standard deviation (*n* = 3)

Phage adsorption assay showed that approximately 90% of phage fEgEco12 adsorbed to the host bacterial cells in 5 min and 99% within 15 min (Fig. 9). The latent period of phage fEgEco12 was 45 min and the burst size, 38 PFU/infected cell (Fig. 10).

## Conclusions

In conclusion, this study describes the isolation and detailed characterization of the novel *Escherichia* jumbo bacteriophage, fEgEco12, active against avian pathogenic *E. coli* (APEC) strains. Morphological, genomic, and bioinformatic analyses classified fEgEco12 as a strictly lytic jumbo phage belonging to the class *Caudoviricetes* and the genus *Asteriusvirus*. The phage possesses a large double-stranded DNA genome lacking genes associated with lysogeny, virulence, or antimicrobial resistance, supporting its safety for therapeutic applications. fEgEco12 encodes its own replication and lysis machinery, indicating a high degree of autonomy during infection. Comparative genomic analyses revealed conserved modular genome organization and confirmed that fEgEco12 represents a new species within its genus. Despite exhibiting a relatively narrow host range, the phage efficiently infected multiple APEC strains and one *Klebsiella pneumoniae* isolate. fEgEco12 demonstrated rapid adsorption, a short latent period, and strong environmental stability across a wide range of temperatures and pH values. Long-term stability at 4 °C further supports its practical applicability. The phage showed strong in vitro antibacterial activity against MDR-APEC strains. These characteristics highlight the potential of fEgEco12 as a biocontrol agent in poultry production. However, in vivo efficacy and safety must be validated through controlled field trials. Overall, this work contributes valuable insights into the diversity and therapeutic potential of jumbo bacteriophages targeting pathogenic *E. coli*.

## Supplementary Information

Below is the link to the electronic supplementary material.

Supplementary Material 1(PDF 3.53 MB)

## Data Availability

The annotated genomic sequence of fEgEco12 was deposited in NCBI GenBank under the accession number PP777464.1.

## Abbreviations

APEC: avian pathogenic *E. coli*
DEC: diarrheagenic *E. coli*
DLA: double-layer agar
ExPEC: extraintestinal pathogenic *E. coli*
Kb: kilobases
LA: Lysogeny Broth agar
LB: Lysogeny Broth
MDR: multi-drug resistant
PFU/mL: plaque-forming units per milliliter
RT: room temperature
RAST: rapid annotation subsystems technology
SD: standard deviation
SDS: sodium dodecyl sulphate
TEM: Transmission electron microscopy
UPEC: uropathogenic *E. coli*
VIRIDIC: Virus Intergenomic Distance Calculator
