# Supplementary material for: Genomic analysis of jumbo coliphage fEgEco12
Source: Arch Virol. 2026 Apr 12;171(5):160. doi: 10.1007/s00705-026-06623-3 (PMC13070987; doi:10.1007/s00705-026-06623-3)
Supplement: Supplementary file 1 — Supplementary Material 1(PDF 3.53 MB) [file 705_2026_6623_MOESM1_ESM.pdf]

Supplementary Information

Genomic analysis of Jumbo Coliphage fEgEco12

Shimaa Badawy[1,2] and Mikael Skurnik[1*]

[1] Department of Bacteriology and Immunology, Medicum, Human Microbiome Research Program, Faculty of Medicine, University of Helsinki, 00014 UH Helsinki, Finland; shimaa_a_badwy@yahoo.com (S.B.)

[2] Department of Botany and Microbiology, Faculty of Science, Damietta University, 34511 New Damietta, Egypt

* Correspondence: mikael.skurnik@helsinki.fi; Tel.: +358-50-3360981

Supplementary figures

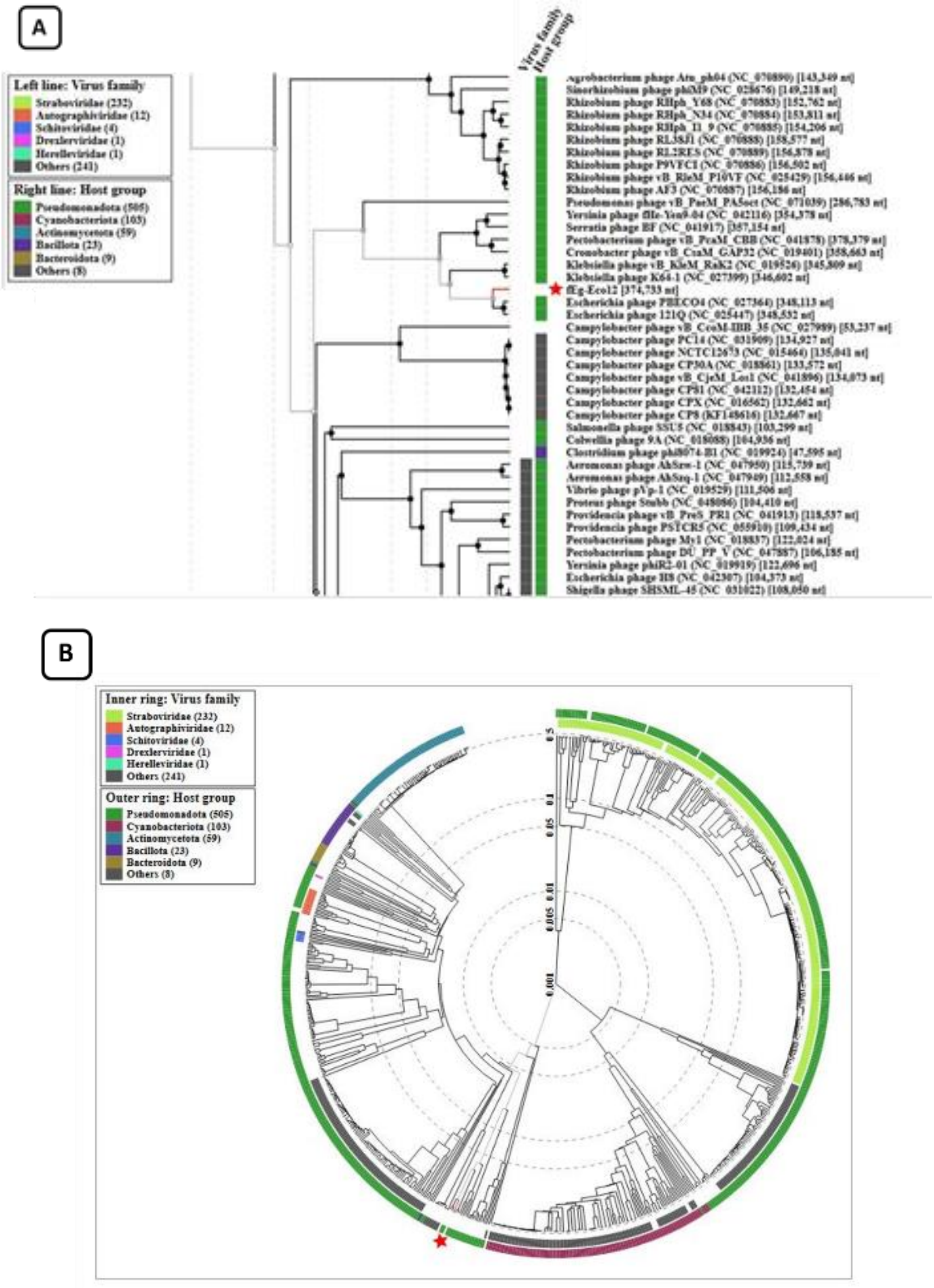

Figure S1. Genome comparison and phylogenetic proteomic tree analysis of fEgEco12 with related phages. **A.** A linear proteomic tree of the most similar 41 sequences to fEgEco12 according to their SG scores, designed by VipTree. **B.** Circular proteomic phylogenetic tree of fEgEco12 with the closest phage genomes according to BLASTn top matches, designed by VipTree. The inner and outer colors indicate viral families and host groups, respectively. The red asterisks indicate the phage's location in the trees

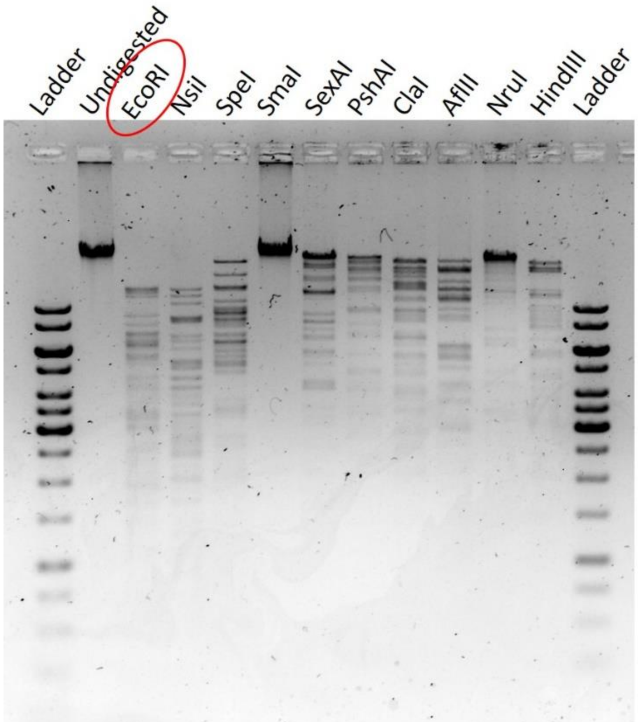

EcoRI

NEB Cutter prediction

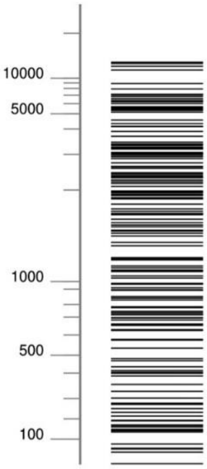

#	Ends	Coordinates	Length (bp)
1	EcoRI - EcoRI	352846-366356	13511
2	EcoRI - EcoRI	14987-28203	13217
3	(LeftEnd) - EcoRI	1-12616	12616
4	EcoRI - EcoRI	177168-188964	11797
5	EcoRI - EcoRI	93506-102388	8883
6	EcoRI - EcoRI	313538-321438	7901
7	EcoRI - EcoRI	328416-335533	7118
8	EcoRI - EcoRI	62197-69299	7103
9	EcoRI - EcoRI	75468-82508	7041
10	EcoRI - EcoRI	143327-150169	6843
11	EcoRI - EcoRI	231508-238092	6585
12	EcoRI - EcoRI	211115-217659	6545
13	EcoRI - EcoRI	336723-343051	6329
14	EcoRI - EcoRI	150170-156485	6316
15	EcoRI - EcoRI	343052-349282	6231
16	EcoRI - EcoRI	285121-291325	6205
17	EcoRI - (RightEnd)	368727-374733	6007

Figure S2. fEgEco12 restriction sites *in silico* vs experimental digestion with EcoRI. The NEB Cutter prediction of the sizes of the longest fragments are listed in the table, and the predicted migration pattern in agarose gel is indicated in the middle.

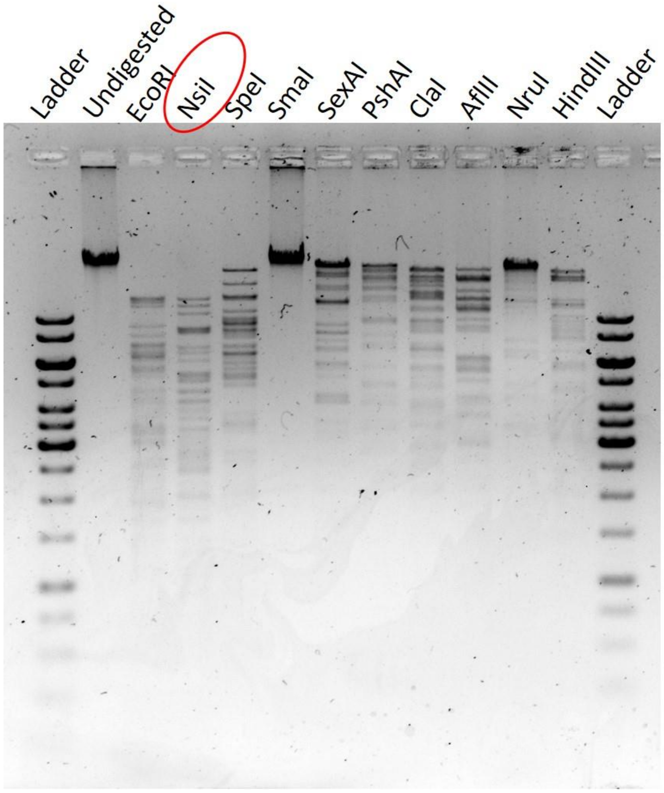

NsiI

NEB Cutter prediction

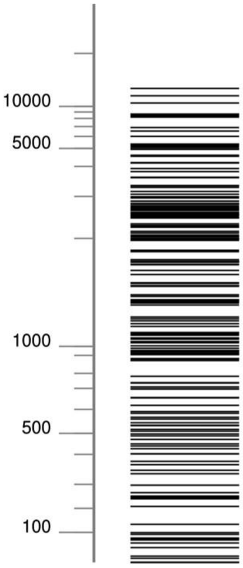

#	Ends	Coordinates	Length (bp)
1	NsiI - NsiI	172347-185711	13365
2	NsiI - NsiI	230365-242352	11988
3	NsiI - NsiI	145083-155720	10638
4	NsiI - NsiI	7776-16423	8648
5	NsiI - NsiI	361516-370163	8648
6	NsiI - NsiI	93885-102311	8427
7	NsiI - NsiI	321366-329700	8335
8	NsiI - NsiI	156627-164808	8182
9	NsiI - NsiI	68450-75342	6893
10	NsiI - NsiI	210754-217270	6517
11	NsiI - NsiI	164809-170813	6005
12	NsiI - NsiI	218370-223704	5335
13	NsiI - NsiI	203884-209192	5309
14	NsiI - NsiI	264509-269720	5212
15	NsiI - NsiI	291560-296726	5167
16	NsiI - NsiI	136235-141304	5070
17	NsiI - NsiI	120285-125242	4958

Figure S3. fEgEco12 restriction sites *in silico* vs experimental digestion with NsiI. The NEB Cutter prediction of the sizes of the longest fragments are listed in the table, and the predicted migration pattern in agarose gel is indicated in the middle.

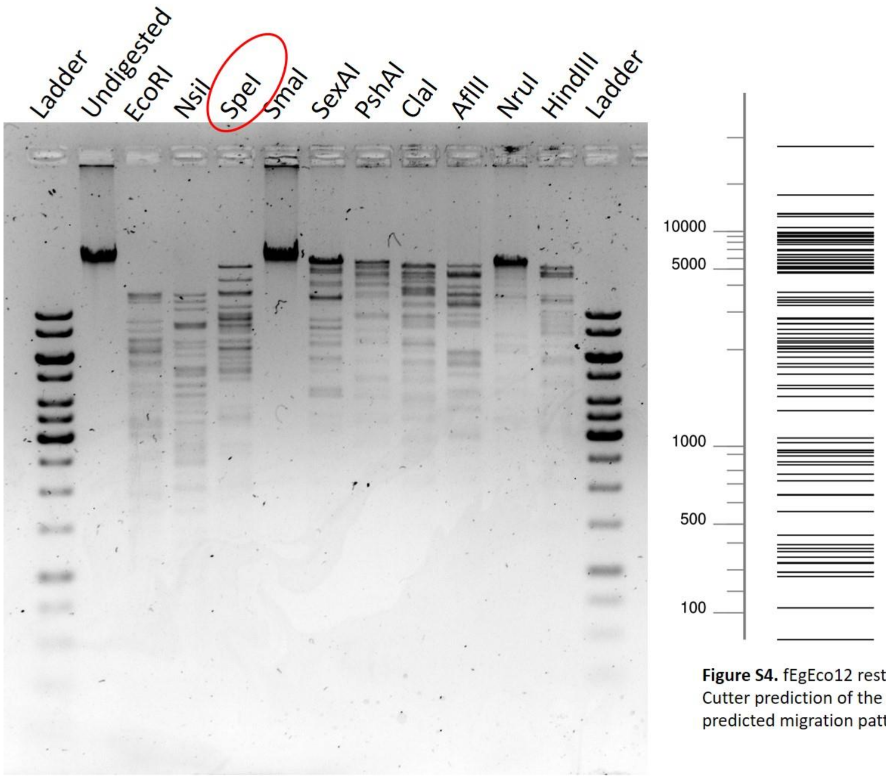

SpeI

NEB Cutter prediction

#	Ends	Coordinates	Length (bp)
1	SpeI - SpeI	11421-39514	28094
2	SpeI - SpeI	295571-313232	17662
3	SpeI - SpeI	39515-53262	13748
4	SpeI - SpeI	132077-145701	13625
5	SpeI - SpeI	69221-82337	13117
6	SpeI - SpeI	347939-358760	10822
7	SpeI - SpeI	254756-264575	9820
8	SpeI - (RightEnd)	365161-374733	9573
9	SpeI - SpeI	206804-216013	9210
10	SpeI - SpeI	145974-155150	9177
11	SpeI - SpeI	91897-101048	9152
12	SpeI - SpeI	316672-325572	8901
13	SpeI - SpeI	102793-111232	8440
14	SpeI - SpeI	192197-200613	8417
15	SpeI - SpeI	222904-231164	8261
16	SpeI - SpeI	286521-294496	7976
17	SpeI - SpeI	277274-284942	7669

Figure S4. fEgEco12 restriction sites *in silico* vs experimental digestion with SpeI. The NEB Cutter prediction of the sizes of the longest fragments are listed in the table, and the predicted migration pattern in agarose gel is indicated in the middle.

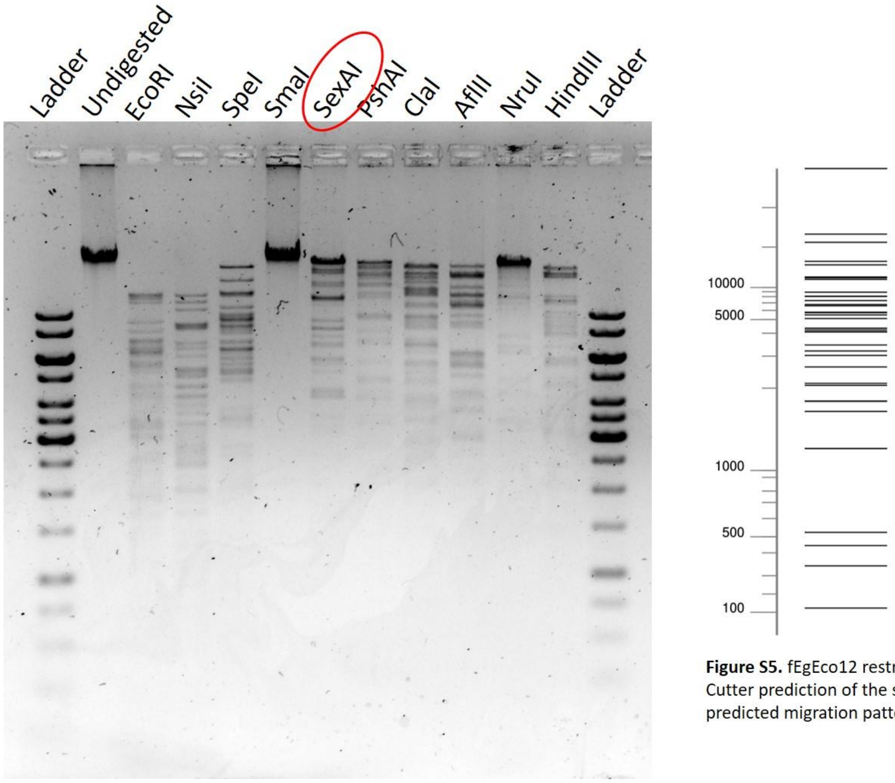

SexAI

NEB Cutter prediction

#	Ends	Coordinates	Length (bp)
1	SexAI - SexAI	248588-295999	47412
2	SexAI - SexAI	12954-57882	44929
3	SexAI - SexAI	120607-161549	40943
4	SexAI - SexAI	176004-199250	23247
5	SexAI - SexAI	214961-236155	21195
6	SexAI - SexAI	84383-100834	16452
7	SexAI - SexAI	68821-84382	15562
8	SexAI - SexAI	104169-116736	12568
9	SexAI - SexAI	163605-176003	12399
10	SexAI - SexAI	343155-355517	12363
11	SexAI - SexAI	327201-339165	11965
12	SexAI - SexAI	296000-304898	8899
13	SexAI - SexAI	316101-324170	8070
14	SexAI - (RightEnd)	366694-374733	8040
15	SexAI - SexAI	236156-243495	7340
16	SexAI - SexAI	62053-68820	6768
17	SexAI - SexAI	205816-212401	6586

Figure S5. fEgEco12 restriction sites *in silico* vs experimental digestion with SexAI. The NEB Cutter prediction of the sizes of the longest fragments are listed in the table, and the predicted migration pattern in agarose gel is indicated in the middle.

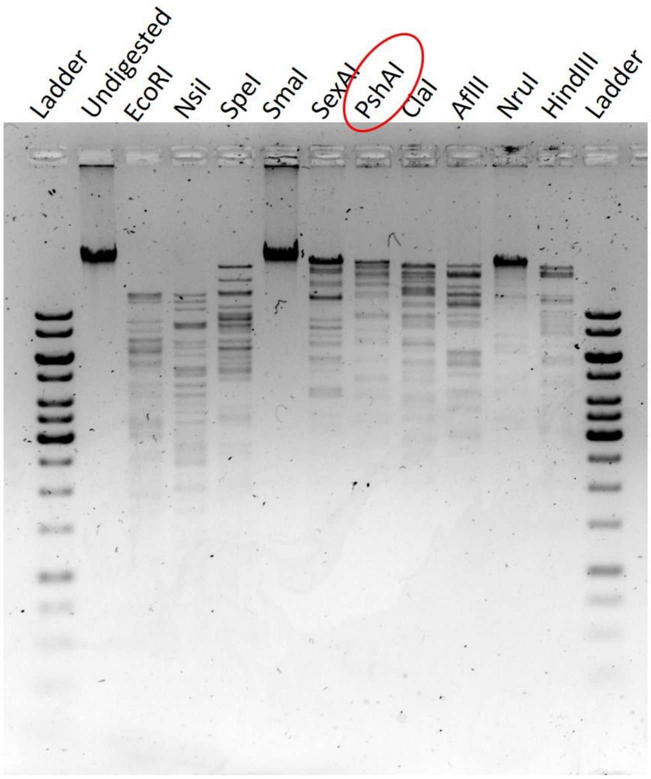

PshAI

NEB Cutter prediction

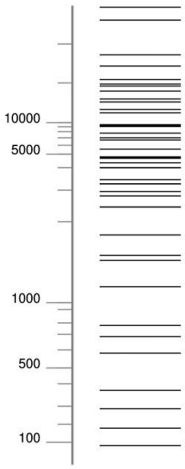

#	Ends	Coordinates	Length (bp)
1	PshAI - PshAI	262733-302312	39580
2	PshAI - PshAI	327033-363244	36212
3	PshAI - PshAI	43828-71037	27210
4	PshAI - PshAI	19524-43827	24304
5	PshAI - PshAI	97544-118396	20853
6	PshAI - PshAI	235320-255003	19684
7	PshAI - PshAI	118397-137572	19176
8	PshAI - PshAI	144244-162161	17918
9	PshAI - PshAI	71038-86959	15922
10	PshAI - PshAI	175458-190609	15152
11	PshAI - PshAI	162162-175457	13296
12	PshAI - PshAI	202192-214638	12447
13	(LeftEnd) - PshAI	1-9504	9504
14	PshAI - PshAI	226060-235319	9260
15	PshAI - PshAI	216991-226059	9069
16	PshAI - PshAI	255004-262732	7729
17	PshAI - PshAI	89400-96395	6996

Figure S6. fEgEco12 restriction sites *in silico* vs experimental digestion with PshAI. The NEB Cutter prediction of the sizes of the longest fragments are listed in the table, and the predicted migration pattern in agarose gel is indicated in the middle.

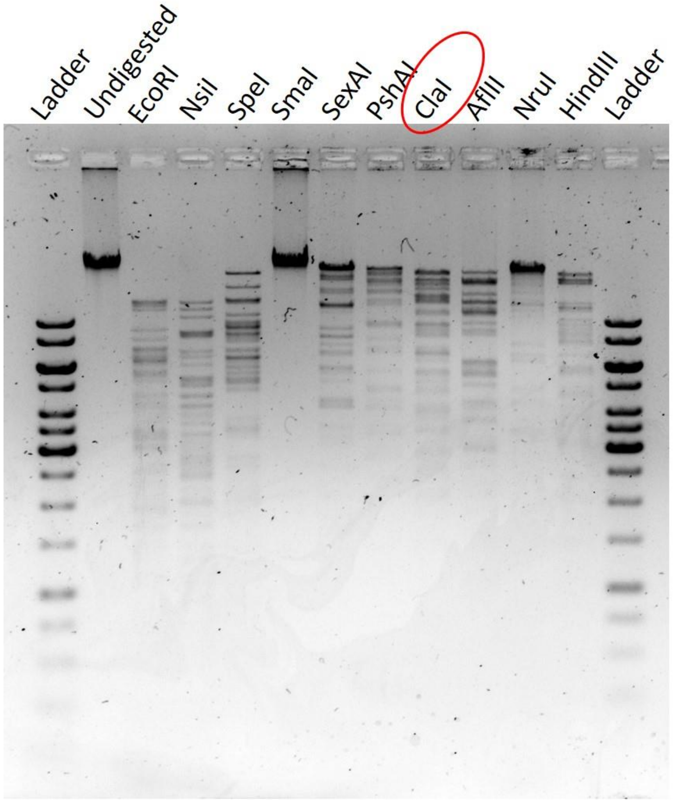

ClaI

NEB Cutter prediction

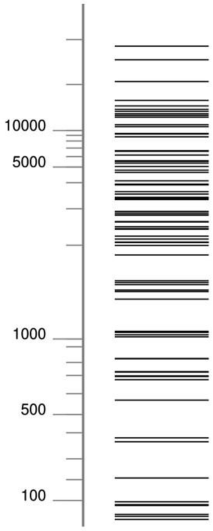

#	Ends	Coordinates	Length (bp)
1	ClaI - ClaI	71029-99537	28509
2	ClaI - ClaI	323617-349090	25474
3	ClaI - ClaI	234970-255617	20648
4	ClaI - ClaI	106465-122979	16515
5	ClaI - ClaI	285981-301293	15313
6	ClaI - ClaI	54964-69539	14576
7	ClaI - ClaI	195270-209411	14142
8	ClaI - ClaI	351379-364983	13605
9	ClaI - ClaI	170029-183285	13257
10	ClaI - ClaI	35477-48345	12869
11	(LeftEnd) - ClaI	1-11243	11243
12	ClaI - ClaI	148256-159085	10830
13	ClaI - ClaI	224767-234144	9378
14	ClaI - ClaI	215496-224766	9271
15	ClaI - ClaI	272890-281620	8731
16	ClaI - ClaI	25910-32577	6668
17	ClaI - ClaI	11244-17881	6638

Figure S7. fEgEco12 restriction sites *in silico* vs experimental digestion with ClaI. The NEB Cutter prediction of the sizes of the longest fragments are listed in the table, and the predicted migration pattern in agarose gel is indicated in the middle.

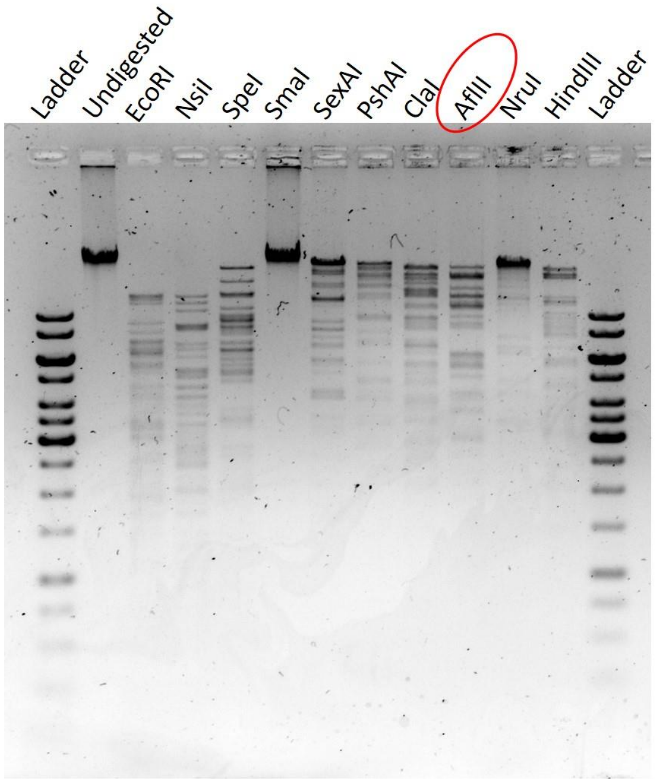

AflII

NEB Cutter prediction

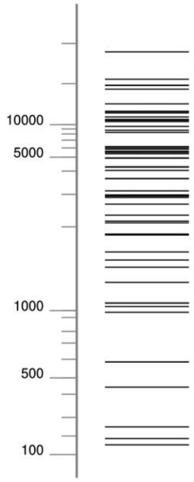

#	Ends	Coordinates	Length (bp)
1	AflII - AflII	202533-230420	27888
2	AflII - AflII	230421-251494	21074
3	AflII - AflII	135258-154863	19606
4	AflII - AflII	115709-135257	19549
5	AflII - AflII	84108-102725	18618
6	AflII - AflII	322531-337572	15042
7	AflII - AflII	267440-280659	13220
8	AflII - AflII	70983-84107	13125
9	AflII - AflII	167710-180521	12812
10	AflII - AflII	184742-196605	11864
11	AflII - AflII	337573-348847	11275
12	AflII - AflII	59769-70982	11214
13	AflII - AflII	285565-296603	11039
14	AflII - AflII	154864-165591	10728
15	AflII - AflII	301731-311306	9576
16	AflII - AflII	15141-23855	8715
17	AflII - AflII	106972-115268	8297

Figure S8. fEgEco12 restriction sites *in silico* vs experimental digestion with AflII. The NEB Cutter prediction of the sizes of the longest fragments are listed in the table, and the predicted migration pattern in agarose gel is indicated in the middle.

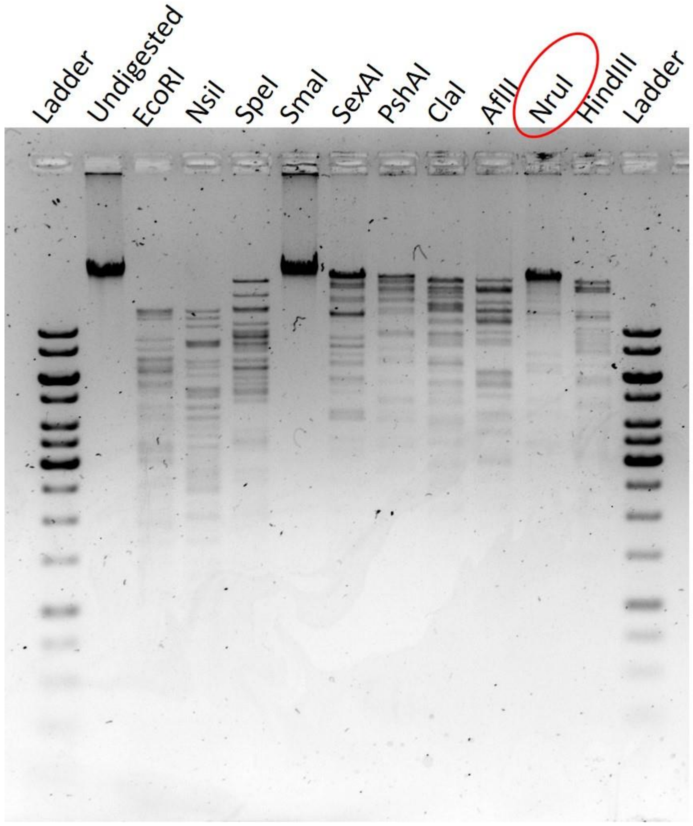

NruI

NEB Cutter prediction

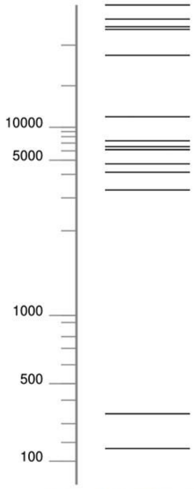

#	Ends	Coordinates	Length (bp)
1	NruI - NruI	245314-311241	65928
2	NruI - NruI	176739-237977	61239
3	NruI - NruI	16315-76475	60161
4	NruI - NruI	323760-360251	36492
5	NruI - NruI	142150-176738	34589
6	NruI - NruI	108226-142149	33924
7	NruI - NruI	80590-108060	27471
8	NruI - NruI	311242-323759	12518
9	NruI - NruI	237978-245313	7336
10	(LeftEnd) - NruI	1-6511	6511
11	NruI - NruI	6512-12656	6145
12	NruI - NruI	360252-366396	6145
13	NruI - (RightEnd)	370055-374733	4679
14	NruI - NruI	76476-80589	4114
15	NruI - NruI	12998-16314	3317
16	NruI - NruI	366738-370054	3317
17	NruI - NruI	12657-12997	341

Figure S9. fEgEco12 restriction sites *in silico* vs experimental digestion with NruI. The NEB Cutter prediction of the sizes of the longest fragments are listed in the table, and the predicted migration pattern in agarose gel is indicated in the middle.

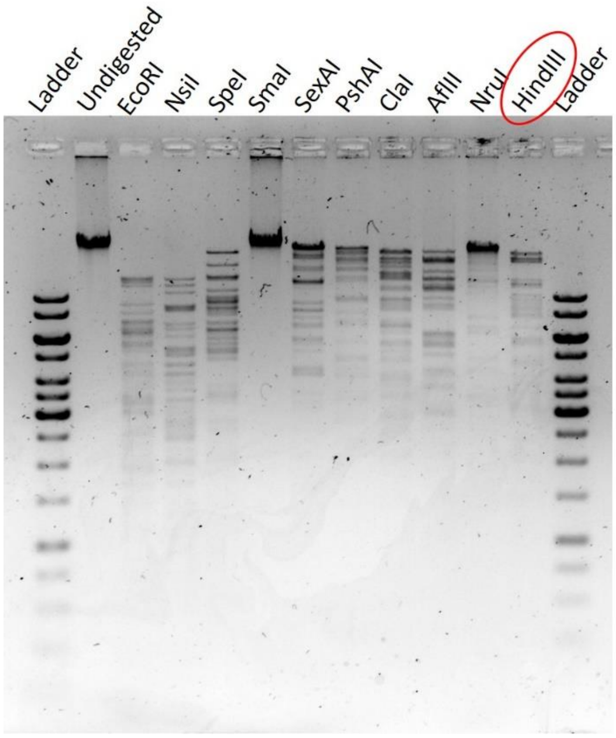

HindIII

NEB Cutter prediction

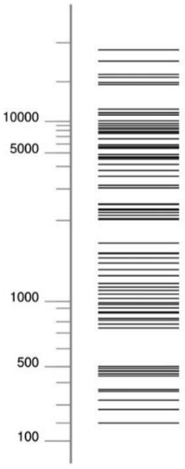

#	Ends	Coordinates	Length (bp)
1	HindIII - HindIII	324210-352380	28171
2	HindIII - HindIII	137999-163279	25281
3	HindIII - HindIII	88560-110386	21827
4	HindIII - HindIII	352381-373505	21125
5	(LeftEnd) - HindIII	1-19765	19765
6	HindIII - HindIII	69263-88559	19297
7	HindIII - HindIII	165004-178051	13048
8	HindIII - HindIII	292408-304599	12192
9	HindIII - HindIII	263858-275962	12105
10	HindIII - HindIII	227733-239338	11606
11	HindIII - HindIII	213401-223568	10168
12	HindIII - HindIII	59766-69262	9497
13	HindIII - HindIII	204421-213400	8980
14	HindIII - HindIII	28469-37001	8533
15	HindIII - HindIII	19766-27998	8233
16	HindIII - HindIII	43875-51715	7841
17	HindIII - HindIII	241322-249012	7691

Figure S10. fEgEco12 restriction sites *in silico* vs experimental digestion with HindIII. The NEB Cutter prediction of the sizes of the longest fragments are listed in the table, and the predicted migration pattern in agarose gel is indicated in the middle.

Supplementary Tables

Table S1. Bacterial strains used in the work[a]. The strains were assayed for phage sensitivity by the Bioscreen C analyser (indicated by + or -) or by the spot test (indicated as ST+ or ST-).

Species	Storage Number	Phage sensitivity	Resistance Phenotype and Origin	Source
Acinetobacter baumannii	5542	ST -	MDR, human	Huslab
	5707	ST -	MDR, hip surgical wound	Huslab
Escherichia coli	5509	ST -	Blood	Huslab
	5510	ST+	Blood	Huslab
	5512	ST -	Blood	Huslab
	5517	ST+	Blood	Huslab
	5519	ST -	Blood	Huslab
	5520	ST -	Blood	Huslab
	5521	ST+	Blood	Huslab
	5522	ST+	Blood	Huslab
	5626	ST+	Urine	Huslab
	5627	ST -	Urine	Huslab
	5629	ST -	Abdominal punction fluid	Huslab
	5632	ST -	Urine	Huslab
	5633	ST+	Rectum mucus	Huslab
	5634	ST -	Rectum mucus	Huslab
	5636	ST+	Urine	Huslab
	5639	ST+	Urine	Huslab
	5759	-	ESBL, stool	Huslab
	5760	+	ESBL, stool	Huslab
	5761	-	ESBL, blood	Huslab
	5762	-	ESBL, stool	Huslab
	5763	-	ESBL, urine	Huslab
	5764	-	ESBL, urine	Huslab
	5765	+	ESBL, urine	Huslab
	5766	-	Abscess in gluteal cleft	Huslab
	5767	-	Foot surgical wound	Huslab
	5768	-	ESBL, rectum slime	Huslab
	5769	+	ESBL, rectum slime	Huslab
	5770	-	ESBL, Human excretion	Huslab
	6040	-	ESBL, Human stool	Huslab
	6041	-	ESBL, Human stool	Huslab
	6042	-	ESBL, Human stool	Huslab
	6043	-	ESBL, Human stool	Huslab
	6044	-	ESBL, Human stool	Huslab
	6045	-	ESBL, Human stool	Huslab
	6046	-	ESBL, Human stool	Huslab
	6047	-	ESBL, Human stool	Huslab
	6048	-	ESBL, Human stool	Huslab
	6049	-	ESBL, Human stool	Huslab
	6050	-	ESBL, Human stool	Huslab
	6051	-	ESBL, Human stool	Huslab
	6052	-	ESBL, Human stool	Huslab
	6053	-	ESBL, Human stool	Huslab
	6054	-	ESBL, Human stool	Huslab
	6055	-	ESBL, Human stool	Huslab

	6056	-	ESBL, Human stool	Huslab
	6057	-	ESBL, Human stool	Huslab
	6058	-	ESBL, Human stool	Huslab
	6059	-	ESBL, Human stool	Huslab
	6060	-	ESBL, Human stool	Huslab
	6061	-	ESBL, Human stool	Huslab
	6062	-	ESBL, Human stool	Huslab
	6063	-	ESBL, Human stool	Huslab
	6064	-	ESBL, Human stool	Huslab
	6065	-	ESBL, Human stool	Huslab
	6066	-	ESBL, Human stool	Huslab
	6067	-	ESBL, Human stool	Huslab
	6068	-	ESBL, Human stool	Huslab
	6070	-	ESBL, Human stool	Huslab
	6071	-	ESBL, Human stool	Huslab
	6072	-	ESBL, Human stool	Huslab
	6073	-	ESBL, Human stool	Huslab
	6074	-	ESBL, Human stool	Huslab
	6075	-	ESBL, Human stool	Huslab
	6076	-	ESBL, Human stool	Huslab
	6077	-	ESBL, Human stool	Huslab
	6078	-	ESBL, Human stool	Huslab
	6079	-	ESBL, Human stool	Huslab
	6080	-	ESBL, Human stool	Huslab
	6081	-	ESBL, Human stool	Huslab
	6082	-	ESBL, Human stool	Huslab
	6083	-	ESBL, Human stool	Huslab
	6084	-	ESBL, Human stool	Huslab
	6085	-	ESBL, Human stool	Huslab
	6086	-	ESBL, Human stool	Huslab
	6087	-	ESBL, Human stool	Huslab
	6088	-	ESBL, Human stool	Huslab
	6089	-	ESBL, Human stool	Huslab
	6090	-	ESBL, Human stool	Huslab
	6091	-	ESBL, Human stool	Huslab
	6092	-	ESBL, Human stool	Huslab
	6093	-	ESBL, Human stool	Huslab
	6094	-	ESBL, Human stool	Huslab
	6095	-	ESBL, Human stool	Huslab
	6096	-	ESBL, Human stool	Huslab
	6097	-	ESBL, Human stool	Huslab
	6098	-	ESBL, Human stool	Huslab
	6099	-	ESBL, Human stool	Huslab
	6100	-	ESBL, Human stool	Huslab
	6101	-	ESBL, Human stool	Huslab
	6102	-	ESBL, Human stool	Huslab
	6103	ST -	ESBL, Human stool	Huslab
	6104	ST -	ESBL, Human stool	Huslab
	6105	ST -	ESBL, Human stool	Huslab
	6106	ST -	ESBL, Human stool	Huslab
	6107	ST -	ESBL, Human stool	Huslab
	6108	ST -	ESBL, Human stool	Huslab
	6109	ST -	ESBL, Human stool	Huslab
	6110	ST -	ESBL, Human stool	Huslab
	6111	ST -	ESBL, Human stool	Huslab
	6112	ST -	ESBL, Human stool	Huslab
	6113	ST -	ESBL, Human stool	Huslab

	6114	ST -	ESBL, Human stool	Huslab
	6115	**ST+**	ESBL, Human stool	Huslab
	6116	ST -	ESBL, Human stool	Huslab
	6117	**ST+**	ESBL, Human stool	Huslab
	6118	**ST+**	ESBL, Human stool	Huslab
	6119	ST -	ESBL, Human stool	Huslab
	6120	ST -	ESBL, Human stool	Huslab
	6121	ST -	ESBL, Human stool	Huslab
	6122	ST -	ESBL, Human stool	Huslab
	6123	ST -	ESBL, Human stool	Huslab
	6124	ST -	ESBL, Human stool	Huslab
	6125	ST -	ESBL, Human stool	Huslab
	6126	ST -	ESBL, Human stool	Huslab
	6127	ST -	ESBL, Human stool	Huslab
	6128	ST -	ESBL, Human stool	Huslab
	6129	ST -	ESBL, Human stool	Huslab
	6130	**ST+**	ESBL, Human stool	Huslab
	6131	ST -	ESBL, Human stool	Huslab
	6132	ST -	ESBL, Human stool	Huslab
	6133	ST -	ESBL, Human stool	Huslab
	6134	ST -	ESBL, Human stool	Huslab
	6135	**ST+**	ESBL, Human stool	Huslab
	6136	ST -	ESBL, Human stool	Huslab
	6137	ST -	ESBL, Human stool	Huslab
	6138	ST -	ESBL, Human stool	Huslab
	6578	ST -	APEC 2248, Chicken peritoneum	DSMZ
	6581	ST -	APEC 9062, Dove	DSMZ
	6741	ST -	ESBL, Urine	Huslab
	6742	ST -	ESBL, Blood	Huslab
	6882	ST -	UPEC, Human acute cystitis	Huslab
	6883	ST -	EPEC, Infant diarrhea	Huslab
Pseudomonas aeruginosa	5538	ST -	MDR, urine	Huslab
	5539	ST -	MDR, urine	Huslab
Staphylococcus aureus	5696	ST -	MRSA, wound pus	Huslab
	5697	ST -	MRSA, vagina	Huslab
	5698	ST -	MRSA, pharynx	Huslab
	5699	ST -	MRSA, pharynx	Huslab
	5700	ST -	MRSA, pharynx	Huslab
	5701	ST -	MRSA, blood	Huslab
	5702	ST -	MRSA, pharynx	Huslab
	5703	ST -	MRSA, pharynx	Huslab
	5704	ST -	MRSA, pharynx	Huslab
	5705	ST -	MRSA, abscess in gluteal cleft	Huslab
Klebsiella pneumoniae	5772	**ST+**	Urine	Huslab

[a] **APEC**: Avian pathogenic *E. coli*; **DSMZ**: (Deutsche Sammlung von Mikroorganismen und Zellkulturen GmbH) German Collection of Microorganisms and Cell Culture GmbH; **ESBL**: Extended Spectrum Beta-Lactamase; **HUSLAB**: Hospital District of Helsinki and Uusimaa Laboratories; **MDR:** Multidrug resistant;**UPEC**: Uropathogenic strains of *E. Coli*; **MRSA:** Methicillin-resistant *Staphylococcus aureus*

Supplementary Table S2. The predicted gene products and functions of *Escherichia* phage fEgEco12.

Gp*	Genomic location (strand)	MW (Da)	AA (*n*)	Predicted function	Best hit	BLASTP e-value(% identity)	Query Coverage %	HHPRED e-value (probability %)	Organism
Gp1	1689:2108 (f)	16045	139	hypothetical protein Ecwhy1_506	QAY00779.1	1e-99 (100)	100		Escherichia phage Ecwhy_1
Gp2	2202:2441 (f)	9006	79	hypothetical protein ACQ29_gp277	YP_009150591.1	8e-51 (98.73)	100		Escherichia phage PBECO4
Gp3	2528:2794 (f)	10170	88	hypothetical protein Ecwhy1_504	QAY00777.1	1e-54(98.86%)	100		Escherichia phage Ecwhy_1
Gp4	2876:3112 (f)	9192	78	hypothetical protein ESCO32_00155	UPW38588.1	1e-46 (94.81)	98		Escherichia phage vB_EcoM_ESCO32
Gp5	3278:3388 (f)	4272	36	hypothetical protein A4_267	USL83343.1	7e-05 (100)	61		Escherichia phage A4
Gp6	3540:4151 (f)	23518	203	hypothetical protein PSLUR01_00172	SCA80149.1	3e-144 (99.51)	100		Escherichia phage vB_Eco_slurp01
Gp7	4244:4408 (f)	5983	54	hypothetical protein JR323_gp138	YP_009985993.1	5e-12 (62.75)	94		Escherichia phage nepoznato
Gp8	4467:4718 (f)	9102	83	hypothetical protein EO157G_2500	BBM61839.1	2e-54 (100)	100		Escherichia phage SP27
Gp9	4963:5199 (f)	8735	78	hypothetical protein P3ECOSTME9A_CDS0016	XDC03237.1	1e-50 (98.72)	100		Escherichia phage vB_EcoM_E9.1
Gp10	5284:5583 (f)	10816	99	Protein spackle; APOBEC, deaminase, hydrolase, viral protein	6X6O_A			3.9 (69.84)	Escherichia virus T4
Gp11	5605:5835 (f)	8065	76	hypothetical protein Ecwhy1_494	QAY00768.1	2e-44 (100)	100		Escherichia phage Ecwhy_1
Gp12	6069:6551 (f)	17469	160	hypothetical protein Ecwhy1_493	QAY00767.1	5e-107 (98.75)	100		Escherichia phage Ecwhy_1
Gp13	6827:6979 (f)	5627	50	hypothetical protein CPT_Munch_012	AZU97956.1	2e-07 (48.00)	100		Salmonella phage Munch
Gp14	7050:7277 (f)	8201	75	hypothetical protein ACQ29_gp266	YP_009150580.1	2e-45 (98.67)	100		Escherichia phage PBECO4
Gp15	7363:7506 (f)	5314	47	hypothetical protein vBEcoMphAPEC6_gp526c	QDF14149.1	7e-25 (97.87)	100		Escherichia phage vB_EcoM_phAPEC6
Gp16	7596:7919 (f)	11951	107	hypothetical protein	WIL00533.1	2e-66 (93.46)	100		Escherichia phage vB_EcoM_CRJP21
Gp17	8000:8269 (f)	10230	89	hypothetical protein EO157G_2580	BBM61847.1	2e-59 (98.88)	100		Escherichia phage SP27
Gp18	8321:8593 (f)	10161	90	hypothetical protein Ecwhy1_488	QAY00762.1	2e-58 (100)	100		Escherichia phage Ecwhy_1
Gp19	8599:8808 (f)	7708	69	hypothetical protein	WIL00536.1	7e-40 (97.10)	100		Escherichia phage vB_EcoM_CRJP21
Gp20	8878:9024 (f)	5368	48	hypothetical protein G17_00034	QBO61530.1	6e-23 (95.83)	100		Escherichia phage vB_EcoM_G17

Gp21	9106:9258 (f)	5809	50	hypothetical protein A4_286	WP_210765633.1	1e-27 (98)	100		Escherichia phage A4
Gp22	9255:9344 (f)	3169	29	hypothetical protein vBEcoMphAPEC6_gp520c	QDF14143.1	2e-20 (93.10)	100		Escherichia phage vB_EcoM_phAPEC6
Gp23	9345:9488 (f)	5216	47	hypothetical protein EcMJ_023	WGM49266.1	2e-23 (97.87)	100		Escherichia phage vB_Ec-M-J
Gp24	9699:10040 (f)	12593	113	hypothetical protein	WIL00539.1	2e-75 (100)	100		Escherichia phage vB_EcoM_CRJP21
Gp25	10137:10349 (f)	7895	70	hypothetical protein	QXN76009.1	2e-41 (97.14)	100		Escherichia phage BF17
Gp26	10433:10612 (f)	6943	59	hypothetical protein ACQ29_gp256	YP_009150570.1	2e-32 (96.61)	100		Escherichia phage PBECO4
Gp27	10703:10846 (f)	5246	47	Tail fiber protein gp22; crAssphage, bacteriophage, virus, DNA virus, portal, connector, tail,	7QOL_d			1.1(81.13)	Bacteroides phage crAss001
Gp28	10952:11149 (f)	7669	65	hypothetical protein Ecwhy1_481	QAY00755.1	1e-36 (100)	100		Escherichia phage Ecwhy_1
Gp29	11333:11479 (f)	5460	48	hypothetical protein PBI_121Q_293	YP_009101880.1	2e-20 (89.58)	100		Escherichia phage 121Q
Gp30	11479:11736 (f)	9741	85	DksA-like zinc-finger protein	WGM49272.1	1e-49 (89.41)	100		Escherichia phage vB_Ec-M-J
Gp31	11800:12156 (f)	13312	118	GNAT family N-acetyltransferase	YP_009101882.1	3e-77 (94.92)	100		Escherichia phage 121Q
Gp32	12235:12423 (f)	6806	62	hypothetical protein ACQ29_gp252	YP_009150566.1	3e-36 (100)	100		Escherichia phage PBECO4
Gp33	12451:12774 (f)	12168	107	hypothetical protein vBEcoMphAPEC6_00180	WAE76893.1	2e-74 (100)	100		Escherichia phage ph0011
Gp34	12858:13070 (f)	8185	70	hypothetical protein UES1_256	UTS53624.1	3e-44 (98.57)	100		Escherichia phage UE-S1
Gp35	13158:13331 (f)	6600	57	hypothetical protein Ecwhy1_475	QAY00749.1	6e-33 (100)	100		Escherichia phage Ecwhy_1
Gp36	13328:13477 (f)	5751	49	hypothetical protein UES1_258	UTS53626.1	2e-26 (97.96)	100		Escherichia phage UE-S1
Gp37	13793:14068 (f)	10650	91	hypothetical protein Ecwhy1_473	QAY00747.1	4e-59 (100)	100		Escherichia phage Ecwhy_1
Gp38	14157:14339 (f)	6705	60	hypothetical protein G17_00018	QBO61514.1	3e-34 (98.33)	100		Escherichia phage vB_EcoM_G17
Gp39	14427:14750 (f)	12494	107	hypothetical protein EcMJ_037	WGM49280.1	4e-71 (98.13)	100		Escherichia phage vB_Ec-M-J
Gp40	14846:15241 (f)	14942	131	hypothetical protein Ecwhy1_470	QAY00745.1	1e-90 (100)	100		Escherichia phage Ecwhy_1
Gp41	15303:15557 (f)	9870	84	hypothetical protein Ecwhy1_469	QAY00744.1	1e-51 (100)	100		Escherichia phage Ecwhy_1
Gp42	15554:15829 (f)	10748	91	hypothetical protein UES1_263	UTS53631.1	7e-56 (93.41)	100		Escherichia phage UE-S1

Gp43	15830:16129 (f)	11046	99	hypothetical protein UES1_264	UTS53632.1	3e-64 (98.99)	100		Escherichia phage UE-S1
Gp44	16161:16745 (f)	22372	194	Serine/threonine-protein kinase PRP4	6QX9_K			5.5e-11(99.4)	Human adenovirus 2
Gp45	16836:16955 (f)	4662	39	hypothetical protein UES1_266	UTS53634.1	3e-08 (84.85)	84		Escherichia phage UE-S1
Gp46	16967:17188 (f)	8334	73	hypothetical protein A4_305	USL83381.1	2e-46 (98.63)	100		Escherichia phage A4
Gp47	17272:17676 (f)	15132	134	hypothetical protein vBEcoMphAPEC6_00245	WAE76906.1	4e-93 (97.76)	100		Escherichia phage ph0011
Gp48	17766:18038 (f)	9898	90	hypothetical protein vBEcoMphAPEC6_00250	WAE76907.1	1e-56 (96.67)	100		Escherichia phage ph0011
Gp49	18035:18226 (f)	7320	63	hypothetical protein ELP22_0062	WPK29762.1	2e-37 (98.41)	100		Escherichia phage BAU.Micro_ELP-22
Gp50	18314:18577 (f)	9488	87	hypothetical protein A4_309	USL83385.1	5e-55 (98.85)	100		Escherichia phage A4
Gp51	18645:18869 (f)	8760	74	hypothetical protein A4_310	USL83386.1	5e-46 (98.65)	100		Escherichia phage A4
Gp52	18946:19203 (f)	9768	85	hypothetical protein vBEcoMphAPEC6_00270	WAE76911.1	1e-57 (100)	100		Escherichia phage ph0011
Gp53	19294:19494 (f)	7320	66	hypothetical protein vBEcoMphAPEC6_00275	WAE76912.1	4e-39 (98.48)	100		Escherichia phage ph0011
Gp54	19740:20072 (f)	12478	110	hypothetical protein UES1_273	UTS53641.1	1e-74 (100)	100		Escherichia phage UE-S1
Gp55	20129:20563 (f)	16040	144	hypothetical protein vBEcoMphAPEC6_00285	WAE76914.1	3e-102 (100)	100		Escherichia phage ph0011
Gp56	20632:20841 (f)	7620	69	hypothetical protein vBEcoMphAPEC6_00290	WAE76915.1	1e-41 (100)	100		Escherichia phage ph0011
Gp57	21091:21288 (f)	7934	65	hypothetical protein Ecwhy1_454	QAY00729.1	4e-38 (100)	100		Escherichia phage Ecwhy_1
Gp58	21285:21485 (f)	8042	66	hypothetical protein EcMJ_055	WGM49298.1	3e-36 (98.44)	96		Escherichia phage vB_Ec-M-J
Gp59	21482:21691 (f)	8189	69	hypothetical protein Ecwhy1_452	QAY00727.1	6e-42 (100)	100		Escherichia phage Ecwhy_1
Gp60	21688:21900 (f)	8240	70	hypothetical protein Ecwhy1_451	QAY00726.1	2e-41 (100)	100		Escherichia phage Ecwhy_1
Gp61	21897:22109 (f)	8135	70	hypothetical protein EcMJ_058	WGM49301.1	3e-36 (94.37)	100		Escherichia phage vB_Ec-M-J
Gp62	22106:22324 (f)	8297	72	hypothetical protein vBEcoMphAPEC6_gp484c	QDF14107.1	2e-45 (98.61)	100		Escherichia phage vB_EcoM_phAPEC6
Gp63	22321:22539 (f)	8421	72	hypothetical protein vBEcoMphAPEC6_00325	WAE76922.1	8e-44 (95.83)	100		Escherichia phage ph0011

Gp64	22536:22793 (f)	10440	85	hypothetical protein Ecwhy1_448	QAY00723.1	4e-56 (100)	100		Escherichia phage Ecwhy_1
Gp65	22864:23022 (f)	6302	52	hypothetical protein EcMJ_061	WGM49304.1	9e-28 (94.23)	100		Escherichia phage vB_Ec-M-J
Gp66	23007:23258 (f)	9875	83	hypothetical protein EcMJ_062	WGM49305.1	4e-53 (97.59)	100		Escherichia phage vB_Ec-M-J
Gp67	23242:23457 (f)	8231	71	hypothetical protein Ecwhy1_445	QAY00720.1	9e-43 (97.18)	100		Escherichia phage Ecwhy_1
Gp68	23454:23672 (f)	8331	72	hypothetical protein Ecwhy1_444	QAY00719.1	1e-44 (97.22)	100		Escherichia phage Ecwhy_1
Gp69	23663:23911 (f)	9803	82	hypothetical protein UES1_288	UTS53656.1	1e-27 (72.60)	89		Escherichia phage UE-S1
Gp70	23904:24116 (f)	8131	70	hypothetical protein Ecwhy1_443	QAY00718.1	1e-40 (100)	100		Escherichia phage Ecwhy_1
Gp71	24113:24361 (f)	9891	82	hypothetical protein Ecwhy1_442	QAY00717.1	7e-49 (92.68)	100		Escherichia phage Ecwhy_1
Gp72	24339:24602 (f)	10553	87	hypothetical protein Ecwhy1_441	QAY00716.1	7e-56 (97.70)	100		Escherichia phage Ecwhy_1
Gp73	24599:24850 (f)	10086	83	hypothetical protein vBEcoMphAPEC6_00370	WAE76931.1	8e-42 (77.11)	100		Escherichia phage ph0011
Gp74	24847:25071 (f)	8850	74	hypothetical protein Ecwhy1_439	QAY00714.1	3e-43 (93.24)	100		Escherichia phage Ecwhy_1
Gp75	25061:25318 (f)	10275	85	hypothetical protein Ecwhy1_437	QAY00712.1	2e-54 (98.82)	100		Escherichia phage Ecwhy_1
Gp76	25287:25529 (f)	9513	80	hypothetical protein Ecwhy1_436	QAY00711.1	8e-47 (100)	98		Escherichia phage Ecwhy_1
Gp77	25526:25756 (f)	9362	76	hypothetical protein EcMJ_073	WGM49315.1	7e-47 (97.37)	100		Escherichia phage vB_Ec-M-J
Gp78	25734:25949 (f)	8382	71	hypothetical protein Ecwhy1_435	QAY00710.1	7e-42 (100)	100		Escherichia phage Ecwhy_1
Gp79	25946:26182 (f)	9075	78	hypothetical protein Ecwhy1_434	QAY00709.1	7e-48 (100)	100		Escherichia phage Ecwhy_1
Gp80	26146:26397 (f)	9598	83	hypothetical protein Ecwhy1_433	QAY00708.1	4e-52 (100)	100		Escherichia phage Ecwhy_1
Gp81	26358:26597 (f)	9251	79	hypothetical protein Ecwhy1_432	QAY00707.1	1e-50 (100)	100		Escherichia phage Ecwhy_1
Gp82	26594:26809 (f)	8426	71	hypothetical protein Ecwhy1_431	QAY00706.1	2e-41 (97.18)	100		Escherichia phage Ecwhy_1
Gp83	26806:27027 (f)	8541	73	hypothetical protein Ecwhy1_430	QAY00705.1	4e-47 (100)	100		Escherichia phage Ecwhy_1
Gp84	27020:27280 (f)	10708	86	hypothetical protein vBEcoMphAPEC6_gp467c	QDF14090.1	1e-49 (95.35)	100		Escherichia phage vB_EcoM_phAPEC6
Gp85	27270:27608 (f)	13356	112	hypothetical protein EcMJ_080	WGM49322.1	5e-75 (98.21)	100		Escherichia phage vB_Ec-M-J
Gp86	27623:27964 (f)	13616	113	hypothetical protein Ecwhy1_428	QAY00703.1	1e-73 (98.23)	100		Escherichia phage Ecwhy_1
Gp87	27968:28192 (f)	8588	74	hypothetical protein Ecwhy1_427	QAY00702.1	3e-45 (98.65)	100		Escherichia phage Ecwhy_1

Gp88	28156:28518 (f)	13673	120	hypothetical protein Ecwhy1_426	QAY00701.1	8e-81 (97.50)	100		Escherichia phage Ecwhy_1
Gp89	28578:28823 (f)	9345	81	hypothetical protein G17_00582	QBO62071.1	2e-51 (98.77)	100		Escherichia phage vB_EcoM_G17
Gp90	28823:29146 (f)	12526	107	hypothetical protein Ecwhy1_425	QAY00700.1	1e-70 (95.33)	100		Escherichia phage Ecwhy_1
Gp91	29156:29467 (f)	12037	103	hypothetical protein Ecwhy1_424	QAY00699.1	3e-67 (100.00)	100		Escherichia phage Ecwhy_1
Gp92	29496:29747 (f)	9928	83	hypothetical protein Ecwhy1_422	QAY00697.1	9e-49 (93.90)	98		Escherichia phage Ecwhy_1
Gp93	29719:30123 (f)	15632	134	hypothetical protein Ecwhy1_421	QAY00696.1	6e-94 (100)	100		Escherichia phage Ecwhy_1
Gp94	30111:30263 (f)	6230	50	hypothetical protein Ecwhy1_420	QAY00695.1	7e-28 (100)	100		Escherichia phage Ecwhy_1
Gp95	30263:30445 (f)	6964	60	hypothetical protein Ecwhy1_419	QAY00694.1	2e-33 (100)	100		Escherichia phage Ecwhy_1
Gp96	30457:31017 (f)	21443	186	hypothetical protein Ecwhy1_418	QAY00693.1	4e-133 (100)	100		Escherichia phage Ecwhy_1
Gp97	31094:31600 (f)	19729	168	hypothetical protein EcMJ_092	WGM49334.1	6e-120 (99.40)	100		Escherichia phage vB_Ec-M-J
Gp98	31590:31901 (f)	12348	103	hypothetical protein Ecwhy1_417	QAY00692.1	5e-66 (98.06)	100		Escherichia phage Ecwhy_1
Gp99	31907:32248 (f)	13425	113	hypothetical protein EcMJ_094	WGM49336.1	5e-73 (98.17)	96		Escherichia phage vB_Ec-M-J
Gp100	32220:32789 (f)	21885	189	hypothetical protein Ecwhy1_415	QAY00690.1	5e-138 (100)	100		Escherichia phage Ecwhy_1
Gp101	32776:33117 (f)	13572	113	hypothetical protein EcMJ_096	WGM49338.1	8e-72 (95.58)	100		Escherichia phage vB_Ec-M-J
Gp102	33096:33593 (f)	19804	165	hypothetical protein EcMJ_097	WGM49339.1	6e-116 (100)	98		Escherichia phage vB_Ec-M-J
Gp103	33668:34216 (f)	21376	182	hypothetical protein EcMJ_098	WGM49340.1	2e-124 (93.19)	100		Escherichia phage vB_Ec-M-J
Gp104	34213:34821 (f)	23431	202	hypothetical protein Ecwhy1_411	QAY00686.1	2e-142 (99.01)	100		Escherichia phage Ecwhy_1
Gp105	34911:35549 (f)	25061	212	hypothetical protein Ecwhy1_410	QAY00685.1	5e-147 (98.03)	95		Escherichia phage Ecwhy_1
Gp106	35546:35890 (f)	13062	114	hypothetical protein PSLUR01_00262	SCA80239.1	6e-72 (96.49)	100		Escherichia phage vB_Eco_slurp01
Gp107	35901:36455 (f)	20576	184	hypothetical protein EcMJ_102	WGM49344.1	2e-130 (99.46)	100		Escherichia phage vB_Ec-M-J
Gp108	36465:36626 (f)	6346	53	hypothetical protein EO157G_3400	BBM61929.1	4e-26 (94.23)	98		Escherichia phage SP27
Gp109	36619:36822 (f)	8212	67	hypothetical protein Ecwhy1_407	QAY00682.1	5e-41 (100)	100		Escherichia phage Ecwhy_1
Gp110	36809:37024 (f)	8399	71	hypothetical protein Ecwhy1_406	QAY00681.1	2e-43 (100)	100		Escherichia phage Ecwhy_1
Gp111	37221:37598 (f)	14799	125	hypothetical protein ACQ29_gp195	YP_009150509.1	1e-67 (77.60)	100		Escherichia phage PBECO4
Gp112	37598:37954 (f)	13973	118	hypothetical protein EcMJ_106	WGM49348.1	3e-79 (97.46)	100		Escherichia phage vB_Ec-M-J

Gp113	37951:38316 (f)	14314	121	hypothetical protein vBEcoMphAPEC6_00540	WAE76965.1	9e-80 (96.69)	100		Escherichia phage ph0011
Gp114	38318:38662 (f)	13777	114	hypothetical protein EcMJ_107	WGM49349.1	5e-73 (95.61)	100		Escherichia phage vB_Ec-M-J
Gp115	38659:38844 (f)	7562	61	hypothetical protein EcMJ_108	WGM49350.1	6e-36 (100)	100		Escherichia phage vB_Ec-M-J
Gp116	38846:39316 (f)	18455	156	hypothetical protein EcMJ_109	WGM49351.1	2e-108 (98.72)	100		Escherichia phage vB_Ec-M-J
Gp117	39313:39753 (f)	16790	146	hypothetical protein EcMJ_110	WGM49352.1	6e-103 (99.32)	100		Escherichia phage vB_Ec-M-J
Gp118	39756:40247 (f)	19180	163	hypothetical protein Ecwhy1_399	QAY00674.1	2e-113 (100)	100		Escherichia phage Ecwhy_1
Gp119	40222:40665 (f)	17387	147	hypothetical protein EO157G_3500	BBM61939.1	5e-102 (100)	100		Escherichia phage SP27
Gp120	40662:40976 (f)	12983	104	hypothetical protein EO157G_3510	BBM61940.1	2e-66 (95.19)	100		Escherichia phage SP27
Gp121	40976:41419 (f)	17480	147	hypothetical protein vBEcoMphAPEC6_00580	WAE76973.1	8e-96 (96.60)	100		Escherichia phage ph0011
Gp122	41416:41862 (f)	17124	148	hypothetical protein Ecwhy1_395	QAY00670.1	3e-98 (98.64)	99		Escherichia phage Ecwhy_1
Gp123	41859:42392 (f)	20702	177	hypothetical protein vBEcoMphAPEC6_00590	WAE76975.1	8e-95 (77.65)	100		Escherichia phage ph0011
Gp124	42406:42588 (f)	7333	60	hypothetical protein EcMJ_117	WGM49359.1	2e-37 (100)	100		Escherichia phage vB_Ec-M-J
Gp125	42566:42757 (f)	7595	63	hypothetical protein EcMJ_118	WGM49360.1	2e-35 (100.)	100		Escherichia phage vB_Ec-M-J
Gp126	42747:43301 (f)	21583	184	hypothetical protein Ecwhy1_391	QAY00666.1	3e-130(98.91)	100		Escherichia phage Ecwhy_1
Gp127	43292:43894 (f)	23367	200	hypothetical protein Ecwhy1_390	QAY00665.1	1e-144 (99.50)	100		Escherichia phage Ecwhy_1
Gp128	43900:44259 (f)	14482	119	hypothetical protein vBEcoMphAPEC6_00610	WAE76979.1	5e-77 (94.12)	100		Escherichia phage ph0011
Gp129	44249:44587 (f)	13256	112	hypothetical protein EcMJ_122	WGM49364.1	2e-74 (98.21)	100		Escherichia phage vB_Ec-M-J
Gp130	44595:45128 (f)	21186	177	hypothetical protein EcMJ_123	WGM49365.1	2e-126 (99.44)	100		Escherichia phage vB_Ec-M-J
Gp131	45188:45667 (f)	18832	159	hypothetical protein Ecwhy1_386	QAY00661.1	7e-108 (97.50)	100		Escherichia phage Ecwhy_1
Gp132	45664:46146 (f)	18817	160	hypothetical protein Ecwhy1_385	QAY00660.1	5e-109 (93.75)	100		Escherichia phage Ecwhy_1
Gp133	46139:46501 (f)	14745	120	hypothetical protein EcMJ_126	WGM49368.1	3e-81 (100)	100		Escherichia phage vB_Ec-M-J
Gp134	46485:46898 (f)	15951	137	hypothetical protein Ecwhy1_383	QAY00658.1	5e-96 (98.54)	100		Escherichia phage Ecwhy_1
Gp135	46898:47380 (f)	19360	160	hypothetical protein Ecwhy1_382	QAY00657.1	3e-112 (99.38)	100		Escherichia phage Ecwhy_1

Gp136	47364:47906 (f)	21087	180	hypothetical protein Ecwhy1_381	QAY00656.1	1e-122 (96.67)	100		Escherichia phage Ecwhy_1
Gp137	47903:48460 (f)	21729	185	hypothetical protein EcMJ_130	WGM49372.1	5e-134 (100)	100		Escherichia phage vB_Ec-M-J
Gp138	48453:48971 (f)	19955	172	hypothetical protein Ecwhy1_379	QAY00654.1	3e-122 (100)	100		Escherichia phage Ecwhy_1
Gp139	49059:49622 (f)	22055	187	hypothetical protein Ecwhy1_378	QAY00653.1	5e-129 (98.93)	100		Escherichia phage Ecwhy_1
Gp140	49622:50206 (f)	22847	194	hypothetical protein Ecwhy1_377	QAY00652.1	3e-142 (100)	100		Escherichia phage Ecwhy_1
Gp141	50193:50771 (f)	22097	192	hypothetical protein Ecwhy1_376	QAY00651.1	2e-139 (100)	100		Escherichia phage Ecwhy_1
Gp142	50771:51199 (f)	16655	142	hypothetical protein Ecwhy1_375	QAY00650.1	6e-99 (100)	100		Escherichia phage Ecwhy_1
Gp143	51196:51702 (f)	19538	168	hypothetical protein Ecwhy1_374	QAY00649.1	9e-117 (100)	100		Escherichia phage Ecwhy_1
Gp144	51689:52270 (f)	22913	193	hypothetical protein Ecwhy1_373	QAY00648.1	2e-140 (100)	100		Escherichia phage Ecwhy_1
Gp145	52282:52833 (f)	21133	183	hypothetical protein EcMJ_138	WGM49380.1	7e-134 (99.45)	100		Escherichia phage vB_Ec-M-J
Gp146	52830:53432 (f)	23088	200	hypothetical protein Ecwhy1_371	QAY00646.1	3e-147 (100)	100		Escherichia phage Ecwhy_1
Gp147	53422:54006 (f)	22887	194	hypothetical protein Ecwhy1_370	QAY00645.1	3e-137 (100)	100		Escherichia phage Ecwhy_1
Gp148	54016:54429 (f)	15802	137	hypothetical protein Ecwhy1_369	QAY00644.1	2e-93 (98.54)	100		Escherichia phage Ecwhy_1
Gp149	54429:54947 (f)	20351	172	hypothetical protein Ecwhy1_368	QAY00643.1	2e-120 (100)	100		Escherichia phage Ecwhy_1
Gp150	54941:55414 (f)	18534	157	hypothetical protein Ecwhy1_367	QAY00642.1	5e-108 (100)	100		Escherichia phage Ecwhy_1
Gp151	55428:55775 (f)	12949	115	hypothetical protein Ecwhy1_366	QAY00641.1	1e-78 (100)	100		Escherichia phage Ecwhy_1
Gp152	55762:56262 (f)	18655	166	hypothetical protein Ecwhy1_365	QAY00640.1	3e-117(100)	100		Escherichia phage Ecwhy_1
Gp153	56305:56778 (f)	18140	157	hypothetical protein vBEcoMphAPEC6_00735	WAE77004.1	2e-102(93.63)	100		Escherichia phage ph0011
Gp154	56781:56912 (f)	5138	43	hypothetical protein Ecwhy1_363	QAY00638.1	2e-21(100)	100		Escherichia phage Ecwhy_1
Gp155	56946:57794 (f)	32861	282	hypothetical protein Ecwhy1_362	QAY00637.1	0.0 (100)	100		Escherichia phage Ecwhy_1
Gp156	57811:58104 (f)	11503	97	hypothetical protein EcMJ_149	WGM49391.1	3e-65 (98.97)	100		Escherichia phage vB_Ec-M-J
Gp157	58176:58562 (f)	14515	128	hypothetical protein Ecwhy1_360	QAY00635.1	4e-89 (100)	100		Escherichia phage Ecwhy_1
Gp158	58614:58919 (f)	11854	101	hypothetical protein Ecwhy1_359	QAY00634.1	2e-68 (99.01)	100		Escherichia phage Ecwhy_1
Gp159	58971:59819 (f)	33361	282	hypothetical protein Ecwhy1_358	QAY00633.1	0.0 (100)	100		Escherichia phage Ecwhy_1

Gp160	60186:60359 (f)	6774	57	hypothetical protein	WPK18348.1	3e-21 (89.58)	84		Salmonella phage SD-2_S15
Gp161	60505:61047 (f)	21069	180	hypothetical protein Ecwhy1_357	QAY00632.1	5e-129 (100)	100		Escherichia phage Ecwhy_1
Gp162	61064:61588 (f)	19620	174	DNA methyltransferase	QAY00631.1	3e-124(100.00)	100	3.1(83.55)	Escherichia phage Ecwhy_1
Gp163	61585:62118 (f)	20524	177	ribonucleotide reductase A	QAY00630.1	1e-128 (100)	100		Escherichia phage Ecwhy_1
Gp164	62121:62414 (f)	10961	97	hypothetical protein vBEcoMphAPEC6_00785	WAE77014.1	3e-54 (83.51)	100		Escherichia phage ph0011
Gp165	62407:62694 (f)	11312	95	hypothetical protein Ecwhy1_353	QAY00628.1	3e-63 (100)	100		Escherichia phage Ecwhy_1
Gp166	62675:63289 (f)	23184	204	hypothetical protein Ecwhy1_352	QAY00627.1	8e-147 (99.51)	100		Escherichia phage Ecwhy_1
Gp167	63336:63605 (f)	10768	89	hypothetical protein Ecwhy1_351	QAY00626.1	3e-57 (100)	100		Escherichia phage Ecwhy_1
Gp168	63602:63862 (f)	10101	86	hypothetical protein Ecwhy1_350	QAY00625.1	2e-54 (100)	100		Escherichia phage Ecwhy_1
Gp169	63859:64170 (f)	12266	103	hypothetical protein vBEcoMphAPEC6_00810	WAE77019.1	9e-59 (85.44)	100		Escherichia phage ph0011
Gp170	64181:64492 (f)	12305	103	hypothetical protein Ecwhy1_348	QAY00623.1	6e-68 (100)	100		Escherichia phage Ecwhy_1
Gp171	64455:64739 (f)	11193	94	hypothetical protein Ecwhy1_347	QAY00622.1	7e-61 (100)	100		Escherichia phage Ecwhy_1
Gp172	64732:64932 (f)	7837	66	hypothetical protein EcMJ_165	WGM49407.1	1e-41 (98.48)	100		Escherichia phage vB_Ec-M-J
Gp173	64925:65188 (f)	10252	87	hypothetical protein Ecwhy1_346	QAY00621.1	2e-52 (100)	100		Escherichia phage Ecwhy_1
Gp174	65185:65475 (f)	11564	96	hypothetical protein Ecwhy1_345	QAY00620.1	6e-64 (100)	100		Escherichia phage Ecwhy_1
Gp175	65472:65645 (f)	6422	57	hypothetical protein Ecwhy1_344	QAY00619.1	1e-31 (100)	100		Escherichia phage Ecwhy_1
Gp176	65635:65919 (f)	11327	94	hypothetical protein Ecwhy1_343	QAY00618.1	3e-62 (100)	100		Escherichia phage Ecwhy_1
Gp177	65978:66706 (f)	28484	242	hypothetical protein vBEcoMphAPEC6_00855	WAE77028.1	1e-174 (96.69)	100		Escherichia phage ph0011
Gp178	66716:66997 (f)	10954	93	hypothetical protein EcMJ_172	WGM49414.1	3e-59(98.92)	100		Escherichia phage vB_Ec-M-J
Gp179	67042:67341 (f)	11239	99	hypothetical protein Ecwhy1_340	QAY00616.1	2e-64 (100)	100		Escherichia phage Ecwhy_1
Gp180	67346:68035 (f)	26936	229	hypothetical protein	WIL00688.1	2e-159 (92.14)	100		Escherichia phage vB_EcoM_CRJP21
Gp181	68037:68300 (f)	10030	87	hypothetical protein Ecwhy1_338	QAY00614.1	2e-55 (100)	100		Escherichia phage Ecwhy_1
Gp182	68297:69439 (f)	44223	380	RNA ligase	QAY00613.1	0.0 (100)	100		Escherichia phage Ecwhy_1

Gp183	69469:69723 (f)	9923	84	hypothetical protein vBEcoMphAPEC6_00890	WAE77035.1	1e-48 (91.67)	100		Escherichia phage ph0011
Gp184	69733:69969 (f)	9097	78	hypothetical protein Ecwhy1_335	QAY00611.1	2e-49 (100)	100		Escherichia phage Ecwhy_1
Gp185	69947:70258 (f)	12007	103	Transcriptional regulator	7DE9			0.52(90.98)	Escherichia coli BL21(DE3)
Gp186	70262:71194 (f)	35918	310	polynucleotide kinase	YP_009150439.1	0.0 (100)	98		Escherichia phage PBECO4
Gp187	71244:71420 (f)	7139	58	hypothetical protein ACQ29_gp124	YP_009150438.1	3e-27 (86.21)	100		Escherichia phage PBECO4
Gp188	71420:71680 (f)	10132	86	hypothetical protein Ecwhy1_332	QAY00608.1	6e-55 (100)	100		Escherichia phage Ecwhy_1
Gp189	71680:71931 (f)	10055	83	hypothetical protein Ecwhy1_331	QAY00607.1	4e-53 (100)	100		Escherichia phage Ecwhy_1
Gp190	71924:72226 (f)	11600	100	hypothetical protein Ecwhy1_330	QAY00606.1	2e-65 (100)	100		Escherichia phage Ecwhy_1
Gp191	72223:72519 (f)	11393	98	SAM-dependent 2'-O-MTase domain-containing protein	WAE77043.1	6e-59 (94.90)	100		Escherichia phage ph0011
Gp192	72520:72825 (f)	11726	101	hypothetical protein vBEcoMphAPEC6_00935	WAE77044.1	3e-59 (91.09)	100		Escherichia phage ph0011
Gp193	72883:73212 (f)	12444	109	hypothetical protein EcMJ_187	WGM49429.1	4e-66 (99.02)	93		Escherichia phage vB_Ec-M-J
Gp194	73187:73783 (f)	21325	198	Ribosome biogenesis protein Nop10	2APO_B			0.002(96.4)	Methanocaldococcus jannaschii
Gp195	73786:74118 (f)	13119	110	hypothetical protein EcMJ_189	WGM49431.1	2e-71 (98.18)	100		Escherichia phage vB_Ec-M-J
Gp196	74121:74336 (f)	8269	71	membrane protein	WGM49432.1	5e-43(100)			Escherichia phage vB_Ec-M-J
Gp197	74336:74590 (f)	9861	84	hypothetical protein vBEcoMphAPEC6_00960	WAE77049.1	4e-52 (96.39)	100		Escherichia phage ph0011
Gp198	74590:75333 (f)	28665	247	serine/threonine-protein phosphatase	QAY00599.1	0.0 (100)	100		Escherichia phage Ecwhy_1
Gp199	75338:75607 (f)	10308	89	hypothetical protein Ecwhy1_321	QAY00598.1	3e-55(100.00)	100		Escherichia phage Ecwhy_1
Gp200	75618:75845 (f)	8665	75	chaperone protein	EES8153952.1	3e-40 (84.00)	100		Escherichia phage ph0011
Gp201	75842:76255 (f)	15285	137	hydrolase	WGM49437.1	7e-98 (99.27)	100		Escherichia phage vB_Ec-M-J
Gp202	76255:77454 (f)	46300	399	hypothetical protein Ecwhy1_318	QAY00595.1	0.0(100)	100		Escherichia phage Ecwhy_1
Gp203	77466:78884 (f)	53042	472	DNA ligase	QAY00594.1	0.0 (100)	100		Escherichia phage Ecwhy_1
Gp204	78904:79695 (f)	30388	263	calcineurin-like phosphoesterase	QAY00593.1	0.0 (100.00%)	100		Escherichia phage Ecwhy_1

Gp205	79705:80100 (f)	15302	131	hypothetical protein Ecwhy1_315	QAY00592.1	2e-91 (100)	100		Escherichia phage Ecwhy_1
Gp206	80100:80483 (f)	15166	127	hypothetical protein Ecwhy1_314	QAY00591.1	9e-85 (100)	100		Escherichia phage Ecwhy_1
Gp207	80543:81238 (f)	25444	231	protease	QAY00590.1	3e-170 (100)	100		Escherichia phage Ecwhy_1
Gp208	81290:81580 (f)	10756	96	Phosphoribosyl-ATP pyrophosphatase	1YVW_B			3.2e-13(99.47)	Bacillus cereus
Gp209	81582:82028 (f)	17139	148	hypothetical protein UES1_418	UTS53785.1	2e-100 (91.89)	100		Escherichia phage UE-S1
Gp210	82148:82666 (f)	20691	172	hypothetical protein Ecwhy1_310	QAY00587.1	3e-122 (100)	100		Escherichia phage Ecwhy_1
Gp211	82668:83195 (f)	19739	175	structural protein	QAY00586.1	6e-125 (100)	100		Escherichia phage Ecwhy_1
Gp212	83152:83625 (f)	17510	157	CMP/dCMP deaminase	QAY00585.1	1e-113 (100)	100		Escherichia phage Ecwhy_1
Gp213	83622:83888 (f)	10154	88	hypothetical protein PBI_121Q_470	YP_009102057.1	8e-31 (63.95)	100		Escherichia phage 121Q
Gp214	84170:84778 (f)	22548	202	head maturation protease	WGM49450.1	3e-144(99.50)	100		Escherichia phage vB_Ec-M-J
Gp215	84871:85131 (f)	9749	86	hypothetical protein Ecwhy1_305	QAY00584.1	2e-53 (100)	100		Escherichia phage Ecwhy_1
Gp216	85128:85721 (f)	22009	197	protease	QAY00583.1	2e-143 (100)	100		Escherichia phage Ecwhy_1
Gp217	85753:85944 (f)	7746	63	hypothetical protein vBEcoMphAPEC6_01060	WAE77069.1	3e-10 (54.17)	76		Escherichia phage ph0011
Gp218	85954:86706 (f)	28554	250	Sir2-like protein	QAY00582.1	0.0 (99.60)	100		Escherichia phage Ecwhy_1
Gp219	86703:87254 (f)	20769	183	NUDIX hydrolase	WGM49454.1	2e-132 (98.91)	100		Escherichia phage vB_Ec-M-J
Gp220	87264:87584 (f)	12477	106	hypothetical protein vBEcoMphAPEC6_01075	WAE77072.1	4e-62 (83.96)	100		Escherichia phage ph0011
Gp221	87581:87883 (f)	11395	100	hypothetical protein Ecwhy1_300	QAY00579.1	5e-68 (100)	100		Escherichia phage Ecwhy_1
Gp222	87945:89384 (f)	53190	479	PhoH-like protein	QAY00578.1	0.0 (100)	100		Escherichia phage Ecwhy_1
Gp223	89451:89648 (f)	7526	65	hypothetical protein Ecwhy1_298	QAY00577.1	2e-36 (100)	100		Escherichia phage Ecwhy_1
Gp224	89865:90422 (f)	22918	185	hypothetical protein Ecwhy1_297	QAY00576.1	7e-136 (100)	100		Escherichia phage Ecwhy_1
Gp225	90450:90779 (f)	12864	109	hypothetical protein Ecwhy1_296	QAY00575.1	9e-73 (100)	100		Escherichia phage Ecwhy_1
Gp226	90779:91426 (f)	25131	215	viral protein-winged helix complex, winged helix, dna-binding	2CO5_A	9e-160(100)		0.35(90.44)	Sulfolobus turreted icosahedral virus 1

Gp227	91444:91548 (f)	4231	34	hypothetical protein UES1_434	UTS53801.1	UTS53801.1	2e-12 (94.12)	100	Escherichia phage UE-S1
Gp228	91558:92529 (f)	37341	323	WGR domain-containing protein	YP_0091504 00.1	0.0 (98.45)		100	Escherichia phage PBECO4
Gp229	92526:92774 (f)	9486	82	Lytic conversion lipoprotein	8A60_B			0.052(94.13)	Escherichia phage T5
Gp230	92771:92941 (f)	6277	56	hypothetical protein Ecwhy1_292	QAY00571. 1	3e-32 (100)	100		Escherichia phage Ecwhy_1
Gp231	92938:93297 (f)	14187	119	hypothetical protein Ecwhy1_292	QAY00571. 1	8e-84 (100)	100		Escherichia phage Ecwhy_1
Gp232	93343:93957 (f)	23832	204	hypothetical protein Ecwhy1_290	QAY00569. 1	4e-149 (100)	100		Escherichia phage Ecwhy_1
Gp233	93954:94322 (f)	13802	122	CMP/dCMP-type deaminase domain-containing protein	WAE77083. 1	3e-83 (99.18)	100		Escherichia phage ph0011
Gp234	94319:94540 (f)	8921	73	hypothetical protein Ecwhy1_288	QAY00567. 1	5e-46 (100)	100		Escherichia phage Ecwhy_1
Gp235	94551:94910 (f)	13375	119	hypothetical protein vBEcoMphAPEC6_ 01140	WAE77085. 1	1e-55 (73.95)	100		Escherichia phage ph0011
Gp236	95141:95551 (f)	15785	136	hypothetical protein vBEcoMphAPEC6_ 01145	WAE77086. 1	8e-86 (93.38)	100		Escherichia phage ph0011
Gp237	95541:95726 (f)	7106	61	hypothetical protein Sharanji_gp463	WNN14744. 1	6e-36 (98.36)	100		Escherichia phage Sharanji
Gp238	95713:95829 (f)	4111	38	hypothetical protein EcMJ_230	WGM49472 .1	8e-16 (100)	100		Escherichia phage vB_Ec-M-J
Gp239	95989:97794 (f)	67767	601	nucleotide reductase	QAY00562. 1|	0.0 (100)	100		Escherichia phage Ecwhy_1
Gp240	97803:98129 (f)	12330	108	DUF4326 domain-containing protein	WAE77091. 1	4e-71 (96.30)	100		Escherichia phage ph0011
Gp241	98107:98577 (f)	17776	156	nucleotide reductase-activating protein	QAY00560. 1	4e-111 (100)	100		Escherichia phage Ecwhy_1
Gp242	98654:101350 (f)	98242	898	hypothetical protein Ecwhy1_280	QAY00559. 1	0.0 (100)	100		Escherichia phage Ecwhy_1
Gp243	101363:101950 (f)	21199	195	hypothetical protein Ecwhy1_279	QAY00558. 1	9e-137 (100)	100		Escherichia phage Ecwhy_1
Gp244	101967:104696 (f)	98879	909	hypothetical protein Ecwhy1_278	QAY00557. 1	0.0 (100)	100		Escherichia phage Ecwhy_1
Gp245	104767:105333 (f)	22243	188	hypothetical protein ACQ29_gp070	YP_0091503 84.1	1e-127 (95.21)	100		Escherichia phage PBECO4
Gp246	105346:105861 (f)	20019	171	hypothetical protein	AXC36864. 1	8e-111 (91.81)	100		Escherichia phage UB
Gp247	105858:106367 (f)	19882	169	hypothetical protein EcMJ_239	WGM49481 .1	5e-113(94.67)	100		Escherichia phage vB_Ec-M-J
Gp248	106370:106771 (f)	15719	133	hypothetical protein Ecwhy1_274	QAY00553. 1	4e-91 (100)	100		Escherichia phage Ecwhy_1
Gp249	106768:108006 (f)	48911	412	nucleotidyltransfera se	QAY00552. 1	0.0 (100)	100		Escherichia phage Ecwhy_1

Gp250	108017:108433 (f)	16829	138	D-alanyl carrier protein 1	7R49_F			4.3e-8(98.87)	Lactiplantibacillus plantarum subsp.
Gp251	108487:108861 (f)	14102	124	Cell division protein FtsB	8HHF_B			2.1(91)	Escherichia coli K-12
Gp252	108858:109172 (f)	11832	104	Lytic conversion lipoprotein	8A60_B			0.0023(96.59)	
Gp253	109175:109786 (f)	23984	203	HNH endonuclease	QAY00548.1	1e-150 (100)	100		Escherichia phage Ecwhy_1
Gp254	109841:110197 (f)	14342	118	hypothetical protein EcMJ_246	WGM49488.1	4e-81 (99.15)	100		Escherichia phage vB_Ec-M-J
Gp255	110197:110595 (f)	15308	132	hypothetical protein Sharanji_gp443	WNN14724.1	1e-78 (90.15)	100		Escherichia phage Sharanji
Gp256	110700:110816 (f)	4622	38	hypothetical protein	WPK18241.1	3e-16 (86.84)	100		Salmonella phage SD-2_S15
Gp257	111050:111265 (f)	8602	71	hypothetical protein vBEcoMphAPEC6_01640	WAE77185.1	6e-06 (74.19)	43		Escherichia phage ph0011
Gp258	111286:111714 (f)	16625	142	hypothetical protein EcMJ_248	WGM49490.1	5e-97 (99.30)	100		Escherichia phage vB_Ec-M-J
Gp259	111745:112185 (f)	16828	146	starvation-inducible DNA-binding protein	WAE77108.1	2e-95 (91.78)	100		Escherichia phage ph0011
Gp260	112234:112851 (f)	23211	205	hypothetical protein Ecwhy1_264	QAY00543.1	1e-147 (100)	100		Escherichia phage Ecwhy_1
Gp261	112868:113221 (f)	13315	117	hypothetical protein EcMJ_250	WGM49492.1	4e-78 (97.44)	100		Escherichia phage vB_Ec-M-J
Gp262	113202:113594 (f)	15176	130	hypothetical protein vBEcoMphAPEC6_01265	WAE77110.1	5e-89 (99.23)	100		Escherichia phage ph0011
Gp263	113591:114178 (f)	22691	195	hypothetical protein Ecwhy1_261	QAY00540.1	7e-135 (100.00)	94		Escherichia phage Ecwhy_1
Gp264	114192:114473 (f)	10859	93	hypothetical protein Ecwhy1_260	QAY00539.1	8e-62 (100.00)	100		Escherichia phage Ecwhy_1
Gp265	114482:114961 (f)	18979	159	hypothetical protein Ecwhy1_259	QAY00538.1	7e-114 (100.00)	100		Escherichia phage Ecwhy_1
Gp266	114951:115289 (f)	13275	112	hypothetical protein Ecwhy1_258	QAY00537.1	8e-75 (100.00)	100		Escherichia phage Ecwhy_1
Gp267	115282:115866 (f)	21974	194	thymidine kinase	WAE77115.1	1e-134 (96.91)	100		Escherichia phage ph0011
Gp268	115859:116083 (f)	8458	74	hypothetical protein Ecwhy1_256	QAY00535.1	4e-43 (100.00)	100		Escherichia phage Ecwhy_1
Gp269	116127:116570 (f)	17196	147	hypothetical protein Ecwhy1_255	QAY00534.1	3e-105 (100.00)	100		Escherichia phage Ecwhy_1
Gp270	116573:116962 (f)	15913	129	hypothetical protein Ecwhy1_254	QAY00533.1	5e-88 (100.00)	100		Escherichia phage Ecwhy_1
Gp271	116992:117336 (f)	13377	114	SAM hydrolase	7OCK_A			11(88.22)	Teetrevirus T3

Gp272	117348:117479 (f)	4855	43	Glycoprotein E2; E1-E2 glycoprotein, nucleocapsid protein, transmembrane helix, virus; 5.0A	3J2W_R			4.6(80.5)	Chikungunya virus
Gp273	117483:117686 (f)	7750	67	hypothetical protein A4_526	USL83602.1	1e-39(97.01)	100		Escherichia phage A4
Gp274	117694:118104 (f)	16360	136	Acb1; Anti-CBASS, Nuclease, Immune evasion, viral protein;	7T26_A			0.0059(97.56)	Erwinia phage FBB1
Gp275	118272:119198 (f)	35178	308	lysozyme	WGM49506 .1	0.0 (99.35)	100		Escherichia phage vB_Ec-M-J
Gp276	119214:119546 (f)	12227	110	putative sigma 54	QAY00527. 1	5e-73 (99.09)	100		Escherichia phage Ecwhy_1
Gp277	119601:119981 (f)	15078	126	hypothetical protein Ecwhy1_247	QAY00526. 1	1e-85 (99.21)	100		Escherichia phage Ecwhy_1
Gp278	119994:120365 (f)	14756	123	hypothetical protein Ecwhy1_246	QAY00525. 1	2e-81 (98.37)	100		Escherichia phage Ecwhy_1
Gp279	120365:120490 (f)	4906	41	hypothetical protein ACQ29_gp034	YP_0091503 48.1	3e-07 (70.27)	100		Escherichia phage PBECO4
Gp280	120490:120675 (f)	6725	61	hypothetical protein Ecwhy1_244	QAY00523. 1	9e-35 (100.00)	100		**Escherichia phage Ecwhy_1**
Gp281	120672:121079 (f)	16221	135	hypothetical protein ACQ29_gp036	YP_0091503 50.1	2e-87 (94.81)	100		Escherichia phage PBECO4
Gp282	121066:121488 (f)	16620	140	hypothetical protein EcMJ_269	WGM49511 .1	1e-97 (99.29)	100		Escherichia phage vB_Ec-M-J
Gp283	121500:121622 (f)	4881	40	hypothetical protein Ecwhy1_241	QAY00520. 1	2e-18 (97.44)	100		Escherichia phage Ecwhy_1
Gp284	121624:122739 (f)	43049	371	tRNA nucleotidyltransfera se	WGM49513 .1	0.0 (99.19)	100		Escherichia phage vB_Ec-M-J
Gp285	122849:123259 (f)	16010	136	nudix hydrolase	QAY00518. 1	3e-96 (99.26)	100		Escherichia phage Ecwhy_1
Gp286	123249:123497 (f)	9009	82	Thioredoxin glutathione reductase	7B02_A			8e-8(98.9)	Escherichia coli BL21(DE3)
Gp287	123497:123739 (f)	9077	80	hypothetical protein vBEcoMphAPEC6_ 01375	WAE77132. 1	1e-32 (72.50)	100		Escherichia phage ph0011
Gp288	123785:124027 (f)	9257	80	hypothetical protein vBEcoMphAPEC6_ 01380	WAE77133. 1	9e-49 (95.00)	100		Escherichia phage ph0011
Gp289	124079:124555 (f)	18059	158	phosphatase	WGM49518 .1	7e-112 (98.10)	100		Escherichia phage vB_Ec-M-J
Gp290	124575:124883 (f)	11128	102	hypothetical protein	WOL22725. 1	2e-58 (89.22)			Escherichia phage vB_EcoM_JNE01
Gp291	124896:125273 (f)	14089	125	aminoacyl-tRNA hydrolase	WAE77136. 1	8e-81 (93.60)	100		Escherichia phage ph0011

Gp292	125535:126326 (f)	29859	263	neck protein	WAE77137.1	0.0 (98.48)	100		Escherichia phage ph0011
Gp293	126407:126685 (f)	10585	92	hypothetical protein	QXN76268.1	5e-59(97.83)	100		Escherichia phage BF17
Gp294	126704:127330 (f)	23896	208	putative deoxynucleoside monophosphate kinase	YP_009150337.1	2e-138 (88.89)	99		Escherichia phage PBECO4
Gp295	127425:130088 (f)	96678	887	tail sheath protein	QXN76270.1	0.0 (99.89)	100		Escherichia phage BF17
Gp296	130172:130318 (f)	5687	48	coat protein; helical polymer, cumulative disorder, plant viruses, viral protein; 5.6A	5A2T_E			2.6(59.17)	BAMBOO MOSAIC VIRUS
Gp297	130476:131162 (f)	25055	228	Tail tube protein gp19; T4, baseplate-tail tube complex, pre-attachment, bacteriophage, bacterial virus, hexagonal, memb	5IV5_p			4.3e-23(99.91)	Tequatrovirus T4
Gp298	131252:132028 (f)	28881	258	tail tube protein	WGM49527.1	0.0 (99.61)	100		Escherichia phage vB_Ec-M-J
Gp299	132040:133134 (f)	40260	364	Tail tube protein gp19; T4, baseplate-tail tube complex, pre-attachment, bacteriophage, bacterial virus, hexagonal, memb	5IV5_p			7.4e-17(99.75)	Tequatrovirus T4
Gp300	133135:133563 (f)	16504	142	head closure	YP_009150332.1	2e-93 (91.55)	100		Escherichia phage PBECO4
Gp301	133594:134151 (f)	21112	185	Terminase DNA packaging enzyme small subunit; helix, small terminase, viral protein; 1.81A	3TXS_A			0.94(94.02)	Biquartavirus 44RR2
Gp302	134179:134712 (f)	20558	177	structural protein	QAY00501.1	7e-128 (100.00)	100		Escherichia phage Ecwhy_1
Gp303	134716:135261 (f)	20101	181	Hsp70 heat shock protein	YP_009150329.1	1e-105 (85.64)	100		Escherichia phage PBECO4
Gp304	135275:136786 (f)	56337	503	structural protein	QAY00499.1	0.0 (99.80)	100		Escherichia phage Ecwhy_1
Gp305	136809:137798 (f)	36097	329	virion structural protein	WGM49534.1	0.0 (99.70)	100		Escherichia phage vB_Ec-M-J
Gp306	137800:138192 (f)	14429	130	virion structural protein	YP_009150326.1	6e-55 (71.43)	99		Escherichia phage PBECO4

Gp307	138196:138573 (f)	14555	125	Transcriptional regulator HdfR; Structural Genomics	6WN5_A			3.1(76.43)	Klebsiella pneumoniae subsp. Pneumoniae
Gp308	138890:140218 (f)	49592	442	ATPase	YP_009150324.1	0.0 (93.67)	100		Escherichia phage PBECO4
Gp309	140294:141703 (f)	53529	469	HNH endonuclease	WGM49538.1	0.0 (99.79)	100		Escherichia phage vB_Ec-M-J
Gp310	141813:142043 (f)	8259	76	hypothetical protein Ecwhy1_214	QAY00493.1	3e-43 (100.00)	100		Escherichia phage Ecwhy_1
Gp311	142046:142699 (f)	24721	217	hypothetical protein Ecwhy1_213	QAY00492.1	1e-160 (100.00)	100		Escherichia phage Ecwhy_1
Gp312	142705:143301 (f)	22301	198	DUF2493 domain-containing protein	WAE77158.1	1e-134 (91.37)	100		Escherichia phage ph0011
Gp313	143298:144227 (f)	35522	309	thymidylate synthase	QXN76288.1	0.0 (99.68)	100		Escherichia phage BF17
Gp314c	144260:146224 (r)	70132	654	structural protein	QAY00489.1	0.0 (100.00)	100		Escherichia phage Ecwhy_1
Gp315c	146316:147095 (r)	29221	259	structural protein	QAY00488.1	0.0 (99.61)	100		Escherichia phage Ecwhy_1
Gp316c	147162:152882 (r)	2E+05	##	long tail fiber proximal subunit	QAY00487.1	0.0 (98.64)	100		Escherichia phage Ecwhy_1
Gp317c	153032:157966 (r)	2E+05	##	long tail fiber proximal subunit	QAY00486.1	0.0 (99.64)	100		Escherichia phage Ecwhy_1
Gp318c	158086:158985 (r)	31524	299	virion structural protein	WGM49548.1	0.0 (97.66)	100		Escherichia phage vB_Ec-M-J
Gp319c	159000:161141 (r)	77649	713	colanic acid biosynthesis protein	QXN76294.1	0.0 (99.30)	100		Escherichia phage BF17
Gp320c	161156:162283 (r)	41244	375	structural protein	QAY00483.1	0.0 (99.47)	100		Escherichia phage Ecwhy_1
Gp321	162393:162890 (f)	18828	165	hypothetical protein	WOL22757.1	6e-114(96.36)	100		Escherichia phage vB_EcoM_JNE01
Gp322	162900:163508 (f)	21937	202	Gp105; bacteriophage baseplate protein, viral protein; HET: MSE; 2.38A	6HHK_A			0.00036(97.97)	Listeria phage A511
Gp323	163521:164621 (f)	40375	366	structural protein	WAE77168.1	0.0 (89.34)	100		Escherichia phage ph0011
Gp324c	164655:165119 (r)	17640	154	nudix hydrolase	QXN76299.1	2e-110 (100.00)	100		Escherichia phage BF17
Gp325c	165132:166475 (r)	51482	447	tail sheath stabilizer and completion protein	UTS53895.1	0.0 (88.14)	100		Escherichia phage UE-S1
Gp326c	166472:167968 (r)	57212	498	hypothetical protein	QXN76301.1	0.0 (99.60)	100		Escherichia phage BF17
Gp327	168077:170365 (f)	83473	762	ATP-dependent Clp protease ATP-binding subunit	WP_210765405.1	0.0 (100.00)	100		Escherichia coli

Gp328c	170399:180370 (r)	4E+05	##	hypothetical protein Ecwhy1_196	QAY00475.1	0.0 (99.76)	100		Escherichia phage Ecwhy_1
Gp329c	180451:183912 (r)	1E+05	##	baseplate wedge subunit	YP_009150851.1	0.0 (92.28)	100		Escherichia phage PBECO4
Gp330c	184015:184410 (r)	15113	131	Baseplate wedge protein gp25; contractile sheath, baseplate, wedge, sheath polymerization, viral protein; HET: MSE;	5IW9_B			1.3e-17(99.81)	Tequatrovirus T4
Gp331c	184426:184914 (r)	18634	162	putative baseplate hub subunit and tail lysozyme	QBO61827.1	2e-105 (90.74)	100		Escherichia phage vB_EcoM_G17
Gp332c	184952:187615 (r)	95555	887	baseplate hub subunit and tail lysozyme	YP_009150848.1	0.0 (92.90)	100		Escherichia phage PBECO4
Gp333c	187629:189884 (r)	85482	751	virion structural protein	WGM49564.1	0.0 (99.07)	100		Escherichia phage vB_Ec-M-J
Gp334c	189896:190210 (r)	12330	104	baseplate wedge protein	WAE77179.1	2e-65 (91.35)	100		Escherichia phage ph0011
Gp335c	190210:190941 (r)	27471	243	Baseplate tail-tube protein gp48; T4, baseplate-tail tube complex, pre-attachment, bacteriophage, bacterial virus, hexag	5IV5_DH			0.42(95.56)	Tequatrovirus T4
Gp336	190974:191912 (f)	36856	312	putative RNA polymerase sigma factor	UTS53906.1	0.0 (91.23)	100		Escherichia phage UE-S1
Gp337	191899:192930 (f)	39901	343	recombination endonuclease subunit	UTS53907.1	0.0 (95.34)	100		Escherichia phage UE-S1
Gp338	192943:195090 (f)	81664	715	SbcC-like subunit of palindrome specific endonuclease	WGM49569.1	0.0 (99.86)	100		Escherichia phage vB_Ec-M-J
Gp339c	195109:195600 (r)	18877	163	endonuclease VII	WGM49570.1	8e-116 (99.39)	100		Escherichia phage vB_Ec-M-J
Gp340c	195980:196192 (r)	7895	70	hypothetical protein	WPK18801.1	3e-14 (97.06)	48		Salmonella phage SD-2_S15
Gp341c	196248:196367 (r)	4606	39	hypothetical protein	WPK18799.1	2e-06 (77.42)	79		Salmonella phage SD-2_S15
Gp342c	196440:196649 (r)	8084	69	hypothetical protein Ecwhy1_184	QAY00464.1	2e-41 (100.00)	100		Escherichia phage Ecwhy_1
Gp343c	196649:197485 (r)	31407	278	baseplate hub subunit	QAY00463.1	0.0 (100.00)	100		Escherichia phage Ecwhy_1

Gp344	197631:199829 (f)	79146	732	Pore-forming tail tip protein pb2; Bacteriophage, Siphophage, T5, baseplate, viral protein; 3.53A	7ZHJ_f			0.053(97.77)	Escherichia phage T5
Gp345	199842:201578 (f)	64471	578	portal protein	QXN76318.1	0.0 (99.83)	100		Escherichia phage BF17
Gp346	201652:201882 (f)	8651	76	hypothetical protein vBEcoMphAPEC6_01665	WAE77190.1	4e-44 (96.05)	100		Escherichia phage ph0011
Gp347	201983:202816 (f)	30563	277	structural protein	QAY00459.1	0.0 (100.00)	100		Escherichia phage Ecwhy_1
Gp348	202848:203468 (f)	22746	206	Prohead core protein protease; protease pentamer, phage T4, prohead, hydrolase; 1.943A	5JBL_C			1.3e-36(100)	Enterobacteria phage T4
Gp349	203535:204650 (f)	42324	371	Outer capsid protein sigma-1	6GAP_B			5.5(94.56)	Mammalian orthoreovirus 3 Dearing
Gp350	204741:205916 (f)	42262	391	major capsid protein	WEM33315.1	0.0 (91.56)	100		Escherichia phage EJP2
Gp351	206027:206377 (f)	13704	116	hypothetical protein vBEcoMphAPEC6_01695	WAE77196.1	2e-68 (88.70)	99		Escherichia phage ph0011
Gp352	206417:206725 (f)	12096	102	GIY-YIG nuclease family protein	YP_009150830.1	5e-65 (92.16)	100		Escherichia phage PBECO4
Gp353	206767:208395 (f)	56915	542	tail fiber	QAY00452.1	0.0 (99.82)	100		Escherichia phage Ecwhy_1
Gp354	208397:208915 (f)	19833	172	tail fiber assembly protein	QXN76328.1	2e-121 (100.00)	100		Escherichia phage BF17
Gp355	209016:209321 (f)	11414	101	hypothetical protein ACQ29_gp513	YP_009150827.1	6e-59 (92.93)	100		Escherichia phage PBECO4
Gp356	209404:212409 (f)	1E+05	##	DNA polymerase	QAY00449.1	0.0 (100.00)	100		Escherichia phage Ecwhy_1
Gp357	212420:212560 (f)	5255	46	hypothetical protein Ecwhy1_168	QAY00448.1	2e-22 (97.83)	100		Escherichia phage Ecwhy_1
Gp358	212562:212864 (f)	11358	100	hypothetical protein	QXN76332.1	3e-64(99.00)	100		Escherichia phage BF17
Gp359	212969:213700 (f)	26500	243	protein (sliding clamp); sliding clamp, GP45, replisome	1B77_B			1e-31(100)	Enterobacteria phage RB69
Gp360	213768:213974 (f)	7902	68	CxxC-CXXC-SSSS domain-containing protein	WAE77205.1	3e-42 (95.59)	100		Escherichia phage ph0011
Gp361	213976:214278 (f)	10757	100	co-chaperone GroES	WGM49591.1	2e-64 (99.00)	100		Escherichia phage vB_Ec-M-J
Gp362	214271:214996 (f)	28321	241	virion structural protein	WGM49592.1	4e-173 (99.59)	100		Escherichia phage vB_Ec-M-J

Gp363c	215041:215730 (r)	24308	229	virion structural protein	WGM49593.1	2e-159 (99.13)	100		Escherichia phage vB_Ec-M-J
Gp364c	215746:216345 (r)	20755	199	virion structural protein	WGM49594.1	3e-138 (98.99)	100		Escherichia phage vB_Ec-M-J
Gp365	216557:217543 (f)	38066	328	RNase H-like 5'-3' exonuclease	YP_009101595.1	0.0 (93.27)	100		Escherichia phage 121Q
Gp366	217545:218030 (f)	18514	161	hypothetical protein	QXN76340.1	7e-113 (99.38)	100		Escherichia phage BF17
Gp367	218074:218760 (f)	26375	228	DNA packaging protein Gp17; nucleotide-binding fold, hydrolase; HET: ADP; 1.8A	2O0J_A			1.7e-22(99.91)	Enterobacteria phage T4
Gp368	218735:219076 (f)	13454	113	terminase large subunit	QXN76342.1	2e-77 (100.00)	100		Escherichia phage BF17
Gp369	219039:220376 (f)	50874	445	large terminase subunit	USL83697.1	0.0 (94.38)	100		Escherichia phage A4
Gp370	220409:221713 (f)	48942	434	structural protein	WAE77215.1	0.0 (91.71)	100		Escherichia phage ph0011
Gp371	221887:222936 (f)	38920	349	gp32 single stranded DNA binding protein	2A1K_A			1.3e-29(99.97)	Escherichia phage RB69
Gp372	223006:224124 (f)	41616	372	protein RecA	WAE77217.1	0.0 (95.43)	100		Escherichia phage ph0011
Gp373	224124:224585 (f)	18134	153	recombination, repair and ssDNA binding protein	WAE77218.1	5e-99 (94.12)	100		Escherichia phage ph0011
Gp374	224633:225451 (f)	31767	272	exodeoxyribonuclease X	QXN76348.1	0.0 (98.53)	100		Escherichia phage BF17
Gp375	225467:226951 (f)	55252	494	ATP-dependent DNA helicase	QXN76349.1	0.0 (99.80)	100		Escherichia phage BF17
Gp376	227008:227436 (f)	16501	142	hypothetical protein vBEcoMphAPEC6_01820	WAE77221.1	8e-79 (96.77)	100		Escherichia phage ph0011
Gp377	227446:227943 (f)	18892	165	hypothetical protein vBEcoMphAPEC6_01825	WAE77222.1	9e-113 (95.15)	100		Escherichia phage ph0011
Gp378	227946:228791 (f)	32626	281	hypothetical protein EcMJ_366	WGM49608.1	0.0 (99.29)	100		Escherichia phage vB_Ec-M-J
Gp379	228772:229482 (f)	28112	236	zinc-finger domain protein	WGM49609.1	2e-171 (99.58)	100		Escherichia phage vB_Ec-M-J
Gp380	229479:229808 (f)	12442	109	Tail needle protein gp26; Viral protein, P22, Tail Needle, Membrane penetration; 2.75A	4ZXQ_A			0.42(95.21)	Enterobacteria phage P22
Gp381	229786:230424 (f)	24339	212	DNA polymerase	WGM49611.1	2e-156 (99.53)	100		Escherichia phage vB_Ec-M-J
Gp382	230455:230667 (f)	7990	70	hypothetical protein	WPK20084.1	2e-25 (74.29)	100		Salmonella phage SD-1_S14

Gp383	230603:231799 (f)	45704	398	virion structural protein	YP_009150800.1	0.0 (91.52)	82		Escherichia phage PBECO4
Gp384	231855:232910 (f)	40123	351	DNA primase subunit	UTS53377.1	0.0 (90.03)	100		Escherichia phage UE-S1
Gp385	232937:234457 (f)	56747	506	helicase	QXN76359.1	0.0 (99.80)	100		Escherichia phage BF17
Gp386	234461:234733 (f)	10413	90	hypothetical protein UES1_004	UTS53379.1	3e-55 (97.78)	100		Escherichia phage UE-S1
Gp387	234735:234902 (f)	5974	55	hypothetical protein EcMJ_374	WGM49616.1	1e-30 (100.00)	100		Escherichia phage vB_Ec-M-J
Gp388	234899:235291 (f)	14495	130	hypothetical protein Ecwhy1_137	QAY00418.1	2e-89 (100.00)	100		Escherichia phage Ecwhy_1
Gp389	235349:236311 (f)	35559	320	structural protein	QAY00417.1	0.0 (99.69)	100		Escherichia phage Ecwhy_1
Gp390	236311:236529 (f)	8282	72	Penicillinase repressor; protein-dna complex, repressor, monomer, operator, antibiotics, transcription regulator; NMR		2P7C_B		0.59(93.78)	Escherichia coli
Gp391	236526:237203 (f)	24977	225	hypothetical protein UES1_009	UTS53384.1	2e-142 (87.05%)	99		Escherichia phage UE-S1
Gp392	237237:237869 (f)	23393	210	hypothetical protein vBEcoMphAPEC6_01905	WAE77238.1	4e-136 (91.35)	100		Escherichia phage ph0011
Gp393	237886:238215 (f)	12049	109	hypothetical protein vBEcoMphAPEC6_01910	WAE77239.1	1e-63 (86.24)	100		Escherichia phage ph0011
Gp394	238262:238981 (f)	27495	239	exonuclease	QAY00412.1	3e-178 (100.00)	100		Escherichia phage Ecwhy_1
Gp395	238968:239525 (f)	20904	185	hypothetical protein	QXN76369.1	1e-119 (99.46)	100		Escherichia phage BF17
Gp396	239554:240135 (f)	21401	193	hyaluronidase, phage associated; lyase, hyaluronan lyase, phage tail fibre, triple-stranded beta- helix, hyaluronidase		2C3F_A		0.031(94.24)	Escherichia coli BL21
Gp397	240202:242520 (f)	87588	772	ribonucleotide reductase large subunit	YP_009150786.1	0.0 (97.41)	100		Escherichia phage PBECO4
Gp398	242566:243339 (f)	30365	257	hypothetical protein Ecwhy1_127	QAY00408.1	0.0 (100.00)	100		Escherichia phage Ecwhy_1
Gp399	243352:244476 (f)	42909	374	ribonucleotide-diphosphate reductase subunit beta	YP_009150785.1	0.0 (96.52)	100		Escherichia phage PBECO4

Gp400	244525:244692 (f)	6036	55	hypothetical protein Ecwhy1_125	QAY00406.1	5e-31(100)	100		Escherichia phage Ecwhy_1
Gp401	244755:244988 (f)	8936	77	hypothetical protein ACQ29_gp469	YP_009150783.1	6e-43 93.51%	100		Escherichia phage PBECO4
Gp402	245003:245398 (f)	15145	131	hypothetical protein Ecwhy1_123	QAY00404.1	7e-93 (100.00)	100		Escherichia phage Ecwhy_1
Gp403	245398:246132 (f)	27327	244	nucleotide pyrophosphohydrolase	QAY00403.1	9e-177 (99.59)	100		Escherichia phage Ecwhy_1
Gp404c	246175:246513 (r)	13434	112	hypothetical protein Ecwhy1_121	QAY00402.1	2e-76 (100.00)	100		Escherichia phage Ecwhy_1
Gp405c	246536:246835 (r)	11464	99	hypothetical protein G17_00268	QBO61757.1	1e-54 (88.89)	100		Escherichia phage vB_EcoM_G17
Gp406c	246852:247514 (r)	25195	220	Holin; phage, lysis inhibition, viral protein; 1.65A	6PX4_B			2.6(89.89)	Escherichia phage ECML-134
Gp407	247666:248034 (f)	13938	122	hypothetical protein Ecwhy1_118	QAY00399.1	2e-80 (100.00)	100		Escherichia phage Ecwhy_1
Gp408	248022:248543 (f)	20018	173	dihydrofolate reductase	QAY00398.1	1e-126 (100.00)	100		Escherichia phage Ecwhy_1
Gp409	248540:248974 (f)	16120	144	RNaseH	QAY00397.1	2e-100 (100.00)	100		Escherichia phage Ecwhy_1
Gp410	249000:250340 (f)	51233	446	ATP-dependent DNA helicase	QXN76384.1	0.0 (100.00)	100		Escherichia phage BF17
Gp411	250424:250990 (f)	20660	188	hypothetical protein	QXN76385.1	1e-129 (99.47)	100		Escherichia phage BF17
Gp412	251014:251208 (f)	7421	64	hypothetical protein vBEcoMphAPEC6_02000	WAE77257.1	2e-38 (98.44)	100		Escherichia phage ph0011
Gp413	251278:251781 (f)	18356	167	translation initiation factor IF3	QAY00393.1	1e-114 (99.40)	100		Escherichia phage Ecwhy_1
Gp414	251795:252340 (f)	20578	181	protease adapter protein	QAY00392.1	3e-131 (100.00)	100		Escherichia phage Ecwhy_1
Gp415	252415:252960 (f)	20959	181	KTSC domain-containing protein	UTS53410.1	7e-126 (95.03)	100		Escherichia phage UE-S1
Gp416	252950:254521 (f)	59498	523	virion structural protein	WGM49644.1	0.0 (99.81)	100		Escherichia phage vB_Ec-M-J
Gp417	254521:255315 (f)	29195	264	structural protein	QAY00389.1	0.0 (100.00)	100		Escherichia phage Ecwhy_1
Gp418	255415:257379 (f)	74290	654	topoisomerase II large subunit	QAY00388.1	0.0 (99.85)	100		Escherichia phage Ecwhy_1
Gp419	257430:258842 (f)	53493	470	topoisomerase II medium subunit	QAY00387.1	0.0(100)	100		Escherichia phage Ecwhy_1
Gp420	258931:261327 (f)	89977	798	virion structural protein	WGM49648.1	0.0(100)	100		Escherichia phage vB_Ec-M-J
Gp421	261600:262118 (f)	20702	172	DNA-pol3-alpha domain-containing protein	WAE77267.1	8e-110 (93.02)	100		Escherichia phage ph0011
Gp422	262182:262526 (f)	13187	114	hypothetical protein Ecwhy1_102	QAY00383.1	4e-78 (99.12)	100		Escherichia phage Ecwhy_1

Gp423	262530:262856 (f)	11990	108	co-chaperone GroES	WAE77269.1	6e-64 (88.89)	100		Escherichia phage ph0011
Gp424	262913:263182 (f)	10395	89	hypothetical protein vBEcoMphAPEC6_02065	WAE77270.1	3e-49 (86.36)	100		Escherichia phage ph0011
Gp425	263209:264162 (f)	36090	317	clamp loader subunit	QAY00380.1	0.0 (100.00)	100		Escherichia phage Ecwhy_1
Gp426	264162:264788 (f)	24644	208	hypothetical protein UES1_047	UTS53422.1	8e-109(72.73)	100		Escherichia phage UE-S1
Gp427	264888:265454 (f)	21413	188	hypothetical protein	QXN76402.1	2e-134 (98.94)	100		Escherichia phage BF17
Gp428	265451:266131 (f)	25384	226	hypothetical protein vBEcoMphAPEC6_02085	WAE77274.1	2e-150 (89.38)	100		Escherichia phage ph0011
Gp429	266143:266946 (f)	32004	267	hypothetical protein Ecwhy1_95	QAY00376.1	0.0 (100.00)	100		Escherichia phage Ecwhy_1
Gp430	267046:268173 (f)	43420	375	hypothetical protein Ecwhy1_94	QAY00375.1	0.0 (99.73)	100		Escherichia phage Ecwhy_1
Gp431	268257:269399 (f)	42678	380	toxic anion resistance protein	UTS53427.1	0.0 (87.70)	100		Escherichia phage UE-S1
Gp432	269481:270170 (f)	25932	229	peptidase	QXN76407.1	5e-170 (99.56)	100		Escherichia phage BF17
Gp433	270174:271268 (f)	42462	364	hypothetical protein EcMJ_420	WGM49662.1	0.0 (99.73)	100		Escherichia phage vB_Ec-M-J
Gp434	271265:271819 (f)	21040	184	LEMA protein	WAE77280.1	1e-121 (90.22)	100		Escherichia phage ph0011
Gp435	271867:272454 (f)	21825	195	hypothetical protein	QXN76410.1	2e-140 (98.97)	100		Escherichia phage BF17
Gp436	272438:273022 (f)	22592	194	hypothetical protein Ecwhy1_88	QAY00369.1	7e-141 (100.00)	100		Escherichia phage Ecwhy_1
Gp437	273025:273378 (f)	13279	117	hypothetical protein	QXN76412.1	5e-79 (100.00)	100		Escherichia phage BF17]
Gp438	273381:273872 (f)	19238	163	hypothetical protein EcMJ_425	WGM49667.1	2e-116(98.77)	100		Escherichia phage vB_Ec-M-J
Gp439	273865:274143 (f)	10621	92	DNA primase/helicase; zinc-biding domain, TOPRIM fold, DNA replication, DNA-directed RNA polymerase, Primosome, Late pro	1NUI_A			8.6(62.61)	Escherichia phage T7
Gp440	274124:274360 (f)	9102	78	hypothetical protein Ecwhy1_84	QAY00365.1	2e-50 (100.00)	100		Escherichia phage Ecwhy_1
Gp441	274368:274601 (f)	8637	77	hypothetical protein Ecwhy1_83	QAY00364.1	9e-43 (97.37)	98		Escherichia phage Ecwhy_1
Gp442	274598:275020 (f)	16617	140	hypothetical protein vBEcoMphAPEC6_02155	WAE77288.1	8e-91 (92.14)	100		Escherichia phage ph0011
Gp443	275017:275352 (f)	13116	111	virion structural protein	WGM49672.1	9e-75 (99.10)	100		Escherichia phage vB_Ec-M-J

Gp444	275349:275543 (f)	7516	64	hypothetical protein Ecwhy1_80	QAY00361.1	4e-39 (100.00)	100		Escherichia phage Ecwhy_1
Gp445	275547:275975 (f)	16098	142	hypothetical protein UES1_066	UTS53441.1	2e-87 (89.44)	100		Escherichia phage UE-S1
Gp446	276032:276583 (f)	20668	183	hypothetical protein vBEcoMphAPEC6_02175	WAE77292.1	1e-101 (75.96)	100		Escherichia phage ph0011
Gp447	276741:277136 (f)	15803	131	hypothetical protein Ecwhy1_77	QAY00358.1	2e-87 (96.95)	100		Escherichia phage Ecwhy_1
Gp448	277192:277602 (f)	15831	136	hypothetical protein vBEcoMphAPEC6_02185	WAE77294.1	2e-68 (73.33)	99		Escherichia phage ph0011
Gp449	277604:278008 (f)	15498	134	hypothetical protein Ecwhy1_75	QAY00356.1	4e-93 (99.25)	100		Escherichia phage Ecwhy_1
Gp450	278010:278384 (f)	14195	124	hypothetical protein	QXN76425.1	1e-80 (97.58)	100		Escherichia phage BF17
Gp451	278384:278836 (f)	17328	150	hypothetical protein EcMJ_438	WGM49680.1	1e-101 (100.00)	100		Escherichia phage vB_Ec-M-J
Gp452	278830:279285 (f)	17524	151	hypothetical protein	QXN76427.1	3e-104 (99.34)	100		Escherichia phage BF17
Gp453	279300:279560 (f)	10398	86	hypothetical protein	WOL22888.1	7e-53 (96.51)	100		Escherichia phage vB_EcoM_JNE01
Gp454	279603:279890 (f)	10882	95	hypothetical protein Ecwhy1_70	QAY00351.1	1e-63 (100)	100		Escherichia phage Ecwhy_1
Gp455	279943:280803 (f)	33186	286	DNA methyltransferase	WGM49685.1	0.0 (100.00)	100		Escherichia phage vB_Ec-M-J
Gp456	280755:281285 (f)	20729	176	hypothetical protein Ecwhy1_68	QAY00349.1	6e-127(100)	100		Escherichia phage Ecwhy_1
Gp457	281328:281843 (f)	20079	171	hypothetical protein Ecwhy1_67	QAY00348.1	3e-119 (98.83)	100		Escherichia phage Ecwhy_1
Gp458	281840:282295 (f)	17566	151	hypothetical protein EcMJ_446	WGM49688.1	2e-104 (98.68)	100		Escherichia phage vB_Ec-M-J
Gp459	282295:282678 (f)	14960	127	hypothetical protein Ecwhy1_65	QAY00346.1	1e-89 (99.21)	100		Escherichia phage Ecwhy_1
Gp460	282675:283118 (f)	17643	147	hypothetical protein EcMJ_448	WGM49690.1	2e-104 (99.32)	100		Escherichia phage vB_Ec-M-J
Gp461	283121:283264 (f)	5400	47	hypothetical protein vBEcoMphAPEC6_02250	WAE77307.1	2e-24 (93.62)	100		Escherichia phage ph0011
Gp462	283281:283451 (f)	6613	56	hypothetical protein UES1_083	UTS53458.1	3e-29 (91.07)	100		Escherichia phage UE-S1
Gp463	283451:283594 (f)	5309	47	hypothetical protein	QXN76439.1	9e-25 (100.00)	100		Escherichia phage BF17
Gp464	283652:284224 (f)	22201	190	hypothetical protein ACQ29_gp407	YP_009150721.1	4e-136 (97.37)	100		Escherichia phage PBECO4
Gp465	284211:284408 (f)	7552	65	hypothetical protein Ecwhy1_59	QAY00340.1	3e-38 (96.92)	100		Escherichia phage Ecwhy_1
Gp466	284411:285241 (f)	32206	276	hypothetical protein	QXN76442.1	0.0 (99.28)	100		Escherichia phage BF17

Gp467	285238:285528 (f)	10942	96	hypothetical protein vBEcoMphAPEC6_02280	WAE77313.1	3e-59 (93.75)	100		Escherichia phage ph0011
Gp468	285528:285797 (f)	10550	89	Scaffolding domain delta; Prohead I, icosahedral symmetry, HK97, phage, capsid, virus; 3.5A	8FQK_F			3.6(88.4)	Escherichia phage HK97
Gp469	285790:286086 (f)	11487	98	hypothetical protein	QXN76445.1	3e-64 (100.00)	100		Escherichia phage BF17
Gp470	286204:286413 (f)	7995	69	hypothetical protein vBEcoMphAPEC6_02295	WAE77316.1	4e-40 (92.75%)	100		Escherichia phage ph0011
Gp471	286479:286985 (f)	19061	168	hypothetical protein	QXN76447.1	2e-117 (99.40)	100		Escherichia phage BF17
Gp472	286985:287290 (f)	11345	101	hypothetical protein	QXN76448.1	4e-64 (99.01)	100		Escherichia phage BF17
Gp473	287313:287462 (f)	5454	49	hypothetical protein	QXN76449.1	9e-25(100.00)	100		Escherichia phage BF17
Gp474	287513:287905 (f)	15314	130	hypothetical protein Ecwhy1_50	QAY00331.1	1e-89 (96.92)	100		Escherichia phage Ecwhy_1
Gp475	287953:288522 (f)	21770	189	hypothetical protein UES1_097	UTS53472.1	1e-131 (97.88)	100		Escherichia phage UE-S1
Gp476	288524:289012 (f)	19078	162	hypothetical protein Ecwhy1_48	QAY00329.1	1e-113 (98.77)	100		Escherichia phage Ecwhy_1
Gp477	289009:289413 (f)	16111	134	hypothetical protein ACQ29_gp394	YP_009150708.1	5e-91 (98.51)	100		Escherichia phage PBECO4
Gp478	289403:289588 (f)	7469	61	hypothetical protein Ecwhy1_46	QAY00327.1	1e-36 (100.00)	100		Escherichia phage Ecwhy_1
Gp479	289585:289755 (f)	6912	56	hypothetical protein	QXN76455.1	5e-33 (100.00)	100		Escherichia phage BF17
Gp480	289766:289978 (f)	8044	70	hypothetical protein Ecwhy1_42	QAY00323.1	5e-40 (95.71)	100		Escherichia phage Ecwhy_1
Gp481	290128:290325 (f)	7360	65	hypothetical protein vBEcoMphAPEC6_02355	WAE77328.1	6e-33 (93.44)	100		Escherichia phage ph0011
Gp482	290336:290554 (f)	7750	72	hypothetical protein G17_00188	QBO61678.1	8e-29 (76.81)	95		Escherichia phage vB_EcoM_G17
Gp483	290576:290848 (f)	10834	90	hypothetical protein Ecwhy1_39	QAY00321.1	3e-59 (98.89)	100		Escherichia phage Ecwhy_1
Gp484	291089:291253 (f)	6731	54	hypothetical protein	QXN76460.1	8e-31 (98.15)	100		Escherichia phage BF17
Gp485	291250:291414 (f)	6762	54	hypothetical protein Ecwhy1_37	QAY00319.1	3e-31 (100.00)	100		Escherichia phage Ecwhy_1
Gp486	291411:291575 (f)	6823	54	hypothetical protein UES1_109	UTS53481.1	5e-31 (98.15)	100		Escherichia phage UE-S1
Gp487	291646:292011 (f)	14503	121	hypothetical protein	QXN76463.1	4e-80 (96.69)	100		Escherichia phage BF17
Gp488	292186:292392 (f)	7752	68	hypothetical protein	WPK20988.1	4e-42 (98.53)	100		Salmonella phage SD-15_S21

Gp489	292470:292841 (f)	14420	123	hypothetical protein Ecwhy1_34	QAY00316.1	2e-82 (100.00)	100		Escherichia phage Ecwhy_1
Gp490	293303:293488 (f)	6926	61	hypothetical protein Ecwhy1_33	QAY00315.1	2e-31 (100.00)	100		Escherichia phage Ecwhy_1
Gp491	293498:293755 (f)	9998	85	hypothetical protein PBI_121Q_134	YP_009101722.1	8e-53 (97.65)	100		Escherichia phage 121Q
Gp492	293764:294165 (f)	15775	133	hypothetical protein	AXC36639.1	4e-86 (93.23)	100		Escherichia phage UB
Gp493	294178:294393 (f)	7800	71	hypothetical protein	AXC36638.1	6e-42 (95.77)	100		Escherichia phage UB
Gp494	294595:294810 (f)	8015	71	hypothetical protein Ecwhy1_30	QAY00312.1	8e-44 (100.00)	100		Escherichia phage Ecwhy_1
Gp495	294797:294910 (f)	4174	37	hypothetical protein PBI_121Q_139	YP_009101727.1	1e-16 (100.00)	100		Escherichia phage 121Q
Gp496	295158:295574 (f)	16362	138	hypothetical protein vBEcoMphAPEC6_02450	WAE77340.1	2e-85 (91.30)	100		Escherichia phage ph0011
Gp497	295567:295926 (f)	13822	119	hypothetical protein EcMJ_482	WGM49724.1	2e-79 (98.32)	100		Escherichia phage vB_Ec-M-J
Gp498	295940:296131 (f)	7503	63	hypothetical protein Ecwhy1_26	QAY00308.1	2e-37 (96.83)	100		Escherichia phage Ecwhy_1
Gp499	296131:296415 (f)	11000	94	hypothetical protein Ecwhy1_25	QAY00307.1	6e-63 (98.94)	100		Escherichia phage Ecwhy_1
Gp500	296655:297071 (f)	16342	138	hypothetical protein	QXN76473.1	4e-94 (99.28)	100		Escherichia phage BF17
Gp501	297144:297551 (f)	15490	135	hypothetical protein	QXN76474.1	1e-92 (100.00)	100		Escherichia phage BF17
Gp502	297541:297987 (f)	16767	148	hypothetical protein Ecwhy1_22	QAY00304.1	3e-104 (99.32)	100		Escherichia phage Ecwhy_1
Gp503	297999:298418 (f)	15938	139	hypothetical protein EcMJ_488	WGM49730.1	2e-95 (99.28)	100		Escherichia phage vB_Ec-M-J
Gp504	298415:298780 (f)	14209	121	hypothetical protein G17_00162	QBO61658.1	3e-69 (84.17)	100		Escherichia phage vB_EcoM_G17
Gp505	298935:299276 (f)	13112	113	hypothetical protein	QXN76479.1	9e-78 (99.12)	100		Escherichia phage BF17
Gp506	299288:300103 (f)	30576	271	hypothetical protein	QXN76480.1	0.0 (99.63)	100		Escherichia phage BF17
Gp507c	300148:301305 (r)	42801	385	structural protein	QAY00298.1	0.0 (99.48)	100		Escherichia phage Ecwhy_1
Gp508	301422:302006 (f)	22009	194	Restriction endonuclease (Eco15I)	8Q5O_B			0.00031(97.79)	Escherichia coli BL21(DE3)
Gp509	302003:302230 (f)	8440	75	hypothetical protein Ecwhy1_14	QAY00296.1	1e-45 (100.00)	100		Escherichia phage Ecwhy_1
Gp510	302233:302691 (f)	18128	152	hypothetical protein	AXC36622.1	9e-94 (87.42)	100		Escherichia phage UB
Gp511	302688:303131 (f)	17349	147	hypothetical protein EcMJ_497	WGM49739.1	4e-101 (99.32)	100		Escherichia phage vB_Ec-M-J
Gp512	303185:303643 (f)	17247	152	hypothetical protein EO157G_1440	BBM61733.1	2e-91 (87.50)	100		Escherichia phage SP27

Gp513	303643:304065 (f)	16476	140	membrane protein	WGM49741.1	1e-95 (98.57)	100		Escherichia phage vB_Ec-M-J
Gp514	304062:304487 (f)	15825	141	hypothetical protein vBEcoMphAPEC6_02540	WAE77358.1	4e-90 (88.65)	100		Escherichia phage ph0011
Gp515	304547:304798 (f)	9674	83	hypothetical protein Ecwhy1_8	QAY00290.1	5e-50 (100.00)	100		Escherichia phage Ecwhy_1
Gp516	304873:305133 (f)	9545	86	hypothetical protein Ecwhy1_7	QAY00289.1	4e-52 (98.84)	100		Escherichia phage Ecwhy_1
Gp517	305139:305558 (f)	16965	139	hypothetical protein EcMJ_503	WGM49745.1	2e-93 (98.56)	100		Escherichia phage vB_Ec-M-J
Gp518	305560:306057 (f)	19105	165	ATP-dependent protease subunit HslV; Threonine prtease, HslUV, bacterial proteasome, hydrolase; HET: SO4; 2.0A	6KR1			3.1e-11 (99.39)	Escherichia coli BL21(DE3)
Gp519	306080:307048 (f)	36896	322	RNA polymerase sigma factor	QXN76493.1	0.0 (99.38)	100		Escherichia phage BF17
Gp520	307710:307919 (f)	7711	69	hypothetical protein vBEcoMphAPEC6_02570	WAE77364.1	5e-29 (75.36)	100		Escherichia phage ph0011
Gp521	307916:308143 (f)	8598	75	hypothetical protein ACQ29_gp354	YP_009150668.1	2e-36 (87.14)	100		Escherichia phage PBECO4
Gp522	308145:308939 (f)	31062	264	HNH endonuclease	WGM49750.1	0.0 (98.48)	100		Escherichia phage vB_Ec-M-J
Gp523	308954:309136 (f)	6835	60	hypothetical protein vBEcoMphAPEC6_02585	WAE77367.1	1e-34 (96.67)	100		Escherichia phage ph0011
Gp524	309146:309346 (f)	7406	66	hypothetical protein vBEcoMphAPEC6_gp061c	QDF13691.1	3e-38 (95.45)	100		Escherichia phage vB_EcoM_phAPEC6
Gp525	309564:309872 (f)	11356	102	hypothetical protein ACQ29_gp349	YP_009150663.1	4e-70 (99.02)	100		Escherichia phage PBECO4
Gp526	309872:310123 (f)	9029	83	Lytic conversion lipoprotein; Outer membrane TonB-dependent transporter FhuA Bacteriophage T5 Superinfection exclusion E	8A60_B			0.000057(97.82)	Escherichia phage T5
Gp527	310120:310488 (f)	14519	122	hypothetical protein Ecwhy1_585	QAY00856.1	1e-84 (99.18)	100		Escherichia phage Ecwhy_1

Gp528	310485:310760 (f)	10567	91	Lytic conversion lipoprotein; Outer membrane TonB-dependent transporter FhuA Bacteriophage T5 Superinfection exclusion E	8A60_B			0.0075(96.04)	Escherichia phage T5
Gp529	310760:310975 (f)	8042	71	hypothetical protein vBEcoMphAPEC6_ 02615	WAE77373. 1	3e-42 (97.18)	100		Escherichia phage ph0011
Gp530	310972:311118 (f)	5553	48	hypothetical protein ACQ29_gp344	YP_0091506 58.1	3e-24 (95.83)	100		Escherichia phage PBECO4
Gp531	311129:311329 (f)	7503	66	hypothetical protein Ecwhy1_581	QAY00853. 1	6e-41 (98.48)	100		Escherichia phage Ecwhy_1
Gp532	311329:311844 (f)	19981	171	hypothetical protein EcMJ_519	WGM49761 .1	4e-121 (99.42)	100		Escherichia phage vB_Ec-M-J
Gp533	311841:312062 (f)	8763	73	hypothetical protein vBEcoMphAPEC6_ 02630	WAE77376. 1	8e-43 (91.78)	100		Escherichia phage ph0011
Gp534	312066:312284 (f)	8507	72	hypothetical protein vBEcoMphAPEC6_ 02635	WAE77377. 1	1e-34 (83.33)	100		Escherichia phage ph0011
Gp535	312277:312492 (f)	8472	71	hypothetical protein Ecwhy1_577	QAY00849. 1	3e-42 (95.71)	100		Escherichia phage Ecwhy_1
Gp536	312526:313188 (f)	25383	220	hypothetical protein Ecwhy1_576	QAY00848. 1	5e-162 (98.64)	100		Escherichia phage Ecwhy_1
Gp537	313179:313421 (f)	9056	80	hypothetical protein	QXN76512. 1	1e-44 (100.00)	100		Escherichia phage BF17
Gp538	313480:313806 (f)	12471	108	hypothetical protein EcMJ_525	WGM49767 .1	7e-65 (98.15)	100		Escherichia phage vB_Ec-M-J
Gp539	313885:314250 (f)	13843	121	hypothetical protein	WOL22973. 1	1e-55 (71.43)	100		Escherichia phage vB_EcoM_JNE01
Gp540	314261:314644 (f)	14226	127	hypothetical protein EcMJ_527	WGM49769 .1	1e-77 (100.00)	100		Escherichia phage vB_Ec-M-J
Gp541	314641:314991 (f)	13142	116	hypothetical protein Ecwhy1_571	QAY00843. 1	3e-80 (100.00)	100		Escherichia phage Ecwhy_1
Gp542	314996:315187 (f)	7671	63	hypothetical protein Ecwhy1_570	QAY00842. 1	2e-38 (100.00)	100		Escherichia phage Ecwhy_1
Gp543	315243:315683 (f)	16898	146	hypothetical protein G17_00121	QBO61617. 1	2e-94 (87.67)	100		Escherichia phage vB_EcoM_G17
Gp544	315676:316020 (f)	13802	114	hypothetical protein Ecwhy1_568	QAY00840. 1	2e-77 (99.12)	100		Escherichia phage Ecwhy_1
Gp545	316035:316301 (f)	10483	88	hypothetical protein Ecwhy1_567	QAY00839. 1	2e-59 (100.00)	100		Escherichia phage Ecwhy_1
Gp546	316313:316606 (f)	11293	97	hypothetical protein	WOL22980. 1	1e-43 (69.79)	100		Escherichia phage vB_EcoM_JNE01
Gp547	316657:316881 (f)	8447	74	hypothetical protein Ecwhy1_565	QAY00837. 1	3e-45 (100.00)	100		Escherichia phage Ecwhy_1
Gp548	316952:317188 (f)	9121	78	hypothetical protein Ecwhy1_564	QAY00836. 1	1e-46 (100.00)	100		Escherichia phage Ecwhy_1

Gp549	317243:317815 (f)	21883	190	hypothetical protein Ecwhy1_563	QAY00835.1	3e-133 (97.89)	100		Escherichia phage Ecwhy_1
Gp550	317829:318272 (f)	16879	147	hypothetical protein Ecwhy1_562	QAY00834.1	1e-103 (99.32)	100		Escherichia phage Ecwhy_1
Gp551	318384:318767 (f)	14768	127	virion structural protein	YP_009101786.1	3e-82 (95.28)	100		Escherichia phage 121Q
Gp552	318855:318968 (f)	3946	37	hypothetical protein	QXN76527.1	9e-17 (100.00)	100		Escherichia phage BF17
Gp553	318977:319240 (f)	9779	87	hypothetical protein	WP_210765684.1	4e-52 (100)	100		Escherichia coli
Gp554	319237:319533 (f)	10921	98	hypothetical protein Ecwhy1_558	QAY00830.1	4e-63 (98.98)	100		Escherichia phage Ecwhy_1
Gp555	319639:321126 (f)	54912	495	virion structural protein	WGM49784.1	0.0 (99.39)	100		Escherichia phage vB_Ec-M-J
Gp556	321198:321449 (f)	9822	83	Lytic conversion lipoprotein; Outer membrane TonB-dependent transporter FhuA Bacteriophage T5 Superinfection exclusion E	8A60_B			0.18(94.52)	Escherichia phage T5
Gp557	321436:321810 (f)	14189	124	hypothetical protein	QXN76532.1	3e-79 (98.39)	100		Escherichia phage BF17
Gp558	321877:322290 (f)	15409	137	hypothetical protein	WOL22988.1	7e-46 (55.97)	97		Escherichia phage vB_EcoM_JNE01
Gp559	322302:322577 (f)	10894	91	hypothetical protein PBI_121Q_206	YP_009101793.1	1e-47 (85.71)	100		Escherichia phage 121Q
Gp560	322587:322925 (f)	13317	112	hypothetical protein	QXN76535.1	3e-75 (99.11)	100		Escherichia phage BF17
Gp561	322935:323276 (f)	12880	113	hypothetical protein	QXN76536.1	8e-72 (99.12)	100		Escherichia phage BF17
Gp562	323278:323622 (f)	13431	114	hypothetical protein EcMJ_549	WGM49791.1	1e-74 (100.00)	100		Escherichia phage vB_Ec-M-J
Gp563	323632:323889 (f)	9625	85	hypothetical protein EcMJ_550	WGM49792.1	1e-55 (98.82)	100		Escherichia phage vB_Ec-M-J
Gp564	323889:324269 (f)	14282	126	hypothetical protein vBEcoMphAPEC6_02745	WAE77399.1	8e-85 (95.24)	100		Escherichia phage ph0011
Gp565	324346:324900 (f)	21703	184	hypothetical protein EcMJ_552	WGM49794.1	3e-134 (98.91)	100		Escherichia phage vB_Ec-M-J
Gp566	324912:325088 (f)	7119	58	hypothetical protein vBEcoMphAPEC6_02755	WAE77401.1	4e-30 (94.83)	100		Escherichia phage ph0011
Gp567	325117:325368 (f)	9473	83	hypothetical protein Ecwhy1_545	QAY00818.1	5e-48 (100.00)	100		Escherichia phage Ecwhy_1
Gp568	325378:325749 (f)	13954	123	hypothetical protein EcMJ_555	WGM49797.1	2e-84 (97.56)	100		Escherichia phage vB_Ec-M-J
Gp569	325751:326020 (f)	10312	89	hypothetical protein PBI_121Q_216	YP_009101803.1	6e-57 (98.88)	100		Escherichia phage 121Q

Gp570	326033:326446 (f)	16076	137	HNH endonuclease	WGM49799.1	2e-91 (97.08)	100		Escherichia phage vB_Ec-M-J
Gp571	326501:327016 (f)	20345	171	endonuclease V N-glycosylase UV repair enzyme	WGM49800.1	9e-121 (96.49)	100		Escherichia phage vB_Ec-M-J
Gp572	327084:327854 (f)	28678	256	hypothetical protein Ecwhy1_537	QAY00810.1	0.0 (99.61)	100		Escherichia phage Ecwhy_1
Gp573	327922:328260 (f)	13056	112	hypothetical protein Ecwhy1_536	QAY00809.1	6e-72 (100.00)	100		Escherichia phage Ecwhy_1
Gp574	328260:328607 (f)	13035	115	hypothetical protein	QXN76553.1	2e-78 (100.00)	100		Escherichia phage BF17
Gp575	328607:328942 (f)	13304	111	hypothetical protein EcMJ_566	WGM49808.1	4e-73 (99.10)	100		Escherichia phage vB_Ec-M-J
Gp576	328942:329256 (f)	12266	104	hypothetical protein	QXN76555.1	1e-68 (100.00)	100		Escherichia phage BF17
Gp577	329256:329624 (f)	14081	122	hypothetical protein Ecwhy1_532	QAY00805.1	5e-82 (100.00)	100		Escherichia phage Ecwhy_1
Gp578	329626:329988 (f)	13712	120	hypothetical protein	QXN76557.1	4e-81 (99.17)	100		Escherichia phage BF17
Gp579	329990:330361 (f)	14113	123	hypothetical protein	QXN76558.1	3e-82 (99.19)	100		Escherichia phage BF17
Gp580c	330398:331522 (r)	40088	374	regulator of chromosome condensation	WGM49813.1	0.0 (99.20)	100		Escherichia phage vB_Ec-M-J
Gp581	331600:332250 (f)	25490	216	hypothetical protein	QXN76560.1	6e-154 (97.69)	100		Escherichia phage BF17
Gp582c	332283:333419 (r)	40442	378	regulator of chromosome condensation	WGM49814.1	0.0 (97.09)	100		Escherichia phage vB_Ec-M-J
Gp583c	333434:334582 (r)	40871	382	regulator of chromosome condensation	WGM49816.1	0.0 (99.21)	100		Escherichia phage vB_Ec-M-J
Gp584c	334660:335790 (r)	40491	376	hypothetical protein	QXN76564.1	0.0 (97.61)	100		QXN76564.1
Gp585c	335870:336979 (r)	39412	369	hypothetical protein	QXN76565.1	0.0 (98.37)	100		Escherichia phage BF17
Gp586c	337052:337789 (r)	23687	245	hypothetical protein UES1_207	UTS53575.1	4e-159 (98.37)	100		Escherichia phage UE-S1
Gp587c	337862:339052 (r)	43521	396	putative DNA condensation protein	WAE77423.1	0.0 (95.20)	100		Escherichia phage ph0011
Gp588c	339124:340266 (r)	40218	380	regulator of chromosome condensation	WGM49819.1	0.0 (97.63)	100		Escherichia phage vB_Ec-M-J
Gp589c	340341:341495 (r)	41614	384	putative DNA condensation protein	QAY00796.1	0.0 (99.22)	100		Escherichia phage Ecwhy_1
Gp590c	341566:342675 (r)	39250	369	putative DNA condensation protein	QAY00793.1	0.0 (99.19)	100		Escherichia phage Ecwhy_1

Gp591c	342684:343877 (r)	43163	397	putative DNA condensation protein	QAY00792.1	0.0 (100.00)	100		Escherichia phage Ecwhy_1
Gp592c	343953:345134 (r)	42436	393	regulator of chromosome condensation	WGM49823.1	0.0 (96.18)	100		Escherichia phage vB_Ec-M-J
Gp593c	345208:346329 (r)	40812	373	regulator of chromosome condensation	WGM49824.1	0.0 (99.46)	100		Escherichia phage vB_Ec-M-J
Gp594c	346402:347511 (r)	39350	369	Ig domain-containing protein	QDF13640.1	4e-166 (63.69)	100		Escherichia phage vB_EcoM_phAPEC6
Gp595c	347584:348732 (r)	41960	382	regulator of chromosome condensation	WGM49826.1	0.0 (92.41)	100		Escherichia phage vB_Ec-M-J
Gp596	348835:349062 (f)	8359	75	hypothetical protein	QXN76576.1	3e-43 (97.33)	100		Escherichia phage BF17
Gp597	349059:349271 (f)	8405	70	hypothetical protein G17_00068	QBO61564.1	1e-32 (83.58)	100		Escherichia phage vB_EcoM_G17
Gp598	349273:349458 (f)	7705	61	hypothetical protein vBEcoMphAPEC6_02890	WAE77428.1	1e-32 (91.80)	100		Escherichia phage ph0011
Gp599	349470:349682 (f)	8211	70	hypothetical protein Ecwhy1_514	QAY00787.1	2e-42 (100.00)	100		Escherichia phage Ecwhy_1
Gp600	349679:349864 (f)	7543	61	hypothetical protein vBEcoMphAPEC6_02900	WAE77430.1	2e-35 (98.36)	100		Escherichia phage ph0011
Gp601	349875:350057 (f)	7328	60	hypothetical protein	QXN76578.1	2e-34 (100.00)	100		Escherichia phage BF17
Gp602	350066:350251 (f)	7304	61	hypothetical protein Ecwhy1_513	QAY00786.1	4e-36 (100.00)	100		Escherichia phage Ecwhy_1
Gp603	350260:350439 (f)	7494	59	hypothetical protein EO157G_2320	BBM61821.1	9e-33 (98.31)	100		Escherichia phage SP27
Gp604	350447:350635 (f)	7403	62	hypothetical protein EcMJ_590	WGM49832.1	6e-36 (100.00)	100		Escherichia phage vB_Ec-M-J
Gp605	350644:350823 (f)	7000	59	hypothetical protein UES1_218	UTS53586.1	5e-30 (88.14)	100		Escherichia phage UE-S1
Gp606	350834:351493 (f)	25581	219	hypothetical protein Ecwhy1_511	QAY00784.1	3e-156 (98.63)	100		Escherichia phage Ecwhy_1
Gp607	351486:351659 (f)	6704	57	hypothetical protein Ecwhy1_510	QAY00783.1	8e-32 (100.00)	100		Escherichia phage Ecwhy_1
Gp608	351668:351859 (f)	7879	63	hypothetical protein G17_00058	QBO61554.1	4e-36 (98.41)	100		Escherichia phage vB_EcoM_G17
Gp609	352045:352233 (f)	7559	62	hypothetical protein	QXN76583.1	9e-38 (100.00)	100		Escherichia phage BF17
Gp610	352244:352450 (f)	8018	68	hypothetical protein vBEcoMphAPEC6_00020	WAE76861.1	1e-42 (98.53)	100		Escherichia phage ph0011
Gp611	352494:352634 (f)	5637	46	hypothetical protein	QXN76585.1	7e-23 (97.83)	100		Escherichia phage BF17
Gp612	353029:353220 (f)	7584	63	hypothetical protein Ecwhy1_508	QAY00781.1	6e-37 (96.83)	100		Escherichia phage Ecwhy_1

Gp613	353232:353438 (f)	8416	68	hypothetical protein Ecwhy1_507	QAY00780.1	1e-41 (100.00)	100		Escherichia phage Ecwhy_1
Gp614c	354759:355058 (r)	11299	99	hypothetical protein P3ECOSTME9A_CDS0004	XDC03225.1	2e-60 (96.97)	100		Escherichia phage vB_EcoM_E9.1
Gp615	355429:355848 (f)	16045	139	hypothetical protein Ecwhy1_506	QAY00779.1	1e-99 (100.00)	100		Escherichia phage Ecwhy_1
Gp616	355942:356181 (f)	9006	79	hypothetical protein UES1_227	UTS53595.1	8e-51 (98.73)	100		Escherichia phage UE-S1
Gp617	356268:356534 (f)	10170	88	hypothetical protein PSLUR01_00170	SCA80147.1	1e-54 (98.86)	100		Escherichia phage vB_Eco_slurp01
Gp618	356622:356852 (f)	8933	76	hypothetical protein EJP02_517	WEM33578.1	6e-46 (97.37)	100		Escherichia phage EJP2
Gp619	357018:357128 (f)	4272	36	hypothetical protein ELP22_0010	XAJ02068.1	5e-15 (97.22)	100		Escherichia phage BAU.Micro_ELP-22
Gp620	357280:357891 (f)	23518	203	hypothetical protein PSLUR01_00172	SCA80149.1	3e-144 (99.51)	100		Escherichia phage vB_Eco_slurp01
Gp621	357984:358148 (f)	5983	54	hypothetical protein JR323_gp138	YP_009985993.1	5e-12 (62.75)	100		Escherichia phage nepoznato
Gp622	358207:358458 (f)	9102	83	hypothetical protein EO157G_2500	BBM61839.1	2e-54 (100.00)	100		Escherichia phage SP27
Gp623	358703:358939 (f)	8735	78	phage protein	BBM61840.1	8e-51 (98.72)	100		Escherichia phage SP27
Gp624	359024:359323 (f)	10816	99	Protein spackle; APOBEC, deaminase, hydrolase, viral protein; 1.52A	6X6O_A			3.9(69.84)	Escherichia virus T4
Gp625	359345:359575 (f)	8065	76	hypothetical protein Ecwhy1_494	QAY00768.1	2e-44 (100.00)	100		Escherichia phage Ecwhy_1
Gp626	359809:360291 (f)	17469	160	hypothetical protein Ecwhy1_493	QAY00767.1	5e-107 (98.75)	100		Escherichia phage Ecwhy_1
Gp627	360567:360719 (f)	5627	50	hypothetical protein	WOL23061.1	4e-27 (98.00)	100		Escherichia phage vB_EcoM_JNE01
Gp628	360790:361017 (f)	8201	75	hypothetical protein ACQ29_gp266	YP_009150580.1	2e-45 (98.67)	100		Escherichia phage PBECO4
Gp629	361103:361246 (f)	5314	47	hypothetical protein vBEcoMphAPEC6_gp526c	QDF14149.1	7e-25 (97.87)	100		Escherichia phage vB_EcoM_phAPEC6
Gp630	361336:361659 (f)	11951	107	hypothetical protein	WOL23064.1	5e-72 (99.07)	100		Escherichia phage vB_EcoM_JNE01
Gp631	361740:362009 (f)	10230	89	hypothetical protein EO157G_2580	BBM61847.1	2e-59 (98.88)	100		Escherichia phage SP27
Gp632	362061:362333 (f)	10161	90	hypothetical protein Ecwhy1_488	QAY00762.1	2e-58 (100.00)	100		Escherichia phage Ecwhy_1
Gp633	362339:362548 (f)	7708	69	hypothetical protein	WIL00536.1	7e-40 (97.10)	100		Escherichia phage vB_EcoM_CRJP21
Gp634	362618:362764 (f)	5368	48	hypothetical protein G17_00034	QBO61530.1	6e-23 (95.83)	100		Escherichia phage vB_EcoM_G17

Gp635	362846:362998 (f)	5809	50	hypothetical protein A4_286	USL83362.1	6e-28 (98.00)	100		Escherichia phage A4
Gp636	362995:363084 (f)	3169	29	hypothetical protein vBEcoMphAPEC6_ gp520c	QDF14143.1	2e-20 (93.10)	100		Escherichia phage vB_EcoM_phAPEC6
Gp637	363085:363228 (f)	5216	47	hypothetical protein EcMJ_023	WGM49266 .1	2e-23 (97.87)	100		Escherichia phage vB_Ec-M-J
Gp638	363439:363780 (f)	12593	113	hypothetical protein	WIL00539.1	2e-75 (100.00)	100		Escherichia phage vB_EcoM_CRJP21
Gp639	363877:364089 (f)	7895	70	hypothetical protein	QXN76009. 1	2e-41 (97.14)	100		Escherichia phage BF17
Gp640	364173:364352 (f)	6943	59	hypothetical protein	AXC37039. 1	4e-33 (100.00)	100		Escherichia phage UB
Gp641	364443:364586 (f)	5246	47	hypothetical protein PBI_121Q_291	YP_0091018 78.1	3e-23 (95.74%)	100		Escherichia phage 121Q
Gp642	364692:364889 (f)	7669	65	hypothetical protein Ecwhy1_481	QAY00755. 1	2e-36 (100.00)	100		Escherichia phage Ecwhy_1
Gp643	365073:365219 (f)	5460	48	hypothetical protein	WOL23076. 1	2e-23 (97.92)	100		Escherichia phage vB_EcoM_JNE01
Gp644	365219:365476 (f)	9741	85	DksA-like zinc-finger protein	XHY53928. 1	3e-54 (96.47)	100		Escherichia phage JP4
Gp645	365540:365896 (f)	13312	118	GNAT family N-acetyltransferase	YP_0091018 82.1	3e-77 (94.92)	100		Escherichia phage 121Q
Gp646	365975:366163 (f)	6806	62	hypothetical protein ACQ29_gp252	YP_0091505 66.1	3e-36 (100.00)	100		Escherichia phage PBECO4
Gp647	366191:366514 (f)	12168	107	hypothetical protein vBEcoMphAPEC6_ 00180	WAE76893. 1	2e-74 (100.00)	100		Escherichia phage ph0011
Gp648	366598:366810 (f)	8185	70	hypothetical protein UES1_256	UTS53624.1	3e-44 (98.57)	100		Escherichia phage UE-S1
Gp649	366898:367071 (f)	6600	57	hypothetical protein Ecwhy1_475	QAY00749. 1	6e-33 (100.00)	100		Escherichia phage Ecwhy_1
Gp650	367068:367217 (f)	5751	49	hypothetical protein UES1_258	UTS53626.1	2e-26 (97.96)	100		Escherichia phage UE-S1
Gp651	367533:367808 (f)	10650	91	hypothetical protein Ecwhy1_473	QAY00747. 1	4e-59 (100.00)	100		Escherichia phage Ecwhy_1
Gp652	367897:368079 (f)	6705	60	hypothetical protein	WOL23085. 1	9e-35 98.33%	100		Escherichia phage vB_EcoM_JNE01
Gp653	368167:368490 (f)	12494	107	hypothetical protein EcMJ_037	WGM49280 .1	4e-71 (98.13)	100		Escherichia phage vB_Ec-M-J
Gp654	368586:368981 (f)	14942	131	hypothetical protein Ecwhy1_470	QAY00745. 1	1e-90 (100.00)	100		Escherichia phage Ecwhy_1
Gp655	369043:369297 (f)	9870	84	hypothetical protein Ecwhy1_469	QAY00744. 1	1e-51 (100)	100		Escherichia phage Ecwhy_1
Gp656	369294:369569 (f)	10748	91	hypothetical protein UES1_263	UTS53631.1	7e-56 (93.41)	100		Escherichia phage UE-S1
Gp657	369570:369869 (f)	11046	99	hypothetical protein UES1_264	UTS53632.1	3e-64 (98.99)	100		Escherichia phage UE-S1
Gp658	369901:370485 (f)	22372	194	hypothetical protein Ecwhy1_466	QAY00741. 1	1e-142(100.00)	100		Escherichia phage Ecwhy_1

Gp659	370576:370695 (f)	4662	39	hypothetical protein UES1_266	UTS53634.1	3e-08 (84.85)	100		Escherichia phage UE-S1
Gp660	370707:370928 (f)	8334	73	hypothetical protein A4_305	USL83381.1	2e-46 (98.63)	100		Escherichia phage A4
Gp661	371012:371416 (f)	15132	134	hypothetical protein vBEcoMphAPEC6_00245	WAE76906.1	4e-93 (97.76)	100		Escherichia phage ph0011
Gp662	371506:371778 (f)	9898	90	hypothetical protein vBEcoMphAPEC6_00250	WAE76907.1	1e-56 (96.67)	100		Escherichia phage ph0011
Gp663	371775:371966 (f)	7320	63	hypothetical protein	WOL23095.1	1e-36 (96.83)	100		Escherichia phage vB_EcoM_JNE01
Gp664	372054:372317 (f)	9488	87	hypothetical protein A4_309	USL83385.1	5e-55 (98.85)	100		Escherichia phage A4
Gp665	372385:372609 (f)	8760	74	hypothetical protein A4_310	USL83386.1	5e-46 (98.65)	100		Escherichia phage A4
Gp666	372686:372943 (f)	9768	85	hypothetical protein vBEcoMphAPEC6_00270	WAE76911.1	1e-57 (100.00)	100		Escherichia phage ph0011
Gp667	373034:373234 (f)	7320	66	hypothetical protein vBEcoMphAPEC6_00275	WAE76912.1	4e-39 (98.48)	100		Escherichia phage ph0011
Gp668	373480:373812 (f)	12478	110	hypothetical protein UES1_273	UTS53641.1	1e-74 (100.00)	100		Escherichia phage UE-S1
Gp669	373869:374303 (f)	16040	144	hypothetical protein vBEcoMphAPEC6_00285	WAE76914.1	3e-102 (100.00)	100		Escherichia phage ph0011
Gp670	374372:374581 (f)	7620	69	hypothetical protein vBEcoMphAPEC6_00290	WAE76915.1	1e-41 (100.00)	100		Escherichia phage ph0011

* The gene products significantly different in phage Ecwhy_1 (**Table S3**) are highlighted in orange, and those completely absent in Ecwhy_1 (**Table S4**) are highlighted in light grey.

Table S3. Gene products significantly different between phages fEgEco12 and Ecwhy_1

Gp ID	Gp#	% id	Annotation if not hypothetical protein
XBS49145.1	063	30.1	
XBS49151.1	069	53.4	
XBS49159.1	077	36.1	
XBS49167.1	085	72.6	
XBS49179.1	097	38.8	
XBS49196.1	114	51.0	
XBS49665.1	583	29.6	chromosome condensation regulator
XBS49666.1	584	30.9	
XBS49667.1	585	63.7	
XBS49675.1	593	25.4	chromosome condensation regulator
XBS49679.1	597	36.8	

Table S4. Phage fEgEco12 proteins completely absent in phage Ecwhy_1

Gp ID	Gp#	Annotation if not hypothetical protein
XBS49087.1	05	
XBS49089.1	07	
XBS49095.1	013	
XBS49097.1	015	
XBS49103.1	021	
XBS49104.1	022	
XBS49105.1	023	
XBS49111.1	029	
XBS49120.1	038	
XBS49127.1	045	
XBS49171.1	089	
XBS49190.1	108	
XBS49197.1	115	
XBS49242.1	160	
XBS49254.1	172	
XBS49259.1	177	
XBS49269.1	187	
XBS49275.1	193	
XBS49278.1	196	
XBS49295.1	213	
XBS49296.1	214	
XBS49299.1	217	
XBS49309.1	227	
XBS49320.1	238	
XBS49338.1	256	
XBS49339.1	257	
XBS49420.1	338	
XBS49422.1	340	
XBS49423.1	341	
XBS49460.1	378	
XBS49464.1	382	
XBS49563.1	481	

XBS49570.1	488	
XBS49574.1	492	
XBS49601.1	519	RNA polymerase sigma factor
XBS49604.1	522	HNH endonuclease
XBS49612.1	530	
XBS49639.1	557	
XBS49652.1	570	HNH endonuclease
XBS49668.1	586	
XBS49680.1	598	
XBS49682.1	600	
XBS49683.1	601	
XBS49685.1	603	
XBS49687.1	605	
XBS49690.1	608	
XBS49691.1	609	
XBS49693.1	611	
XBS49696.1	614	
XBS49701.1	619	
XBS49703.1	621	
XBS49709.1	627	
XBS49711.1	629	
XBS49717.1	635	
XBS49718.1	636	
XBS49719.1	637	
XBS49725.1	643	
XBS49734.1	652	
XBS49741.1	659	
